# Supplementary material for: Molecular Breeding for Incorporation of Submergence Tolerance and Durable Bacterial Blight Resistance into the Popular Rice Variety ‘Ranidhan’
Source: Biomolecules. 2023 Jan 18;13(2):198. doi: 10.3390/biom13020198 (PMC9953461; doi:10.3390/biom13020198)
Supplement: Supplementary file 1 [file biomolecules-13-00198-s001.zip › biomolecules-2155745-supplementary.pdf]

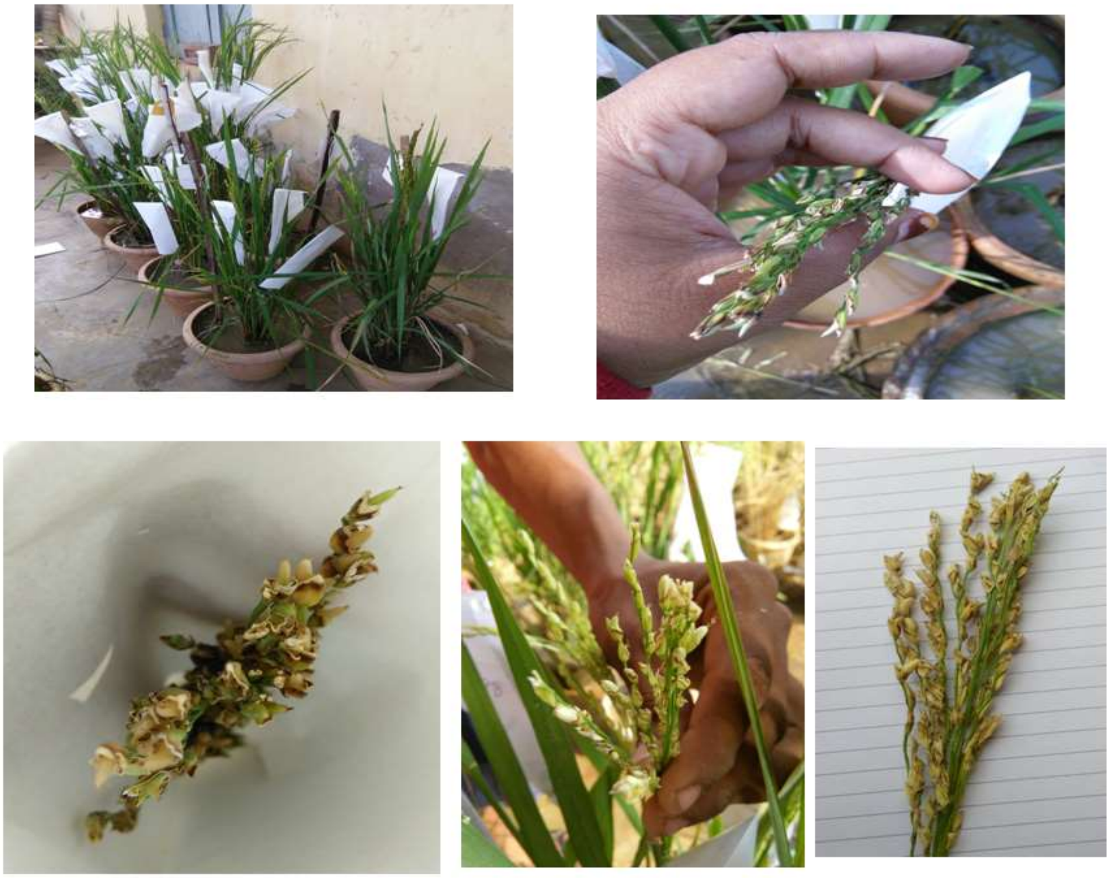

Supplementary figure S1. Hyridizatioon n and generation of cross seeds of recipient and doonor parents for transfer of submergence tolerance e and bacterial blight resistance in variety, Ranidhan..

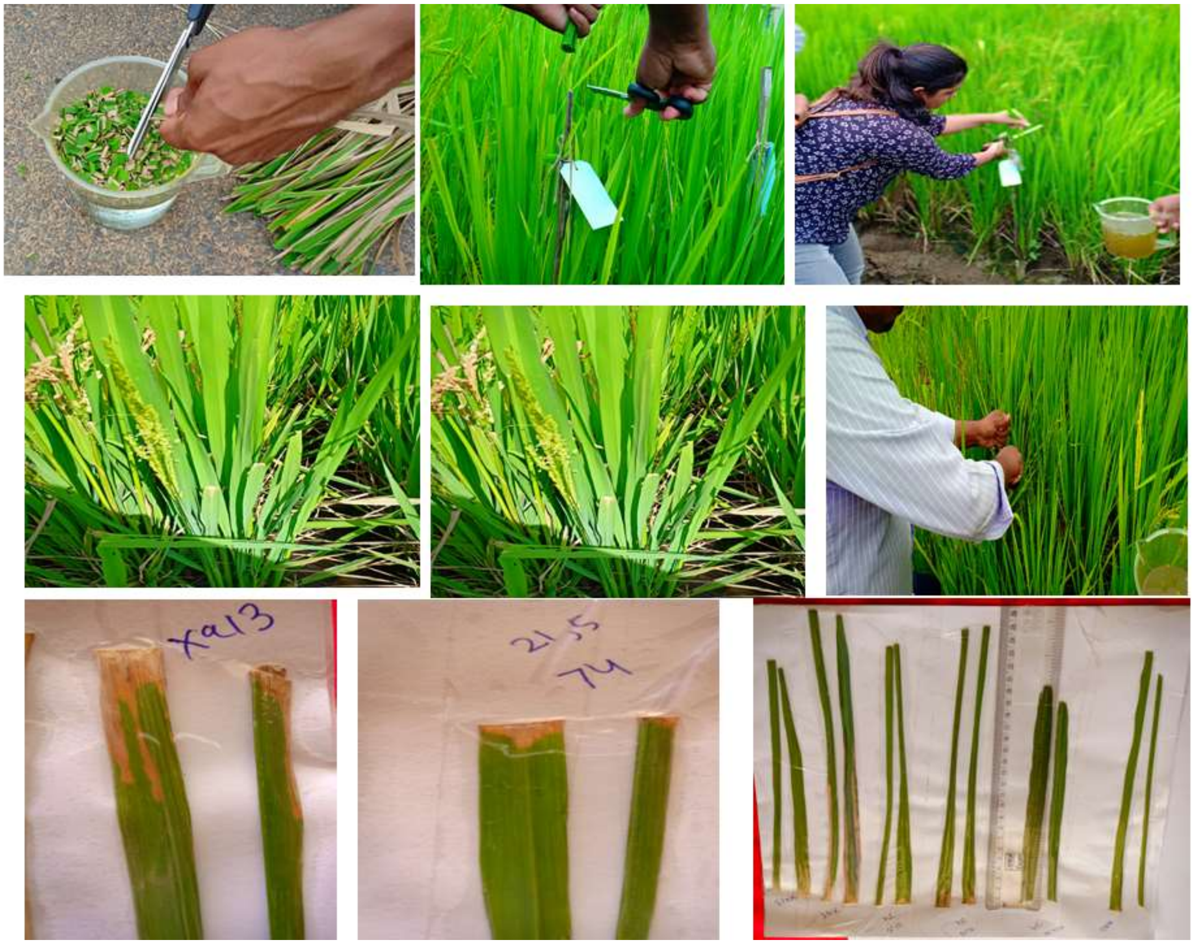

Supplementary figure S2. Clip inocullaation of bacterial blight strains into the pyramided   aand parental lines.

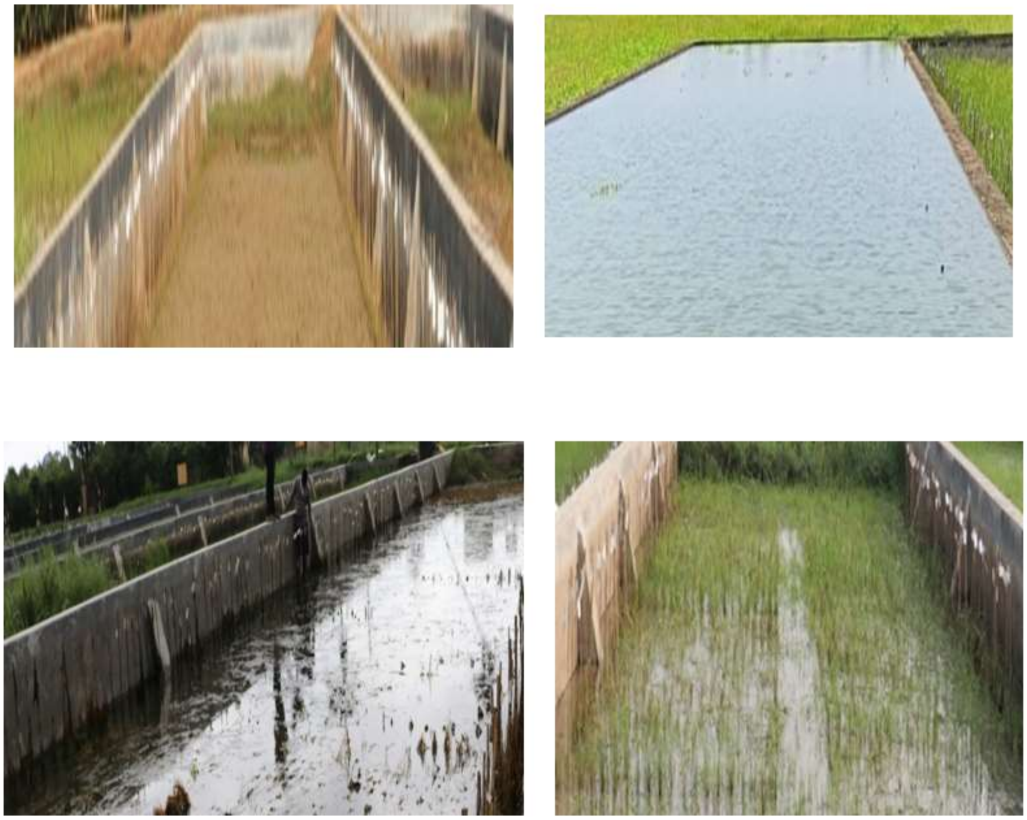

Supplementary figure S3. Screening ffor the submergence tolerance of the pyramided annd parental lines in the screening tank.

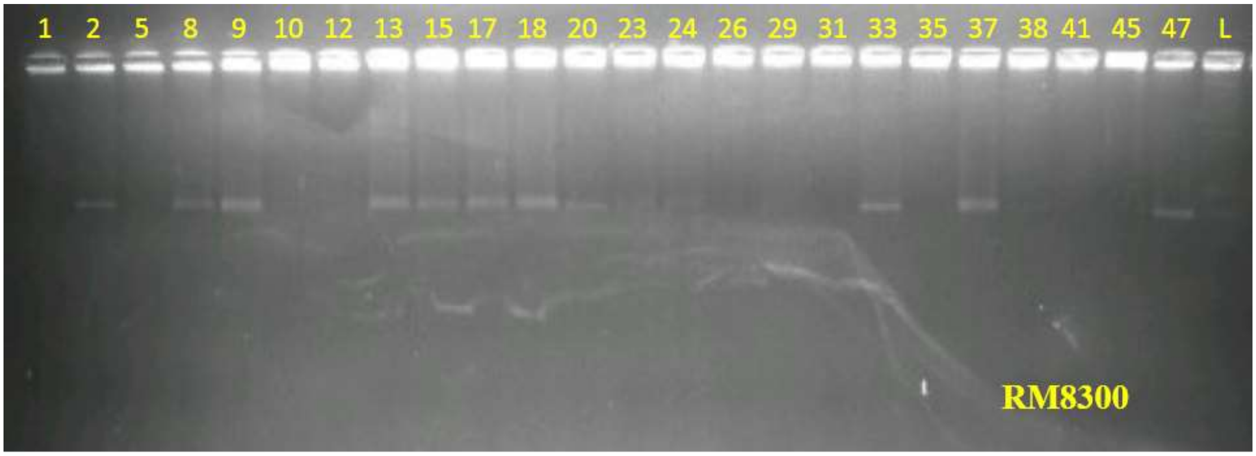
1 2 5 8 9 10 12 13 15 17 18 20 23 24 26 29 31 33 35 37 38 41 45 47 L
RM8300

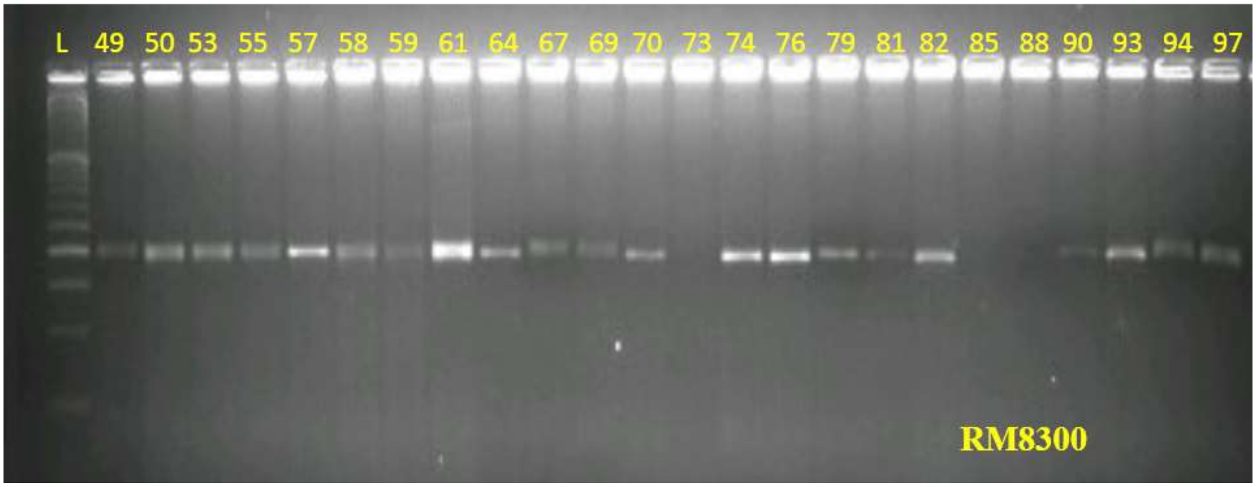
L 49 50 53 55 57 58 59 61 64 67 69 70 73 74 76 79 81 82 85 88 90 93 94 97
RM8300

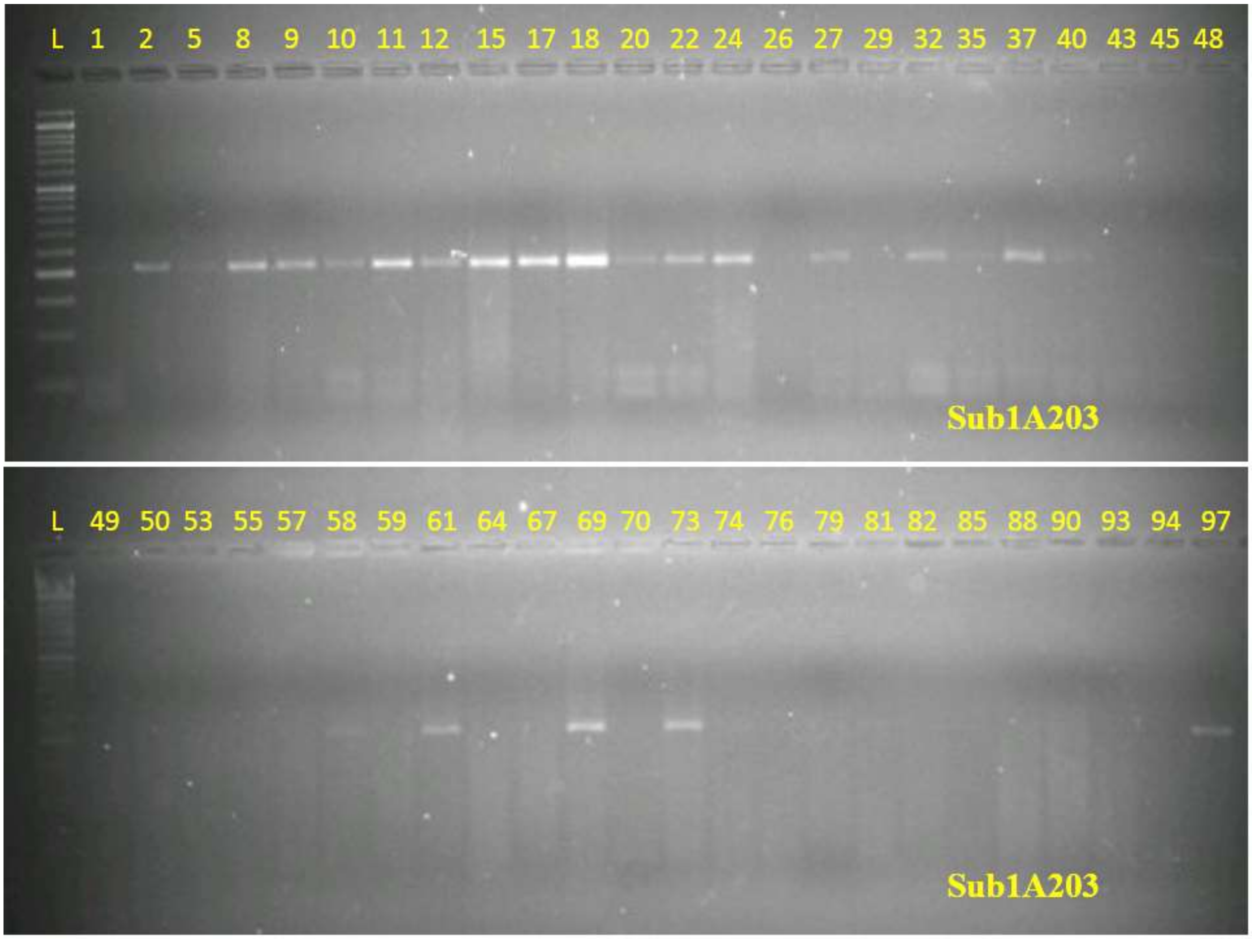
L 1 2 5 8 9 10 11 12 15 17 18 20 22 24 26 27 29 32 35 37 40 43 45 48
Sub1A203
L 49 50 53 55 57 58 59 61 64 67 69 70 73 74 76 79 81 82 85 88 90 93 94 97
Sub1A203

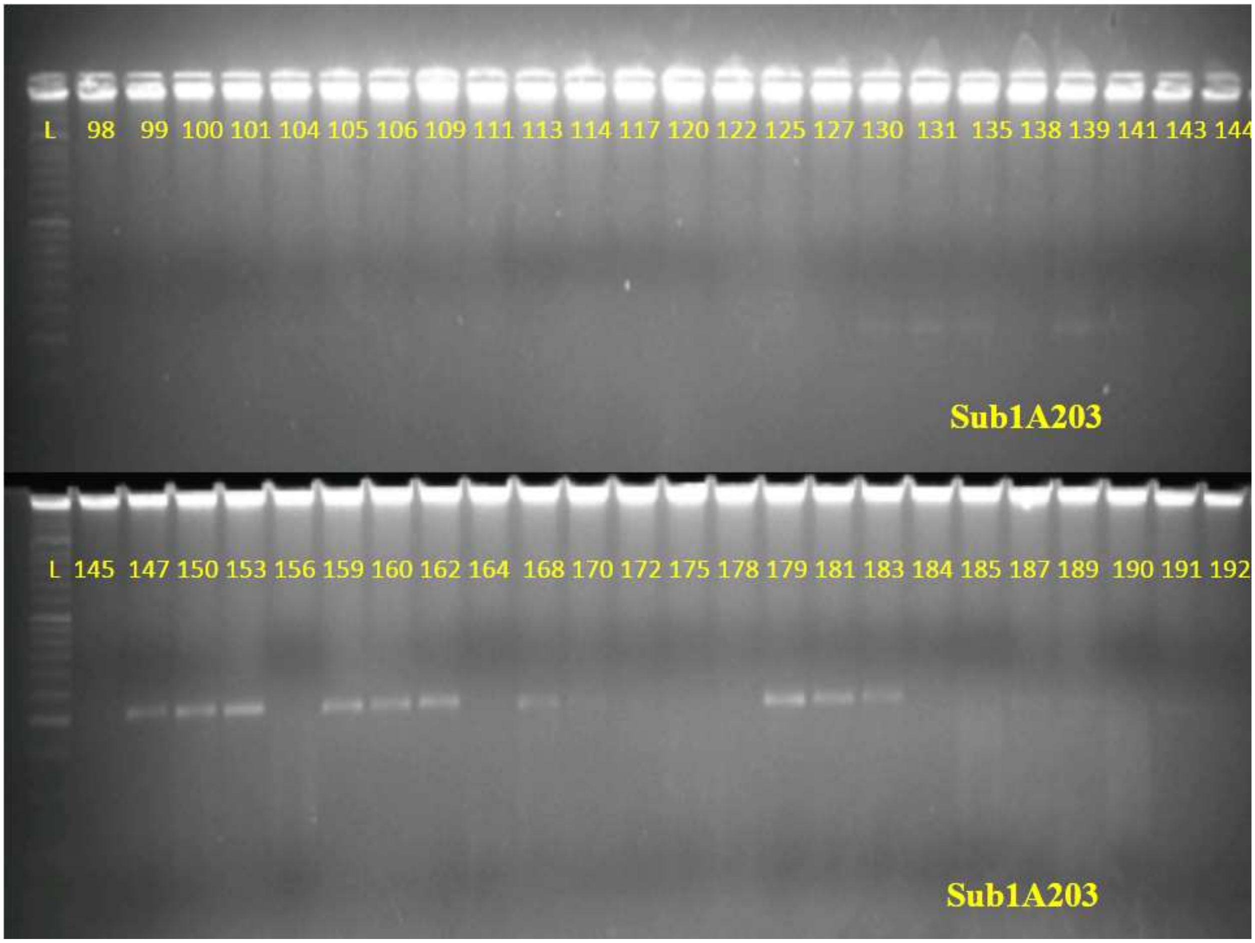
L 98 99 100 101 104 105 106 109 111 113 114 117 120 122 125 127 130 131 135 138 139 141 143 144
Sub1A203
L 145 147 150 153 156 159 160 162 164 168 170 172 175 178 179 181 183 184 185 187 189 190 191 192
Sub1A203

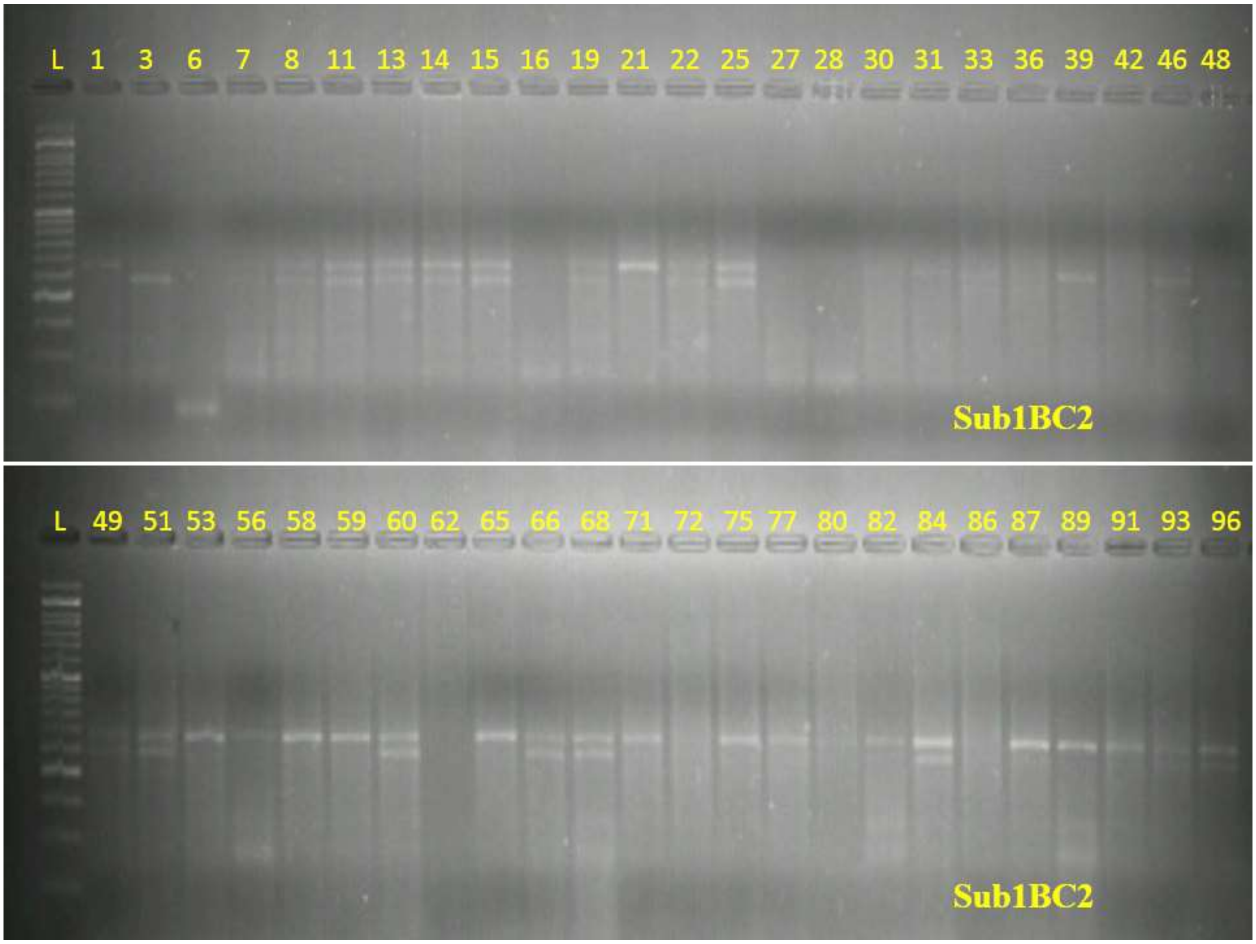
L 1 3 6 7 8 11 13 14 15 16 19 21 22 25 27 28 30 31 33 36 39 42 46 48
Sub1BC2
L 49 51 53 56 58 59 60 62 65 66 68 71 72 75 77 80 82 84 86 87 89 91 93 96
Sub1BC2

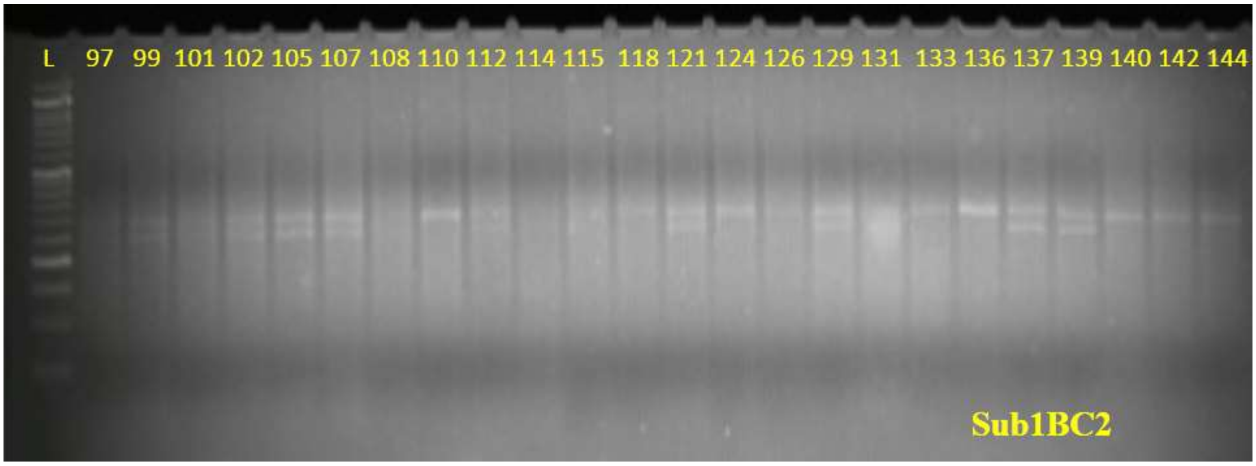
L 97 99 101 102 105 107 108 110 112 114 115 118 121 124 126 129 131 133 136 137 139 140 142 144
Sub1BC2

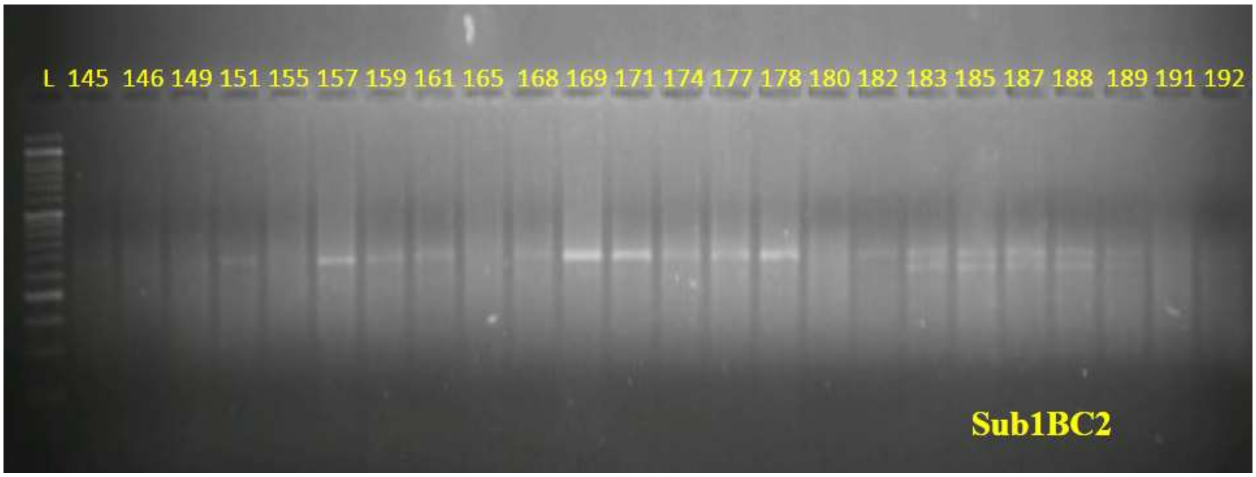
L 145 146 149 151 155 157 159 161 165 168 169 171 174 177 178 180 182 183 185 187 188 189 191 192
Sub1BC2

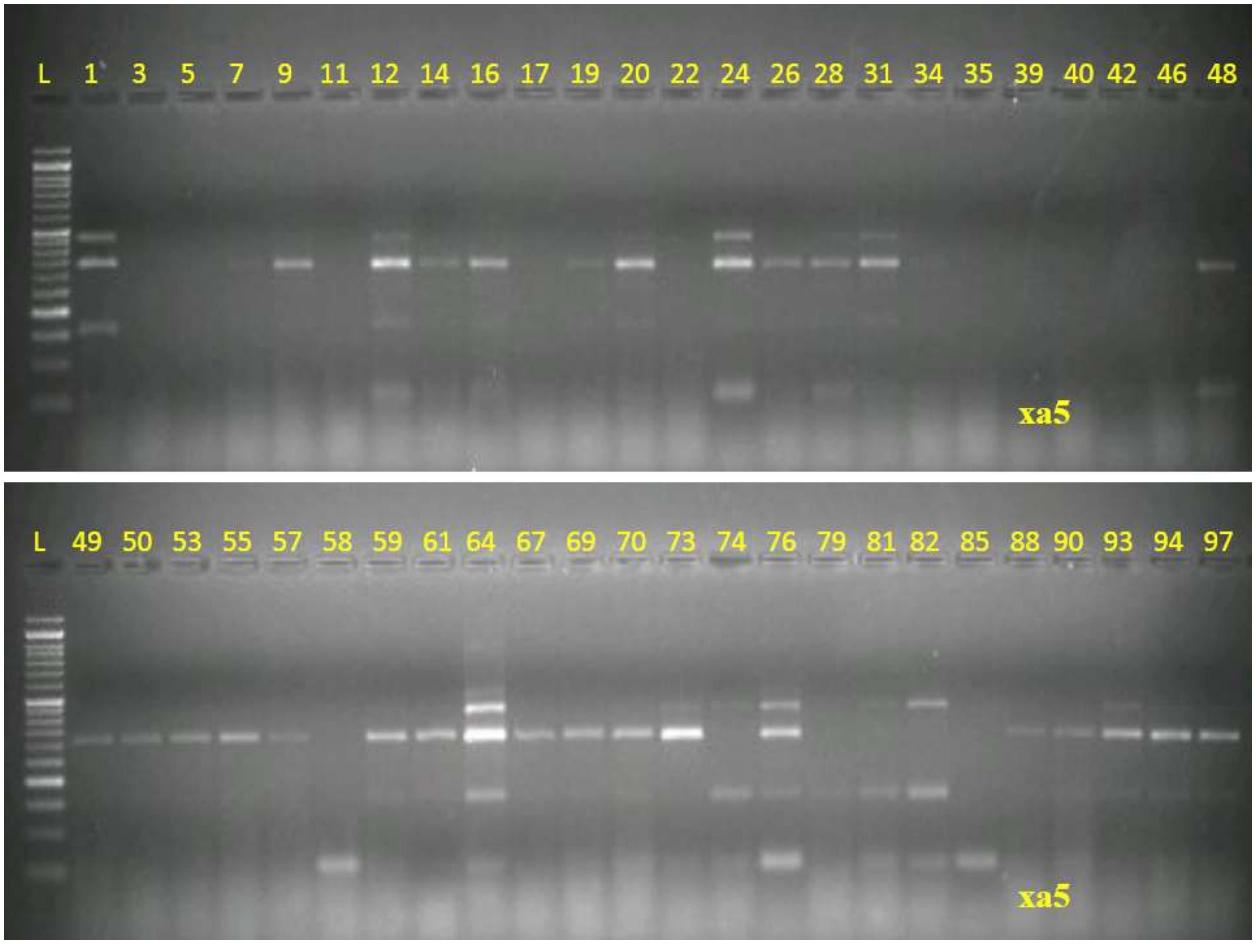
L 1 3 5 7 9 11 12 14 16 17 19 20 22 24 26 28 31 34 35 39 40 42 46 48
xa5
L 49 50 53 55 57 58 59 61 64 67 69 70 73 74 76 79 81 82 85 88 90 93 94 97
xa5

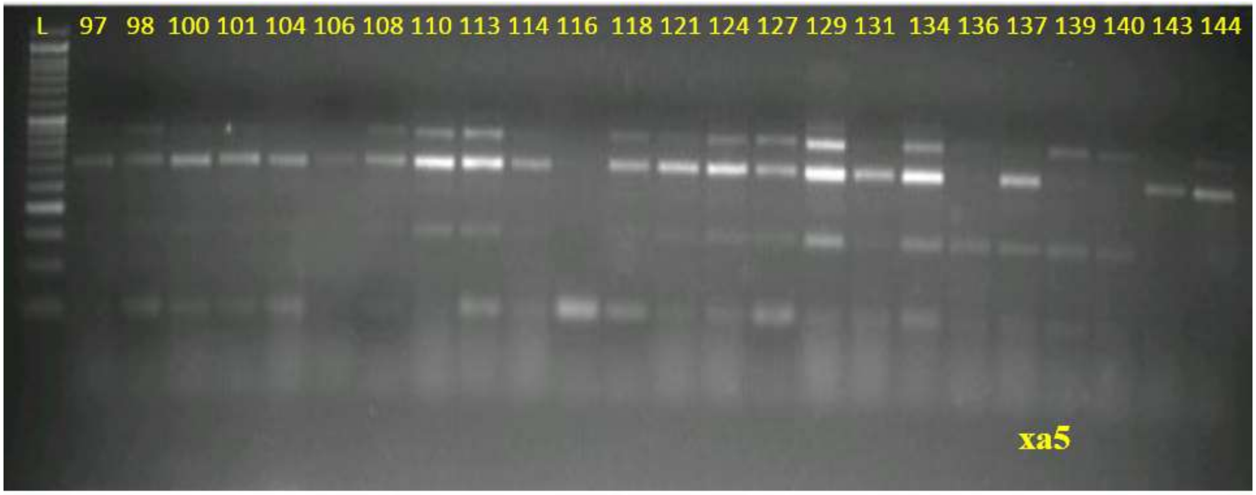
L 97 98 100 101 104 106 108 110 113 114 116 118 121 124 127 129 131 134 136 137 139 140 143 144
xa5

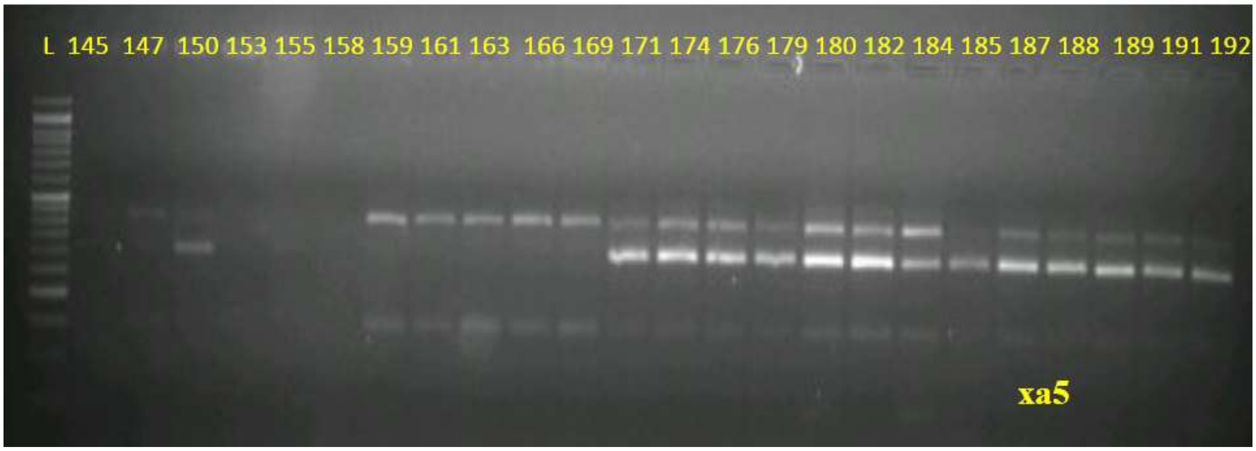
L 145 147 150 153 155 158 159 161 163 166 169 171 174 176 179 180 182 184 185 187 188 189 191 192
xa5

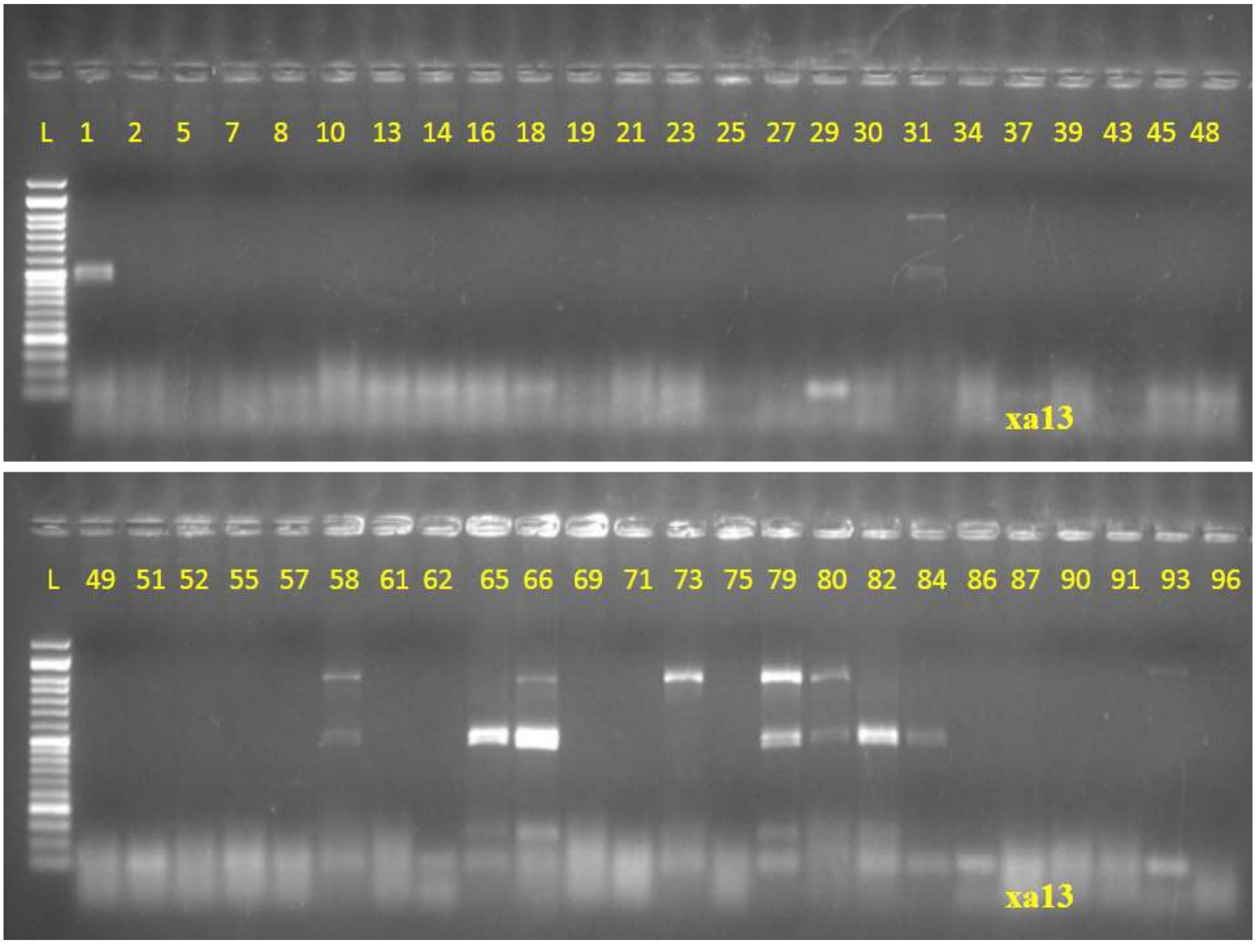
L 1 2 5 7 8 10 13 14 16 18 19 21 23 25 27 29 30 31 34 37 39 43 45 48
xa13
L 49 51 52 55 57 58 61 62 65 66 69 71 73 75 79 80 82 84 86 87 90 91 93 96
xa13

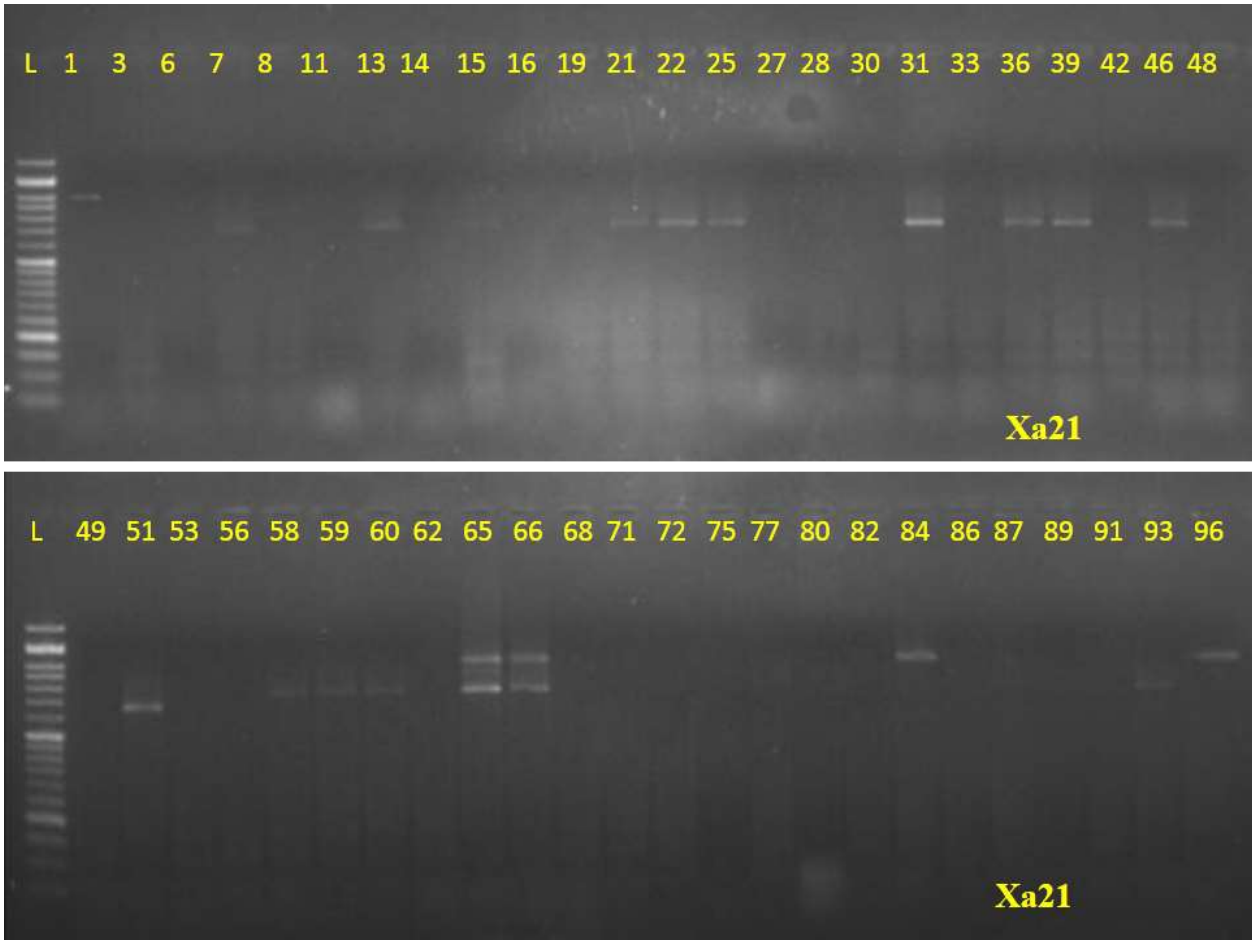
L 1 3 6 7 8 11 13 14 15 16 19 21 22 25 27 28 30 31 33 36 39 42 46 48
Xa21
L 49 51 53 56 58 59 60 62 65 66 68 71 72 75 77 80 82 84 86 87 89 91 93 96
Xa21

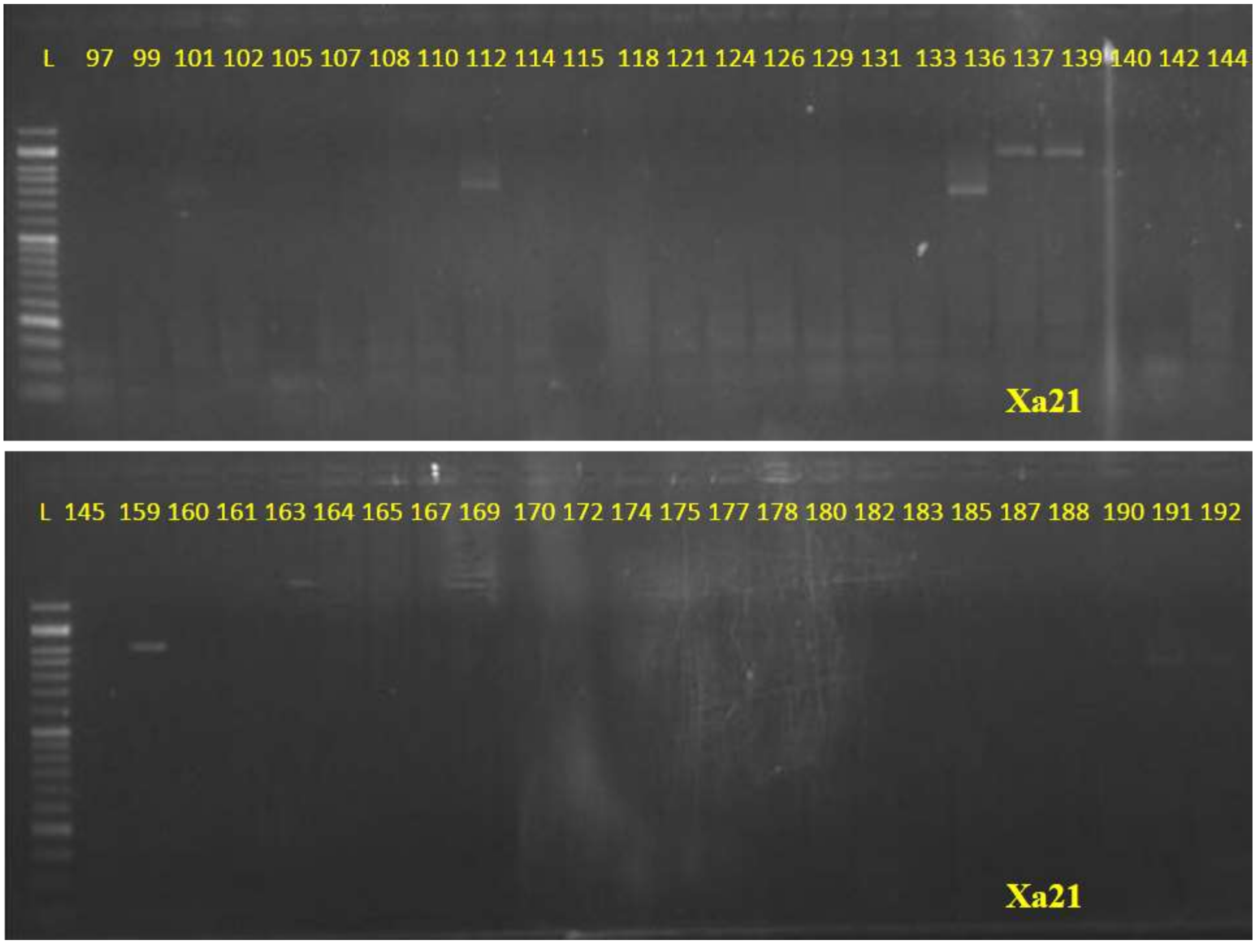

Supplementary figure S4. Molecular screening of BC1F1 progenies for submergence tolerance and bacterial blight resistance in rice variety, Ranidhan background

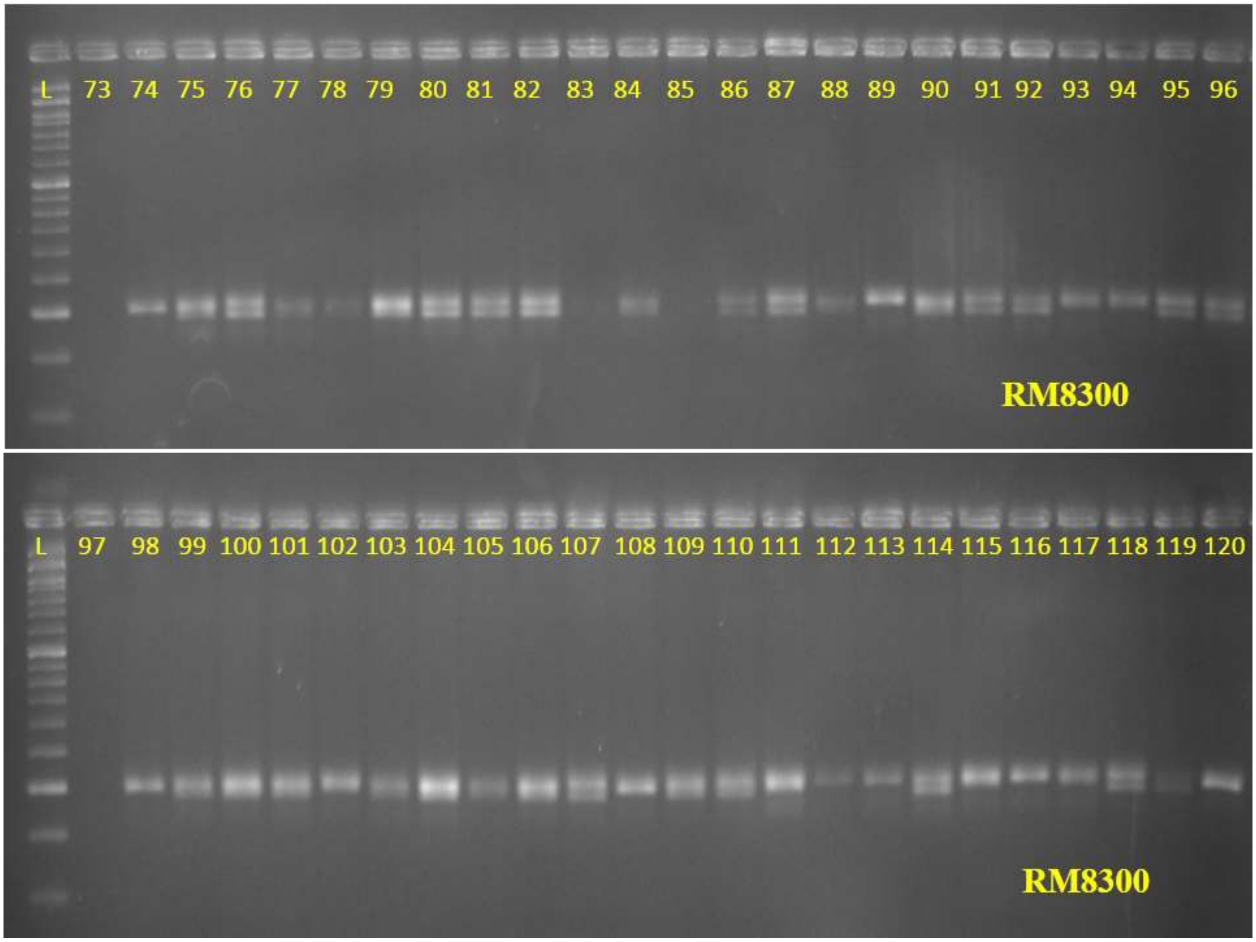

Supplementary figure S5. Molecular screening of BC2F1 progenies in rice variety, Ranidhan background for submergence tolerance and bacterial blight resistance

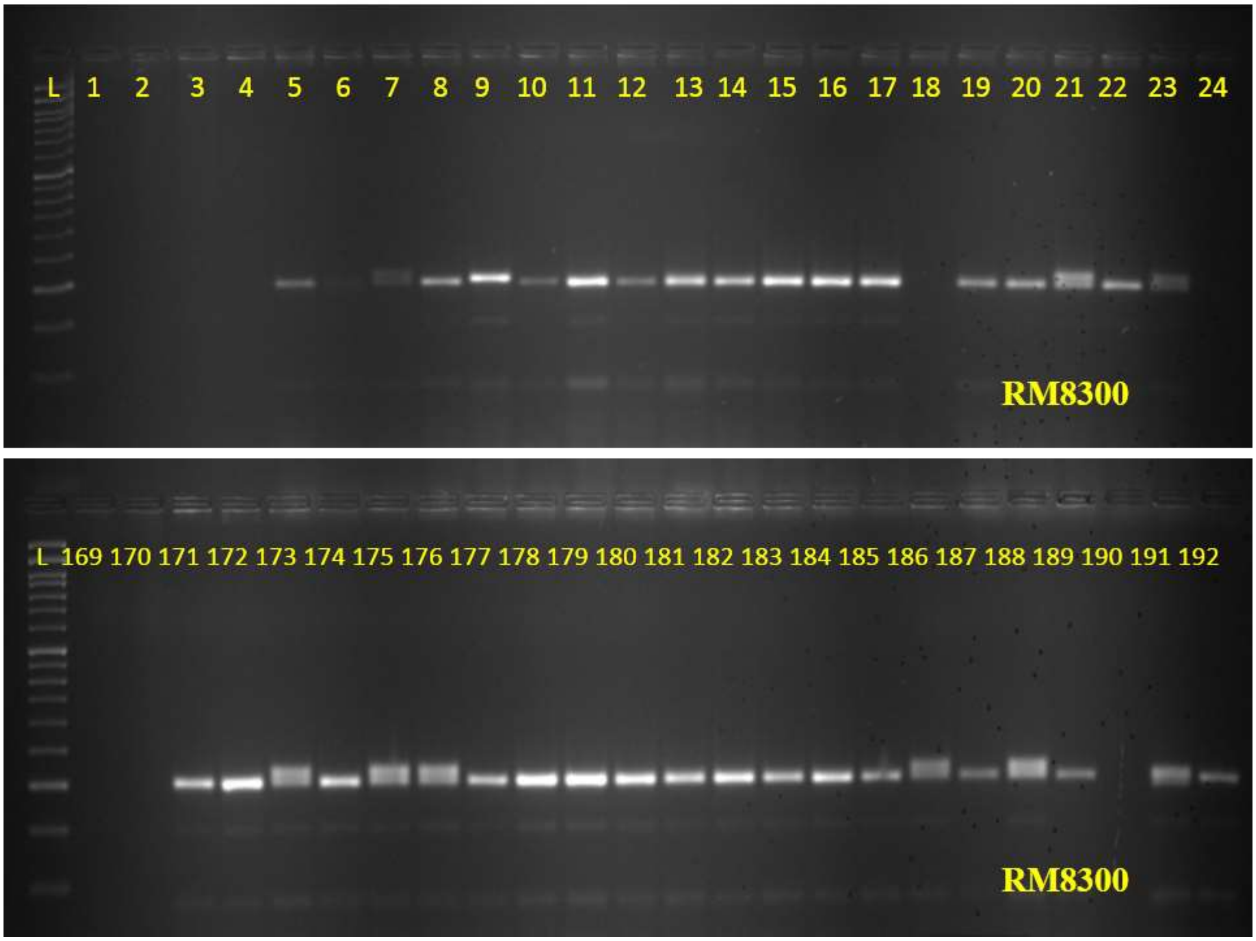
L 1 2 3 4 5 6 7 8 9 10 11 12 13 14 15 16 17 18 19 20 21 22 23 24
RM8300
L 169 170 171 172 173 174 175 176 177 178 179 180 181 182 183 184 185 186 187 188 189 190 191 192
RM8300

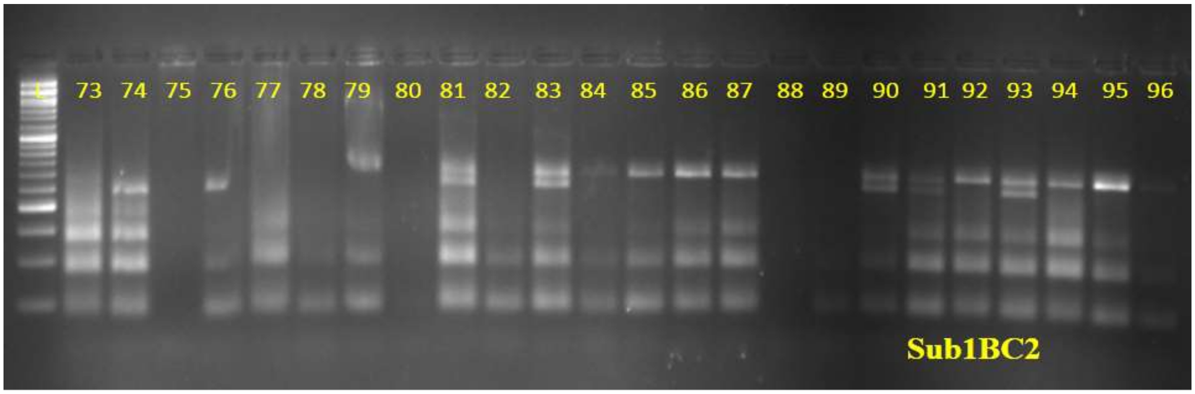
73 74 75 76 77 78 79 80 81 82 83 84 85 86 87 88 89 90 91 92 93 94 95 96
Sub1BC2

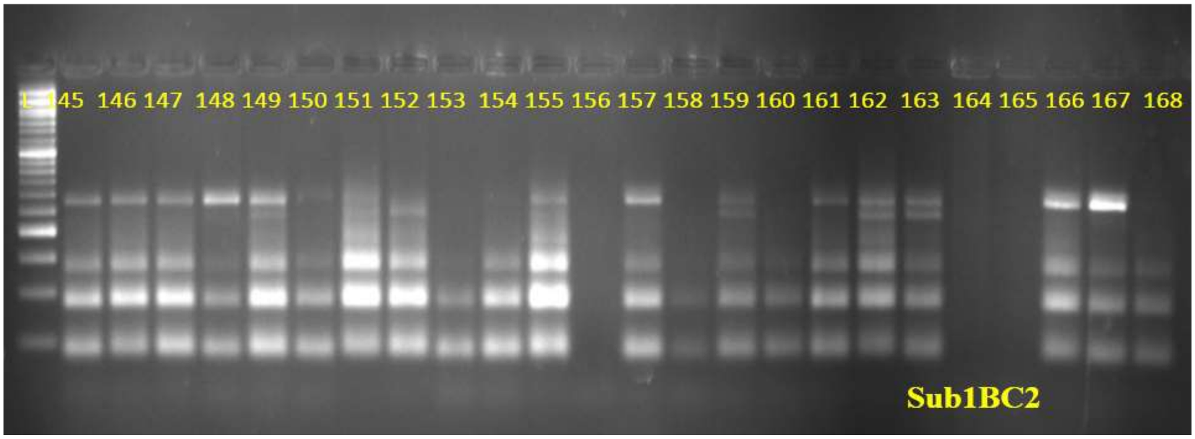
145 146 147 148 149 150 151 152 153 154 155 156 157 158 159 160 161 162 163 164 165 166 167 168
Sub1BC2

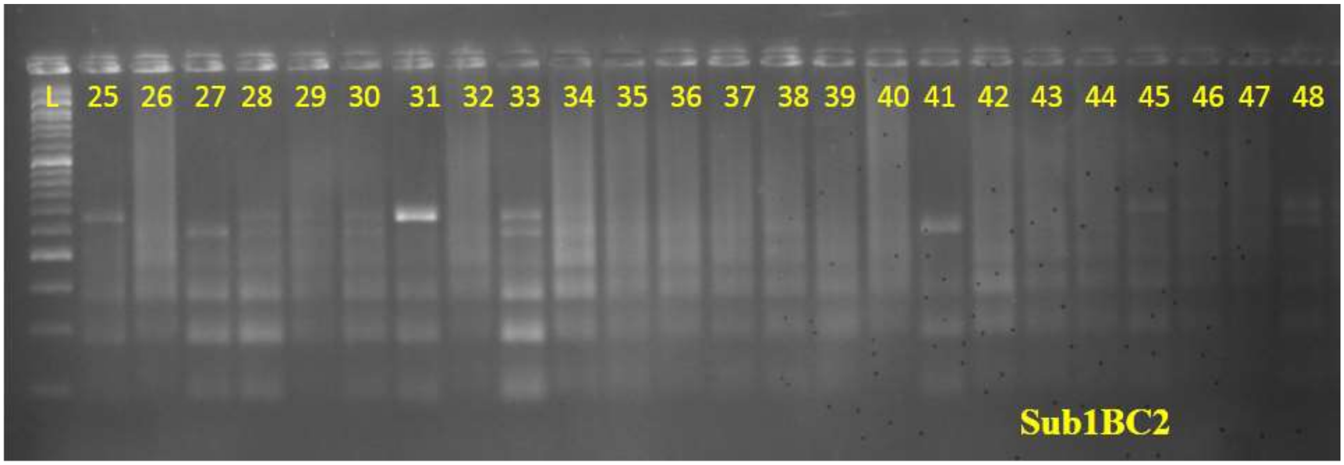
L 25 26 27 28 29 30 31 32 33 34 35 36 37 38 39 40 41 42 43 44 45 46 47 48
Sub1BC2

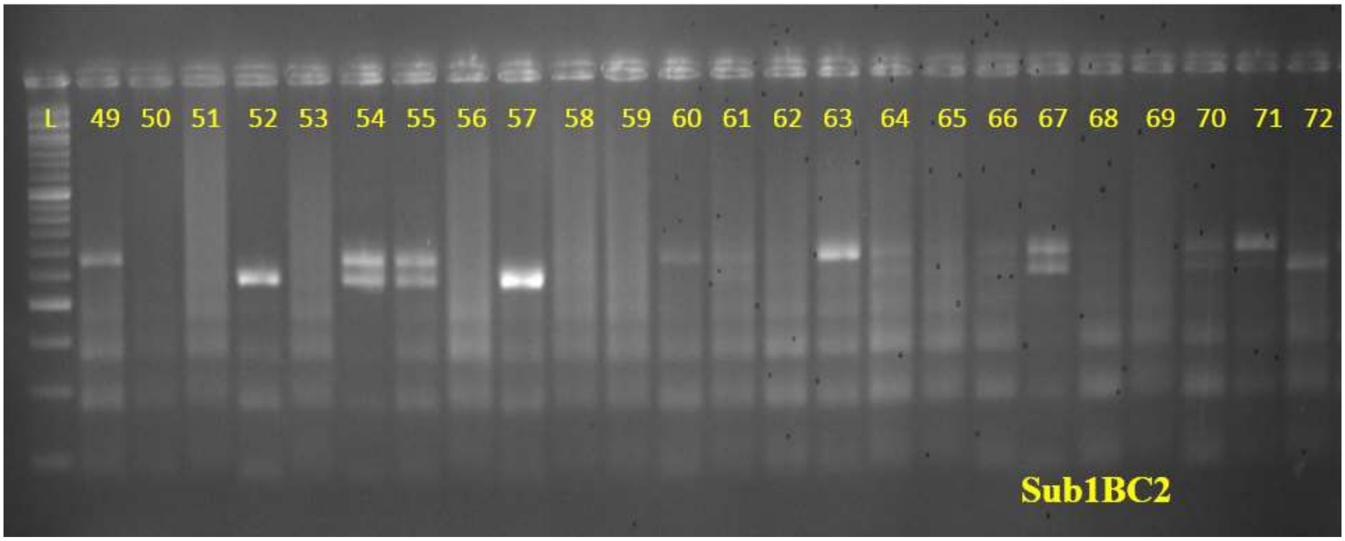
L 49 50 51 52 53 54 55 56 57 58 59 60 61 62 63 64 65 66 67 68 69 70 71 72
Sub1BC2

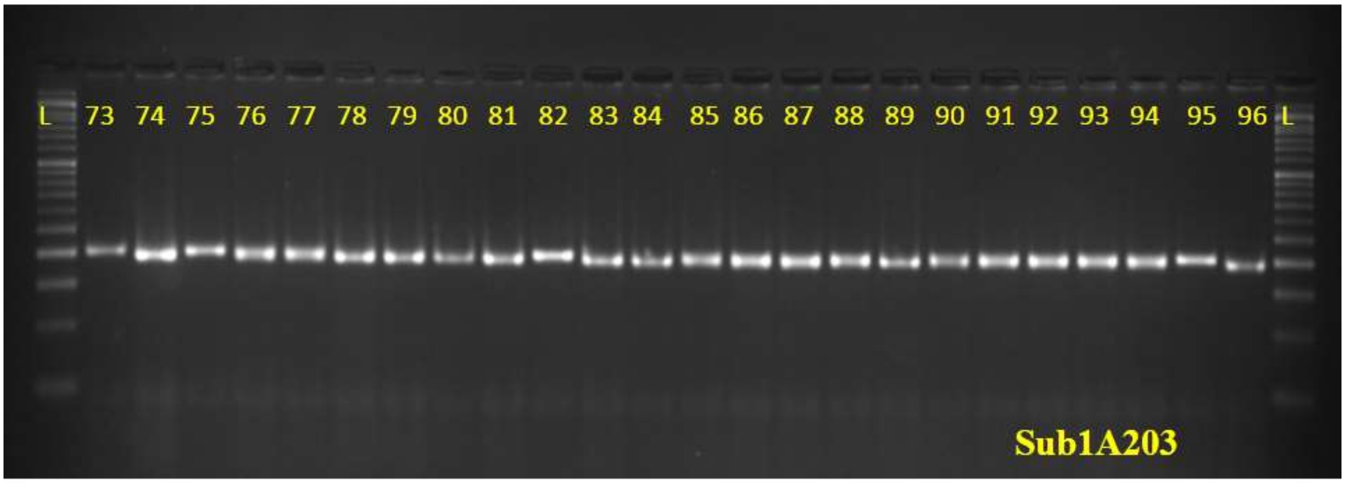
L 73 74 75 76 77 78 79 80 81 82 83 84 85 86 87 88 89 90 91 92 93 94 95 96 L
Sub1A203

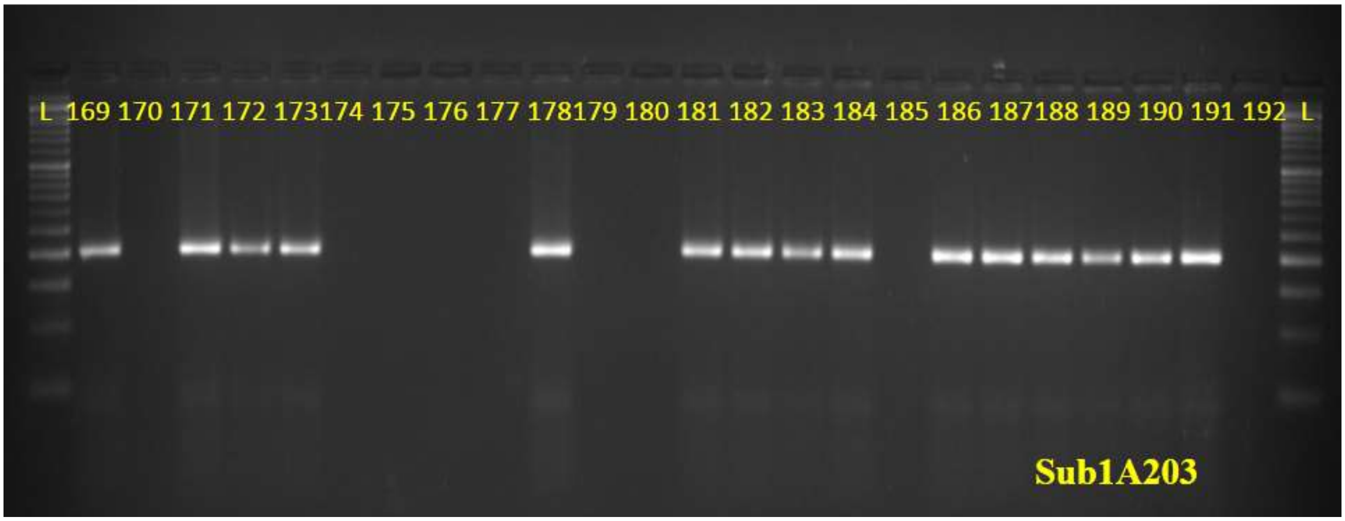
L 169 170 171 172 173174 175 176 177 178179 180 181 182 183 184 185 186 187188 189 190 191 192 L
Sub1A203

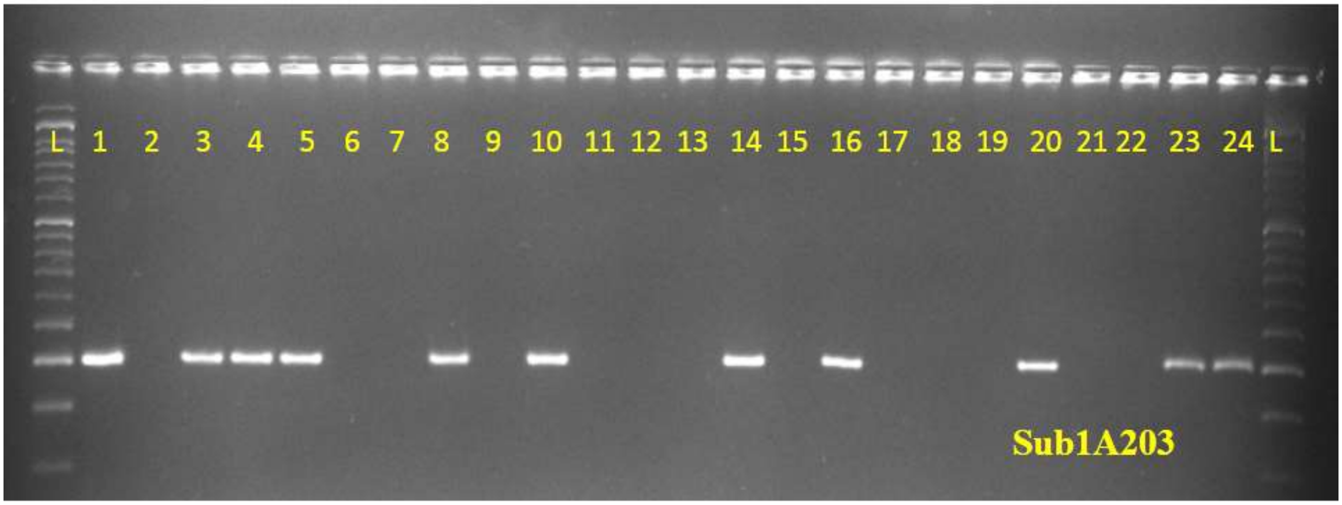
L 1 2 3 4 5 6 7 8 9 10 11 12 13 14 15 16 17 18 19 20 21 22 23 24 L
Sub1A203

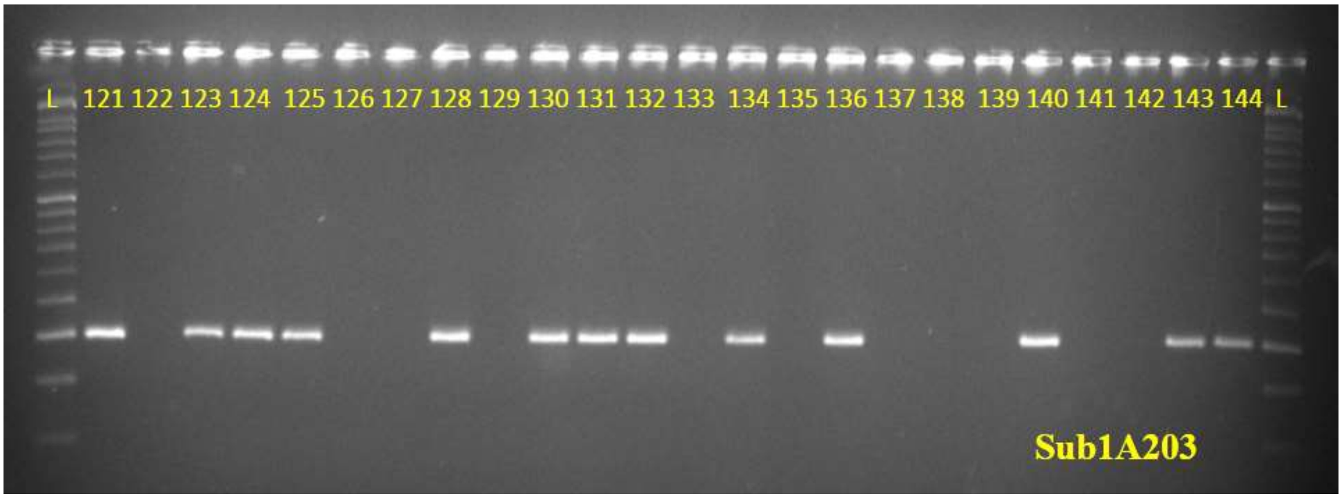
L 121 122 123 124 125 126 127 128 129 130 131 132 133 134 135 136 137 138 139 140 141 142 143 144 L
Sub1A203

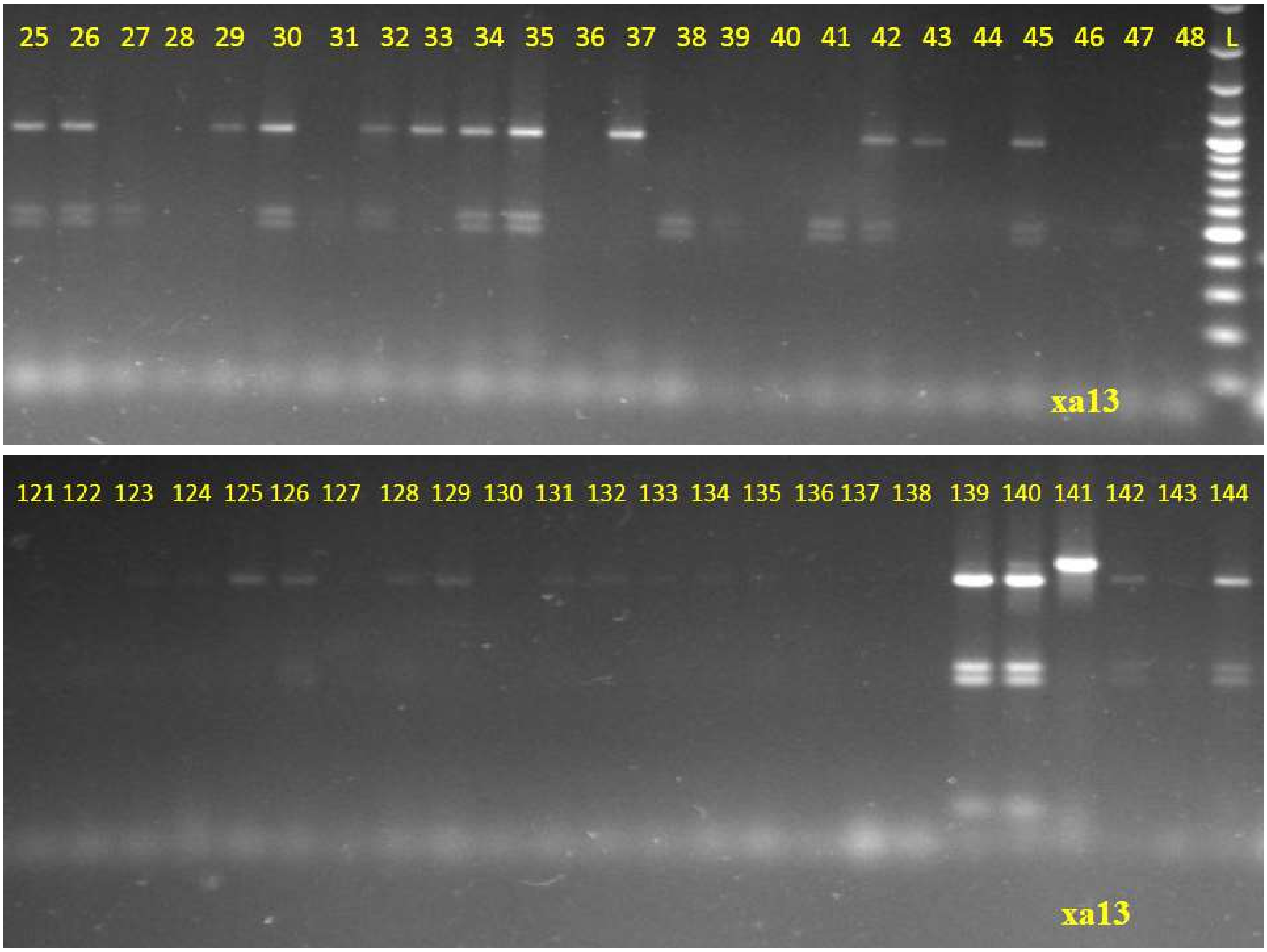
25 26 27 28 29 30 31 32 33 34 35 36 37 38 39 40 41 42 43 44 45 46 47 48 L
xa13
121 122 123 124 125 126 127 128 129 130 131 132 133 134 135 136 137 138 139 140 141 142 143 144
xa13

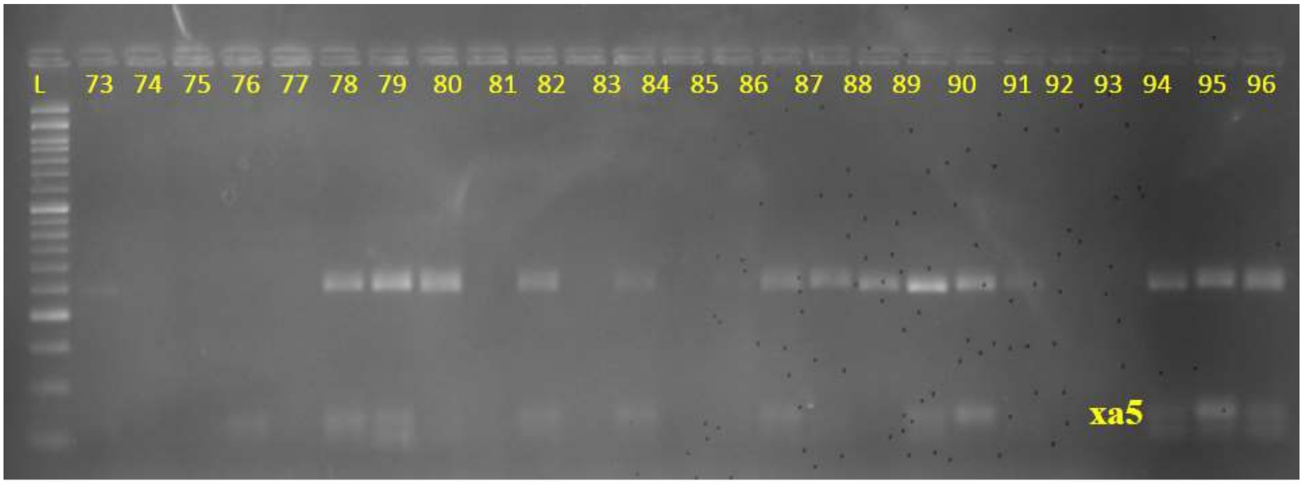
L 73 74 75 76 77 78 79 80 81 82 83 84 85 86 87 88 89 90 91 92 93 94 95 96
xa5

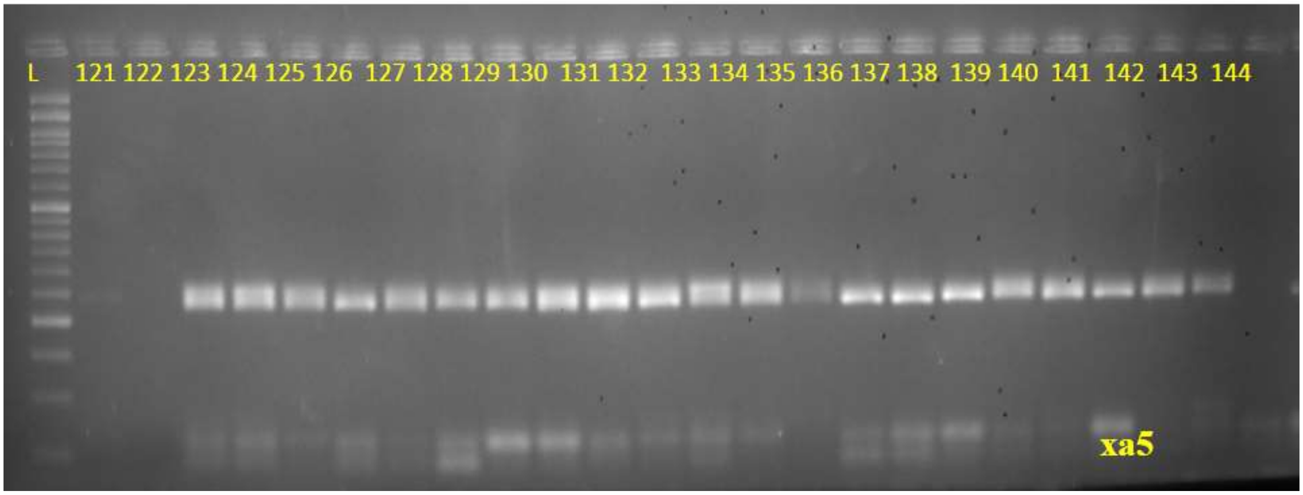
L 121 122 123 124 125 126 127 128 129 130 131 132 133 134 135 136 137 138 139 140 141 142 143 144
xa5

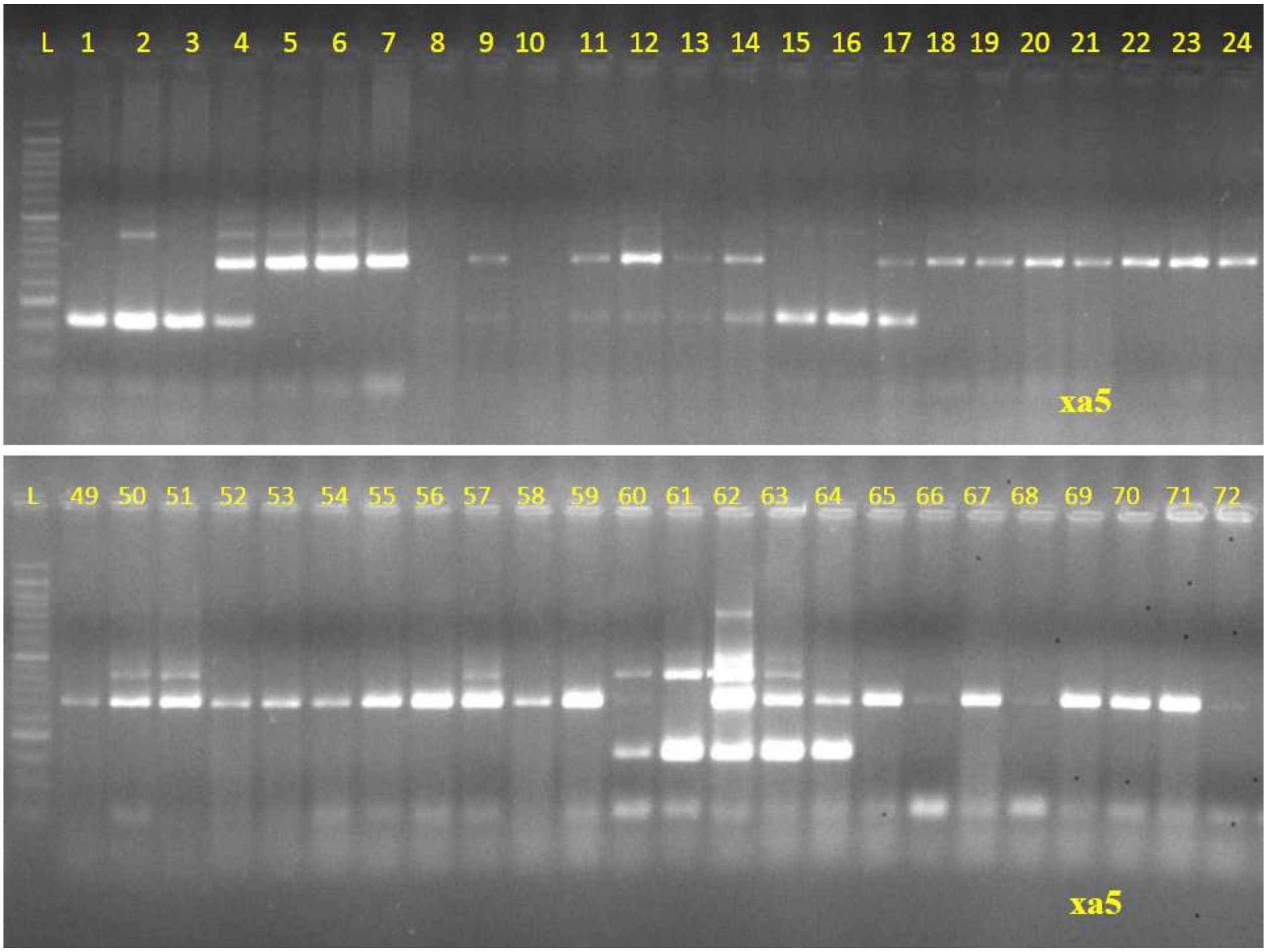
L 1 2 3 4 5 6 7 8 9 10 11 12 13 14 15 16 17 18 19 20 21 22 23 24
xa5
L 49 50 51 52 53 54 55 56 57 58 59 60 61 62 63 64 65 66 67 68 69 70 71 72
xa5

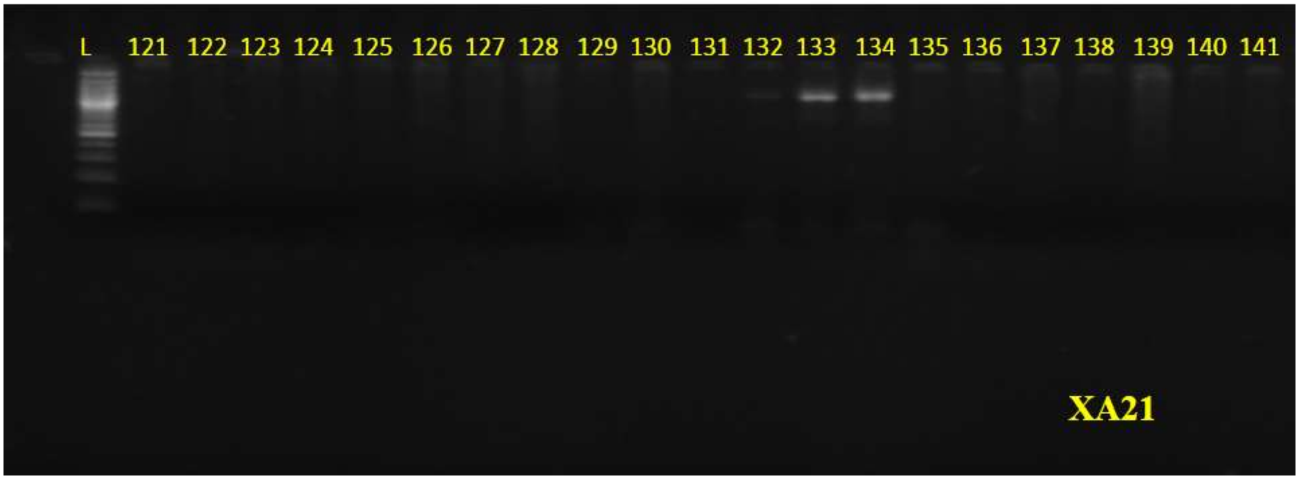
L
121
122
123
124
125
126
127
128
129
130
131
132
133
134
135
136
137
138
139
140
141
XA21

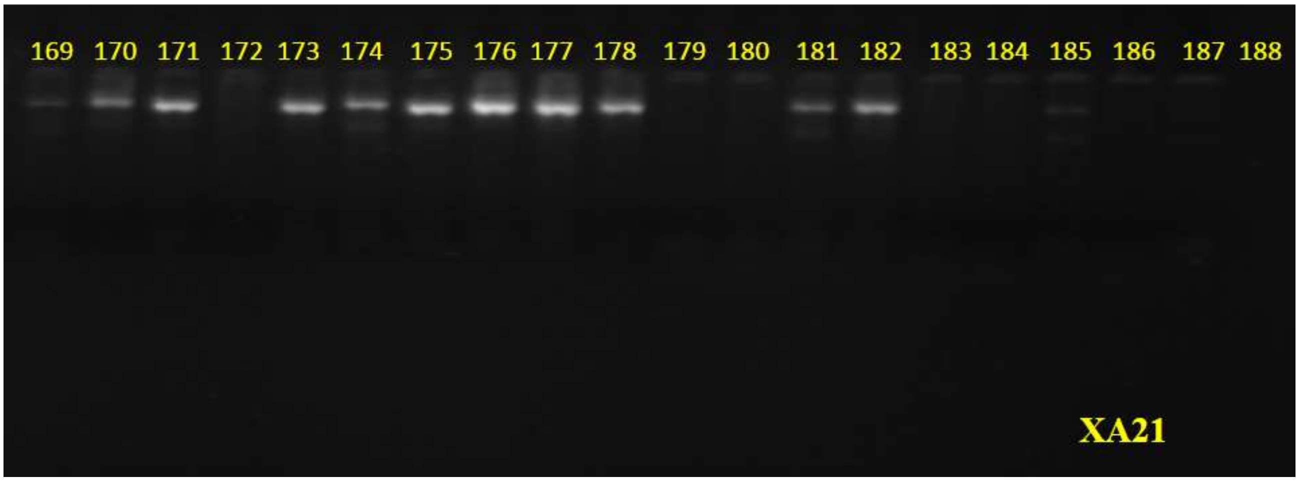
169
170
171
172
173
174
175
176
177
178
179
180
181
182
183
184
185
186
187
188
XA21

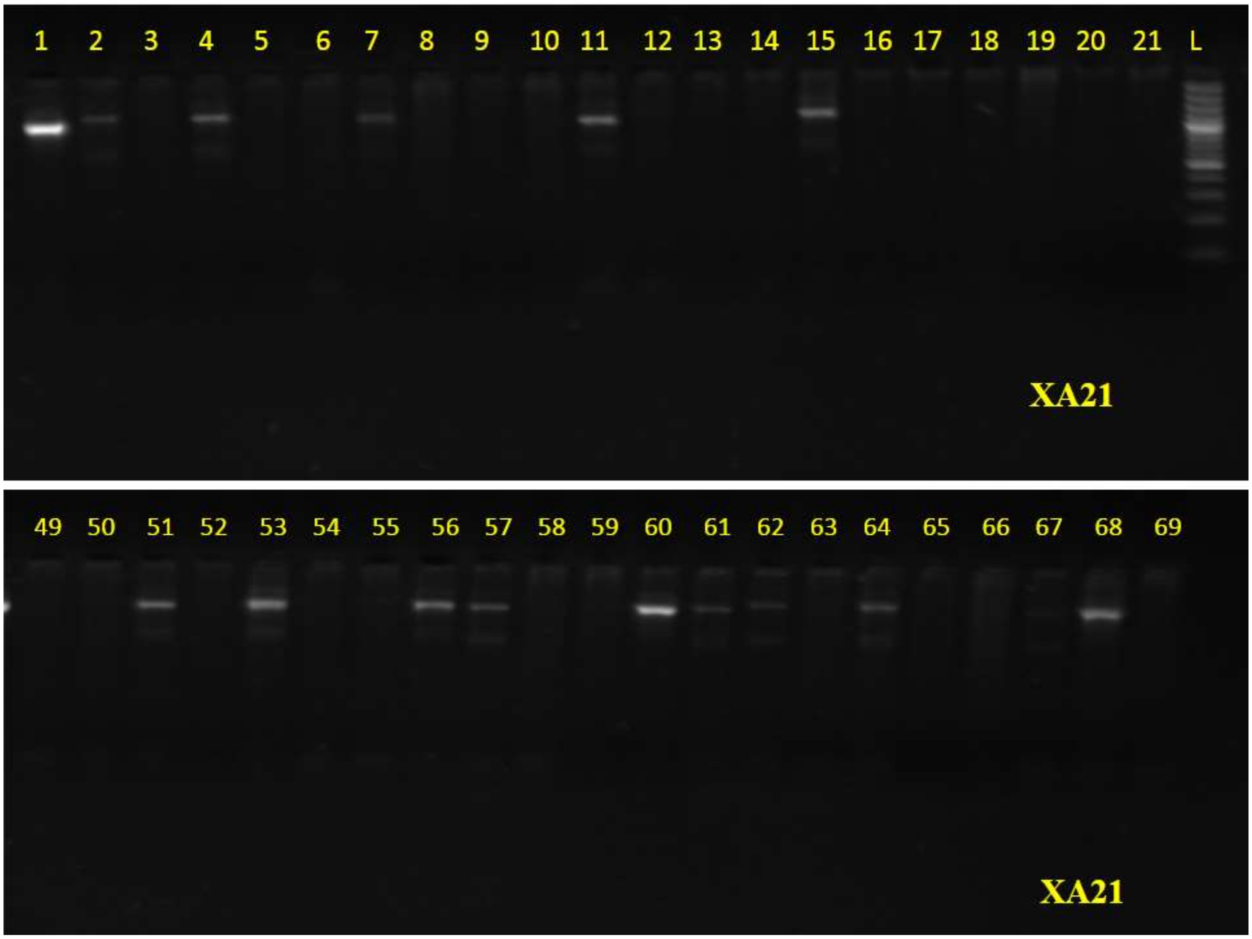
1 2 3 4 5 6 7 8 9 10 11 12 13 14 15 16 17 18 19 20 21 L
XA21
49 50 51 52 53 54 55 56 57 58 59 60 61 62 63 64 65 66 67 68 69
XA21

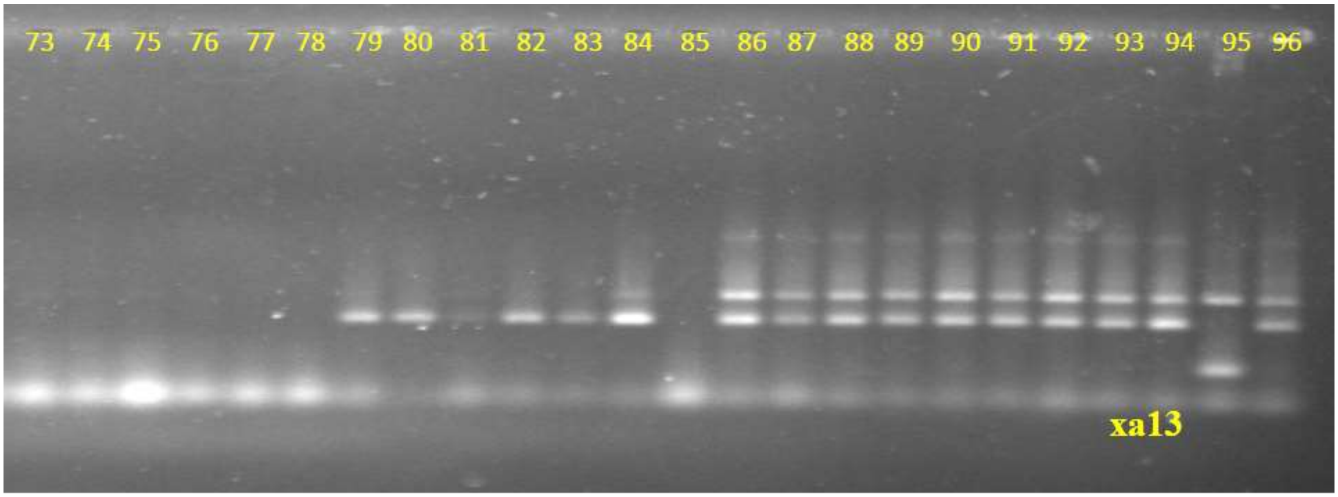
73 74 75 76 77 78 79 80 81 82 83 84 85 86 87 88 89 90 91 92 93 94 95 96
xa13

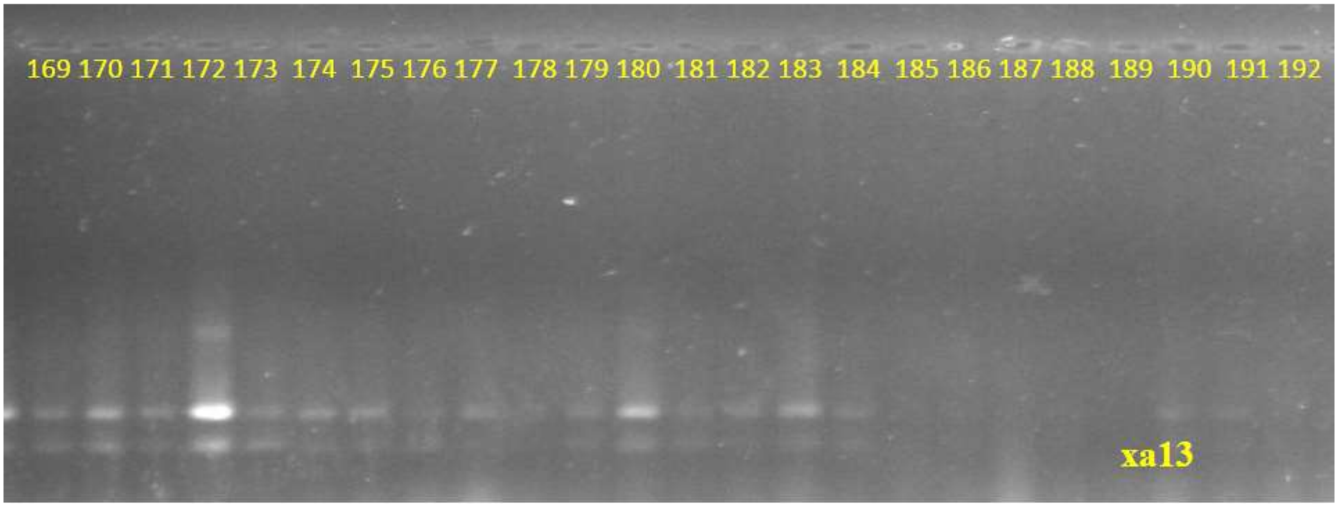
169 170 171 172 173 174 175 176 177 178 179 180 181 182 183 184 185 186 187 188 189 190 191 192
xa13

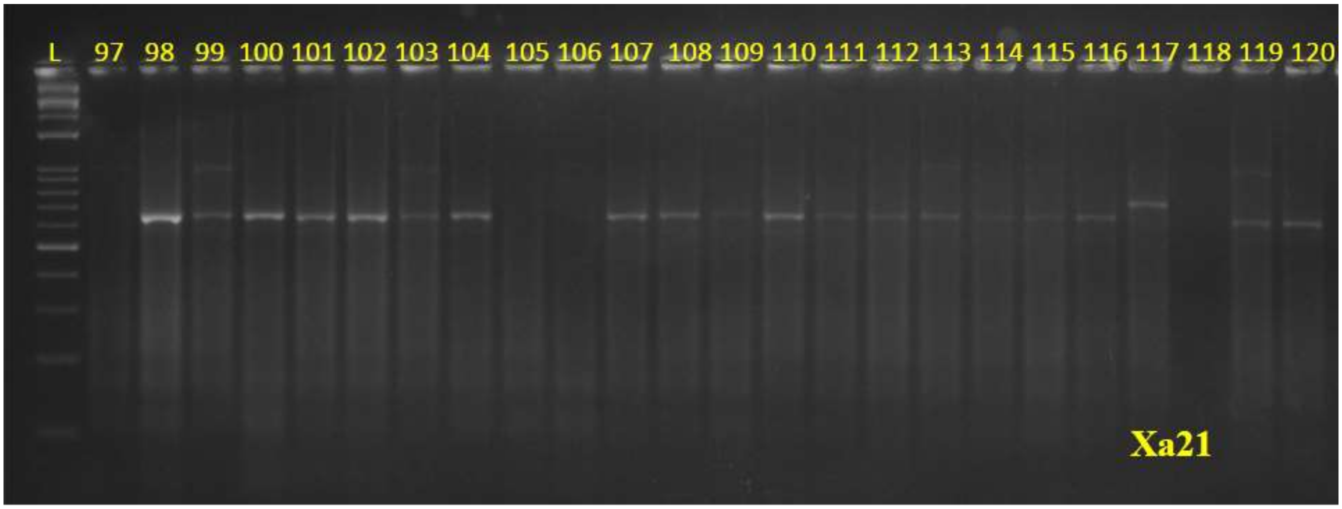
L 97 98 99 100 101 102 103 104 105 106 107 108 109 110 111 112 113 114 115 116 117 118 119 120
Xa21

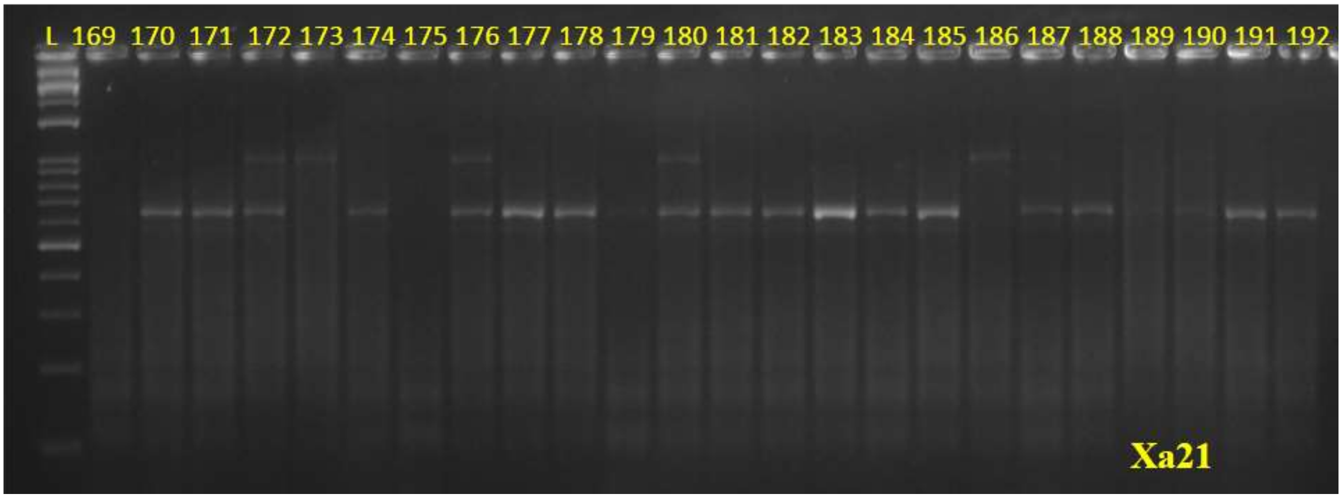
L 169 170 171 172 173 174 175 176 177 178 179 180 181 182 183 184 185 186 187 188 189 190 191 192
Xa21

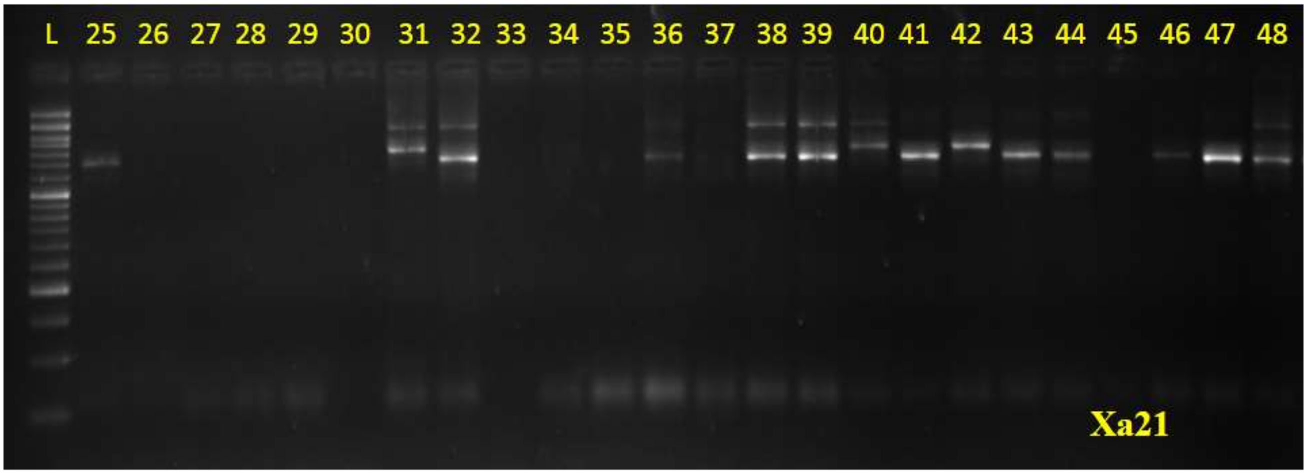
L 25 26 27 28 29 30 31 32 33 34 35 36 37 38 39 40 41 42 43 44 45 46 47 48
Xa21

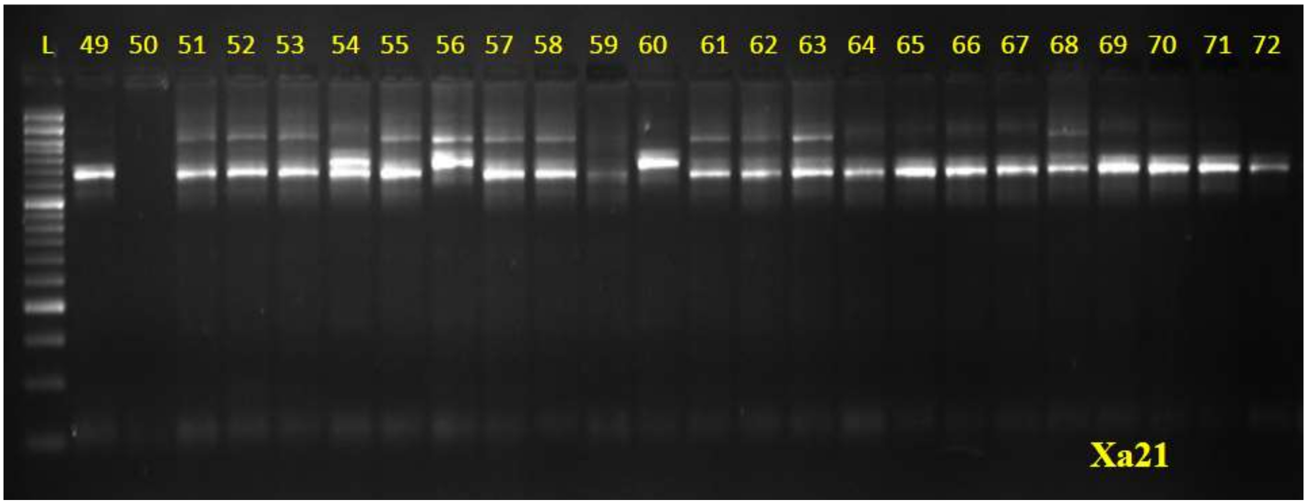
L 49 50 51 52 53 54 55 56 57 58 59 60 61 62 63 64 65 66 67 68 69 70 71 72
Xa21

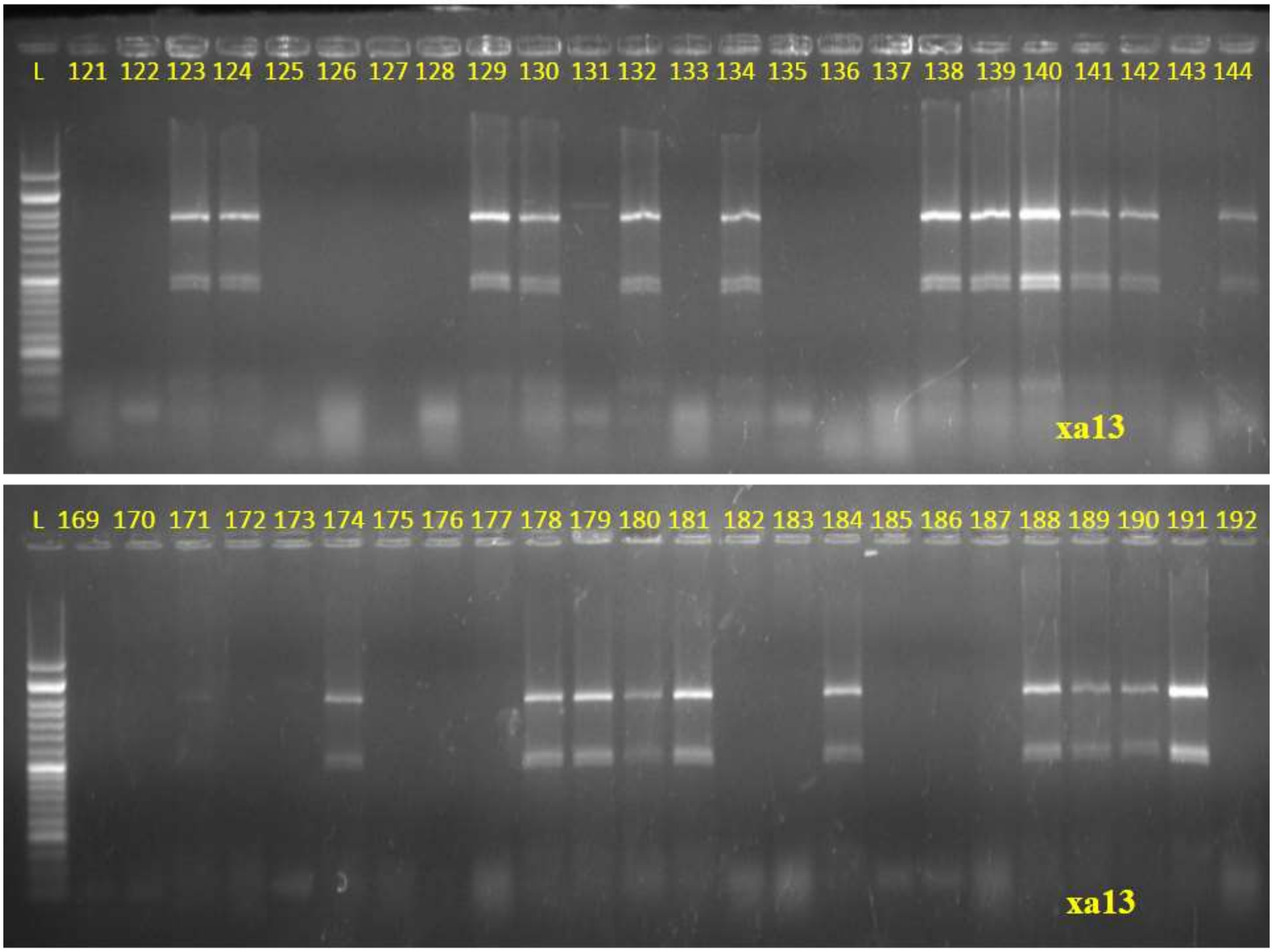
L 121 122 123 124 125 126 127 128 129 130 131 132 133 134 135 136 137 138 139 140 141 142 143 144
xa13
L 169 170 171 172 173 174 175 176 177 178 179 180 181 182 183 184 185 186 187 188 189 190 191 192
xa13

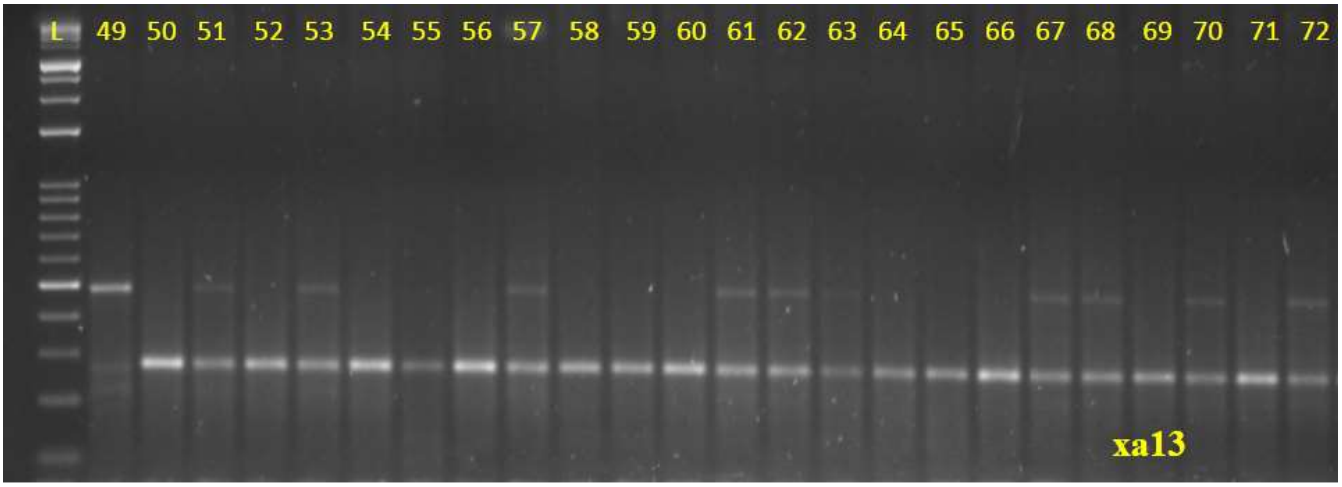
L 49 50 51 52 53 54 55 56 57 58 59 60 61 62 63 64 65 66 67 68 69 70 71 72
xa13
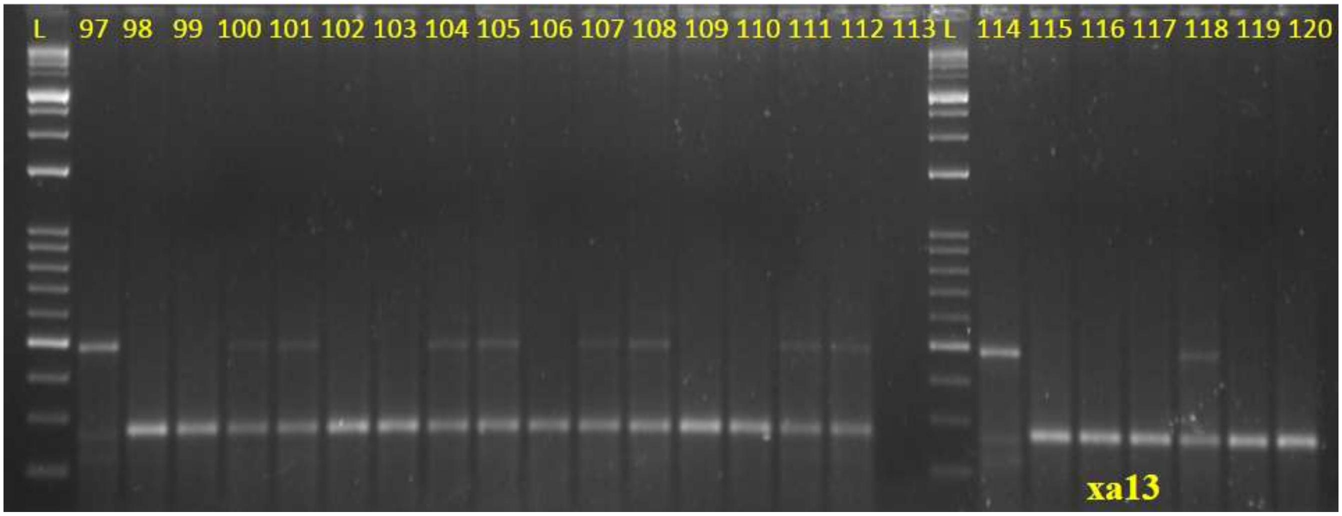
L 97 98 99 100 101 102 103 104 105 106 107 108 109 110 111 112 113 L 114 115 116 117 118 119 120
xa13

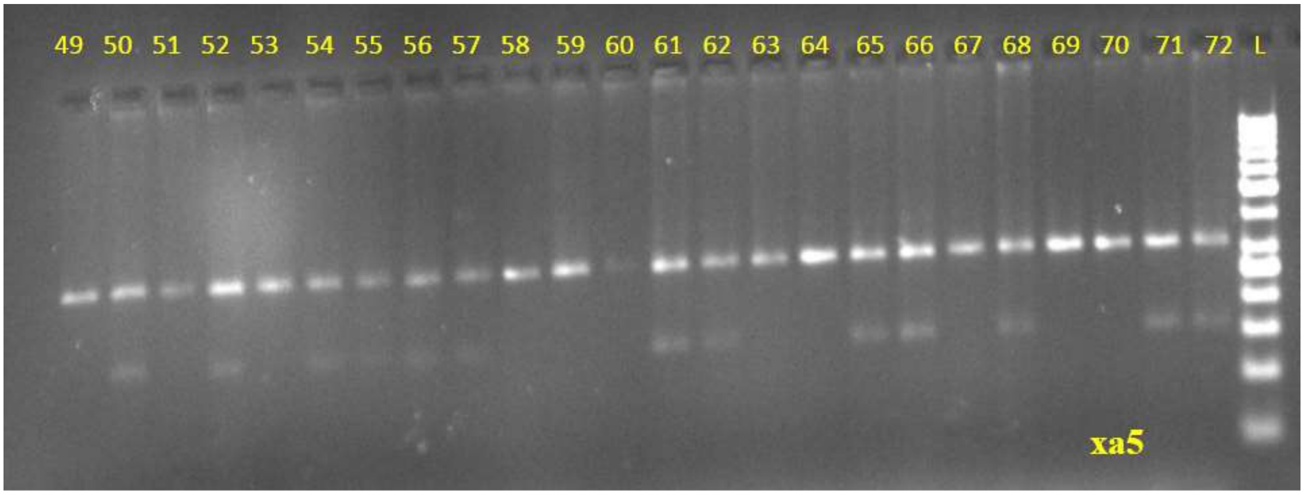
49 50 51 52 53 54 55 56 57 58 59 60 61 62 63 64 65 66 67 68 69 70 71 72 L
xa5

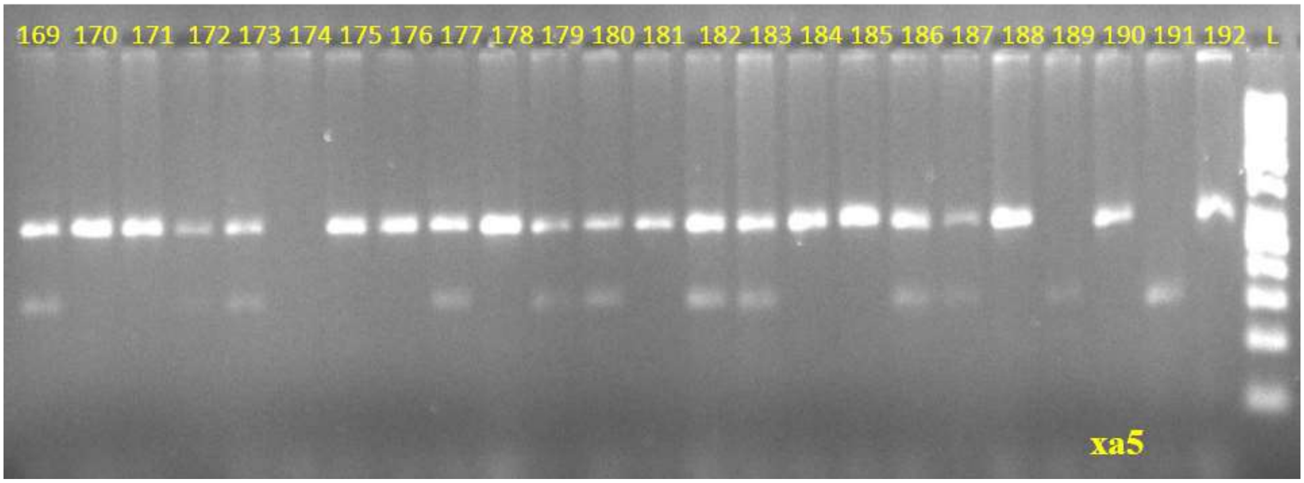
169 170 171 172 173 174 175 176 177 178 179 180 181 182 183 184 185 186 187 188 189 190 191 192 L
xa5

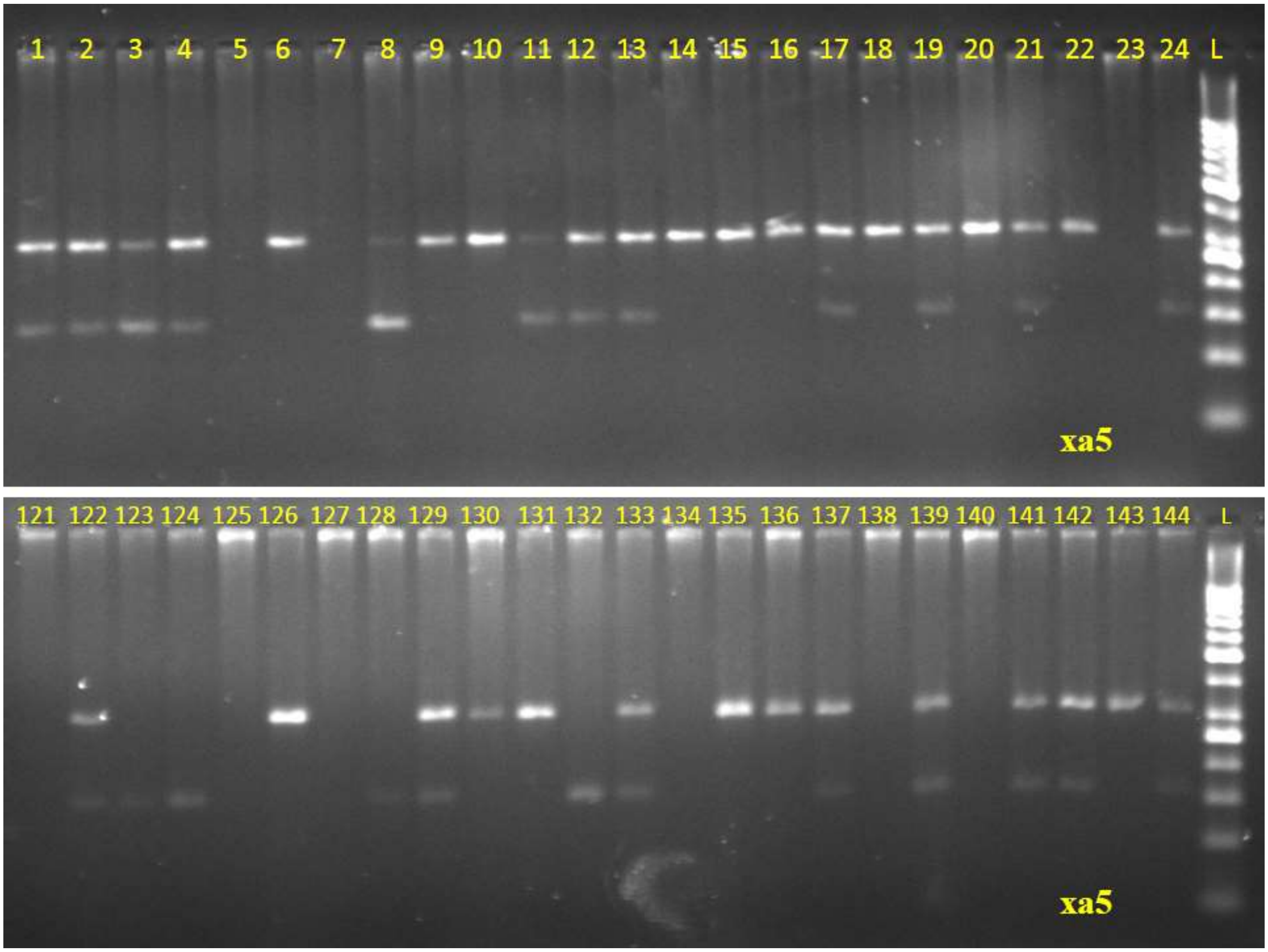
1 2 3 4 5 6 7 8 9 10 11 12 13 14 15 16 17 18 19 20 21 22 23 24 L
xa5
121 122 123 124 125 126 127 128 129 130 131 132 133 134 135 136 137 138 139 140 141 142 143 144 L
xa5

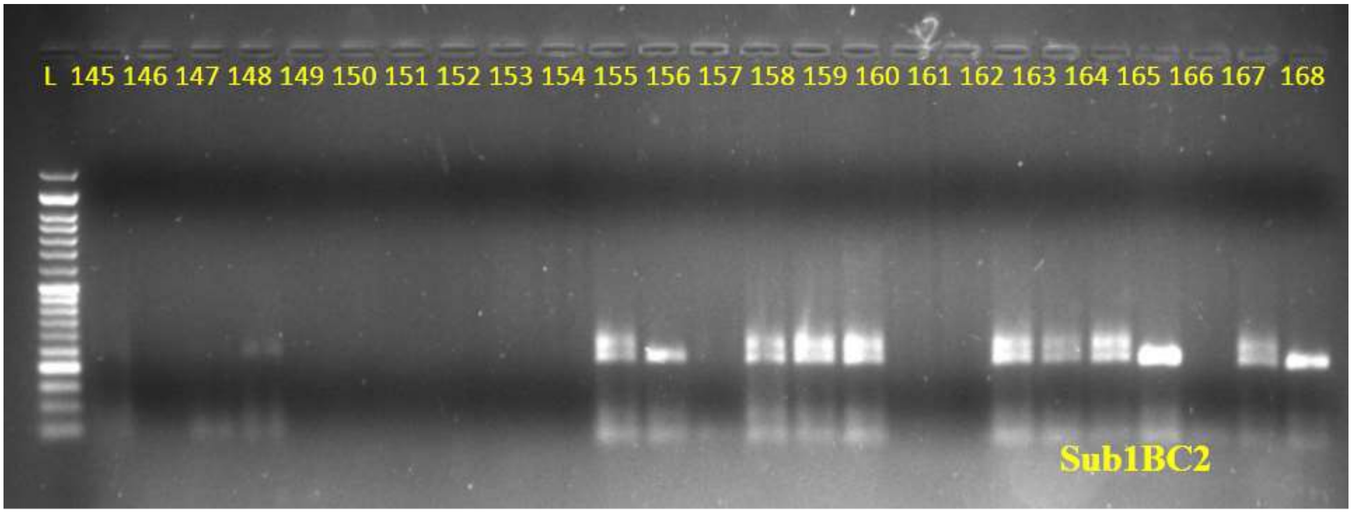
L 145 146 147 148 149 150 151 152 153 154 155 156 157 158 159 160 161 162 163 164 165 166 167 168
Sub1BC2

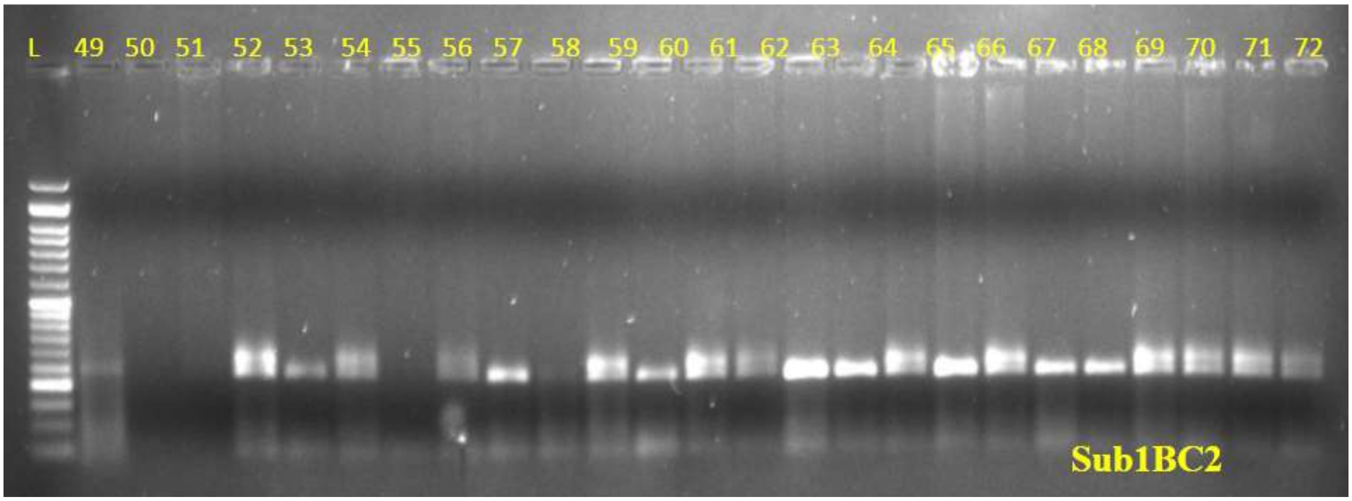
L 49 50 51 52 53 54 55 56 57 58 59 60 61 62 63 64 65 66 67 68 69 70 71 72
Sub1BC2

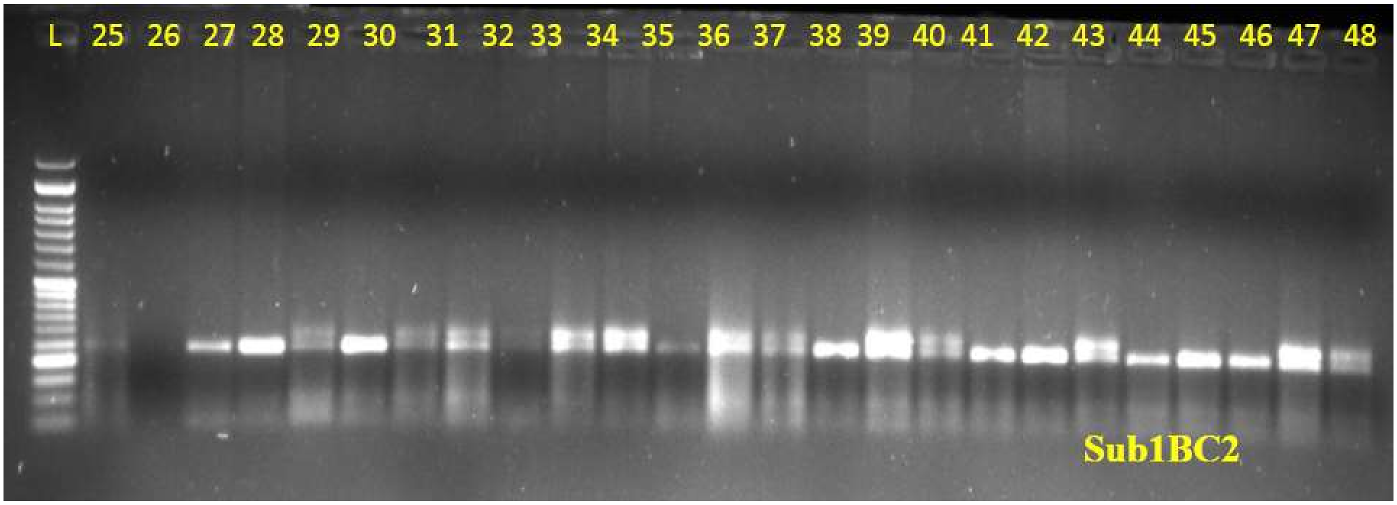
L 25 26 27 28 29 30 31 32 33 34 35 36 37 38 39 40 41 42 43 44 45 46 47 48
Sub1BC2

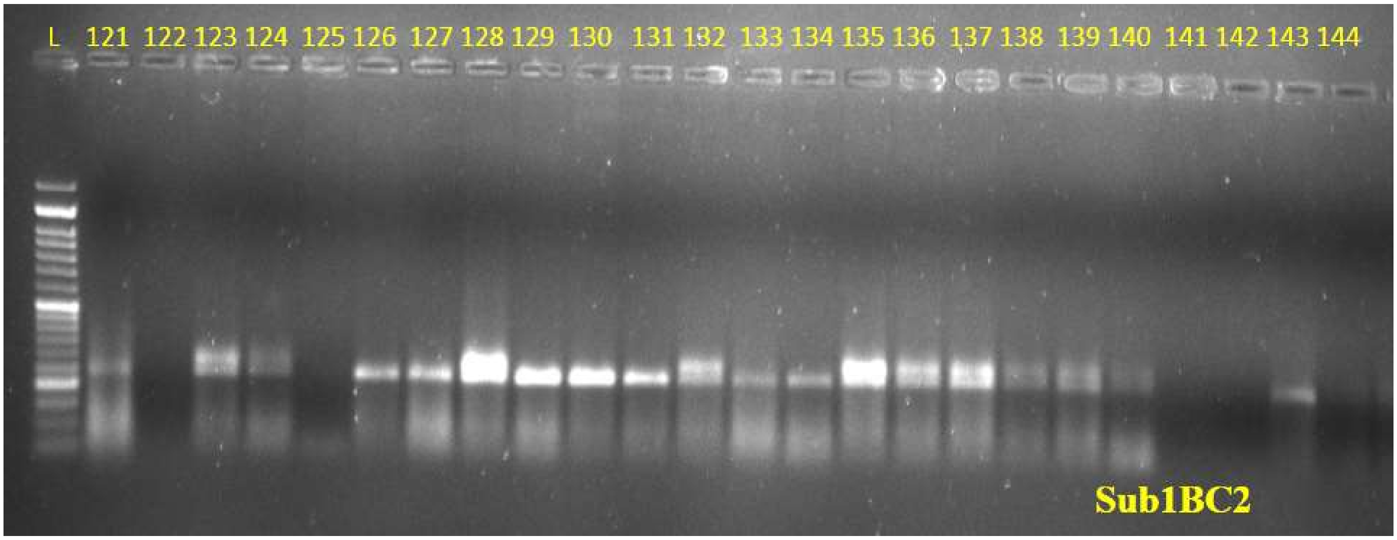
L 121 122 123 124 125 126 127 128 129 130 131 132 133 134 135 136 137 138 139 140 141 142 143 144
Sub1BC2

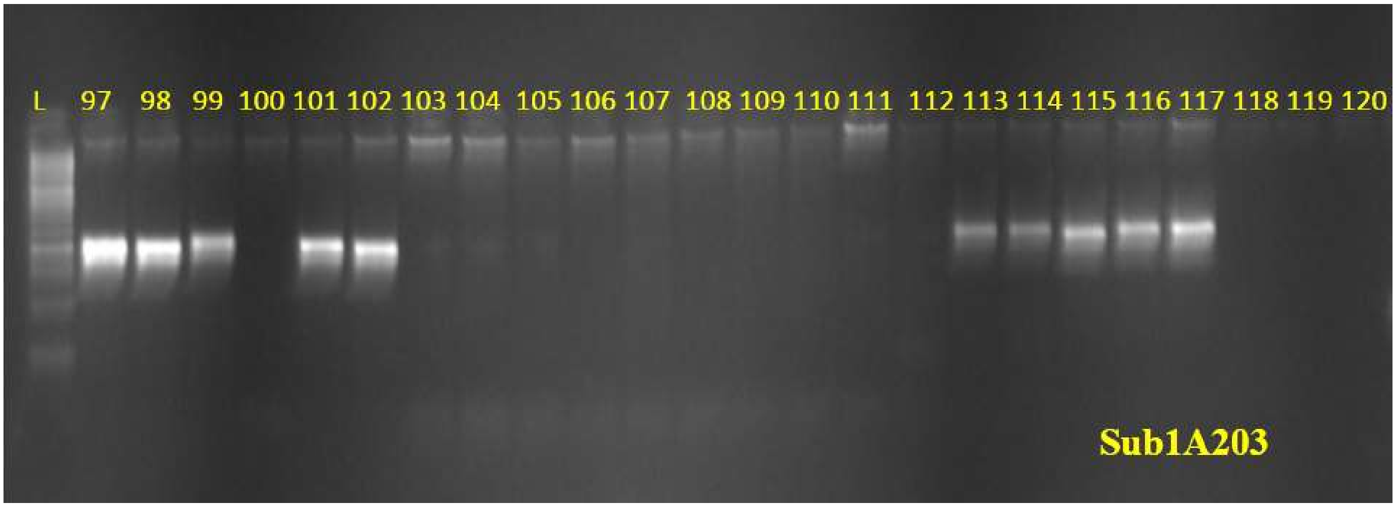
L 97 98 99 100 101 102 103 104 105 106 107 108 109 110 111 112 113 114 115 116 117 118 119 120
Sub1A203

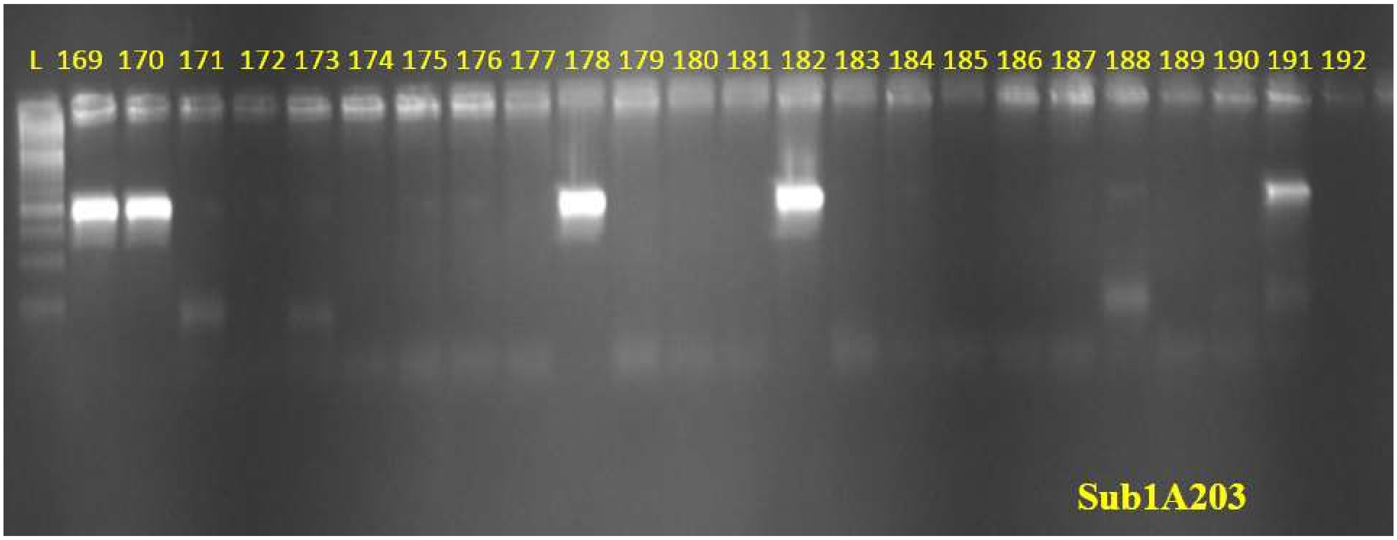
L 169 170 171 172 173 174 175 176 177 178 179 180 181 182 183 184 185 186 187 188 189 190 191 192
Sub1A203

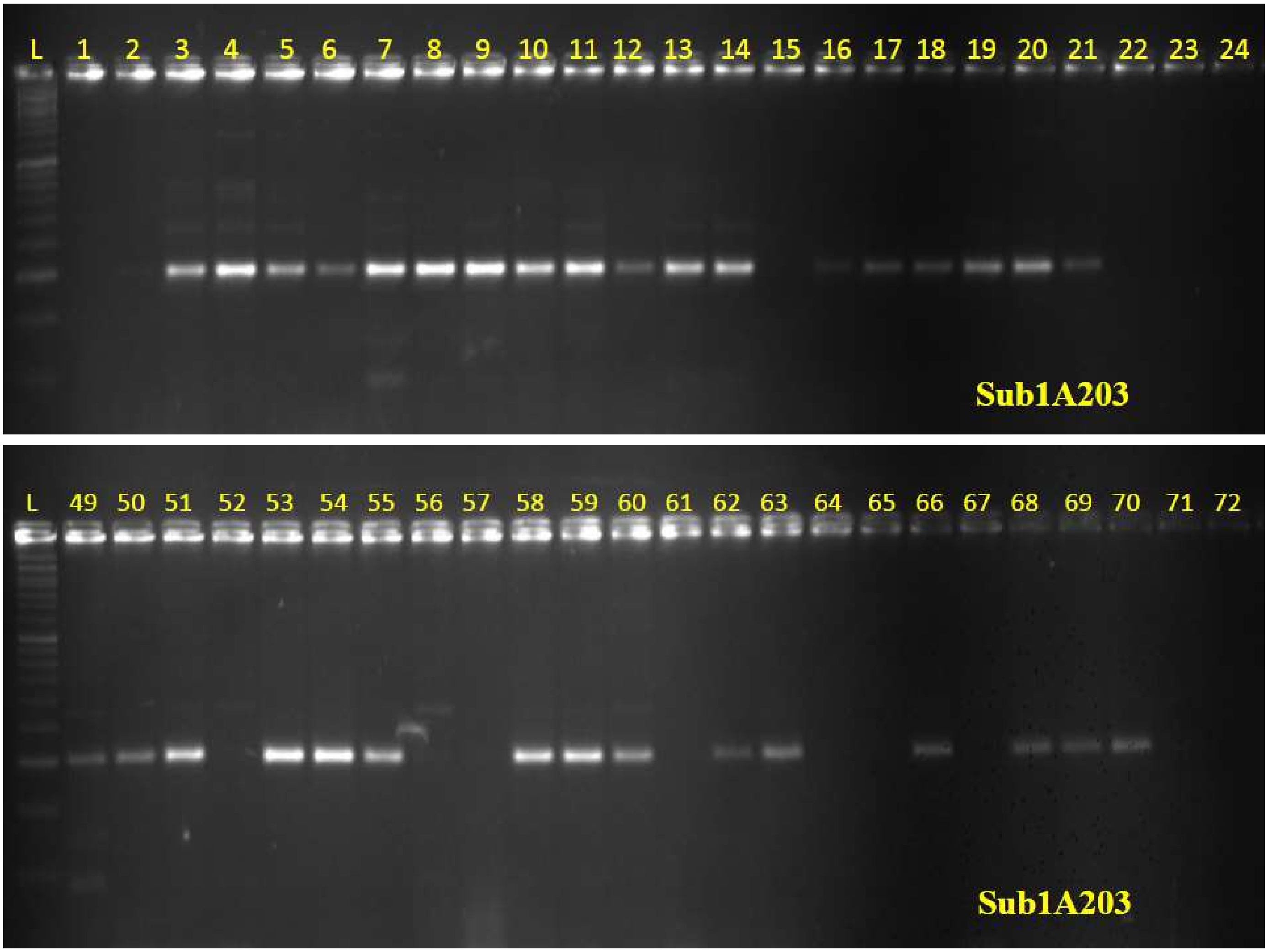
L 1 2 3 4 5 6 7 8 9 10 11 12 13 14 15 16 17 18 19 20 21 22 23 24
Sub1A203
L 49 50 51 52 53 54 55 56 57 58 59 60 61 62 63 64 65 66 67 68 69 70 71 72
Sub1A203

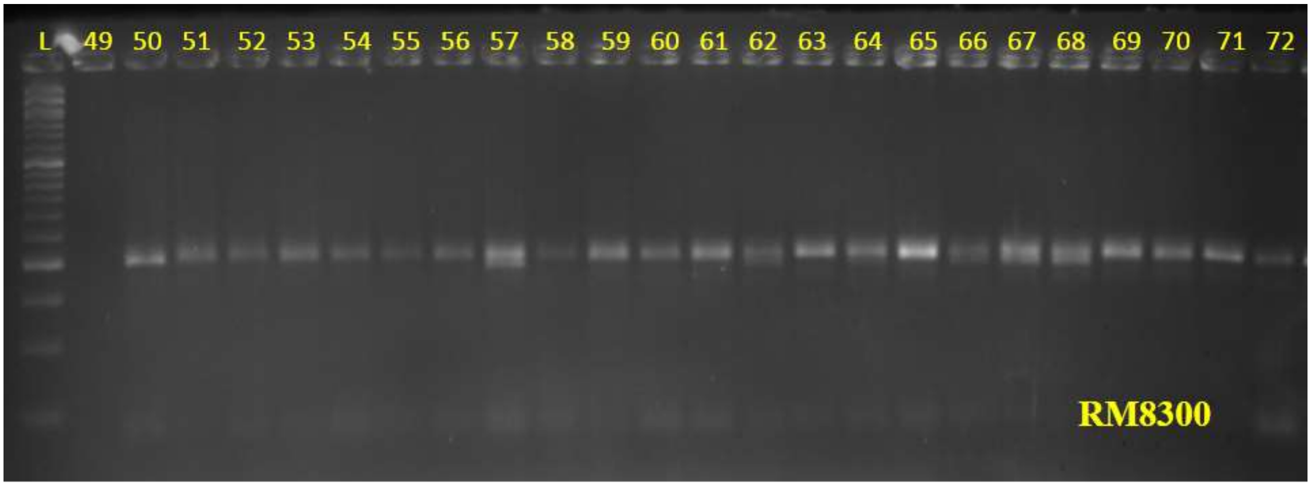
L 49 50 51 52 53 54 55 56 57 58 59 60 61 62 63 64 65 66 67 68 69 70 71 72
RM8300

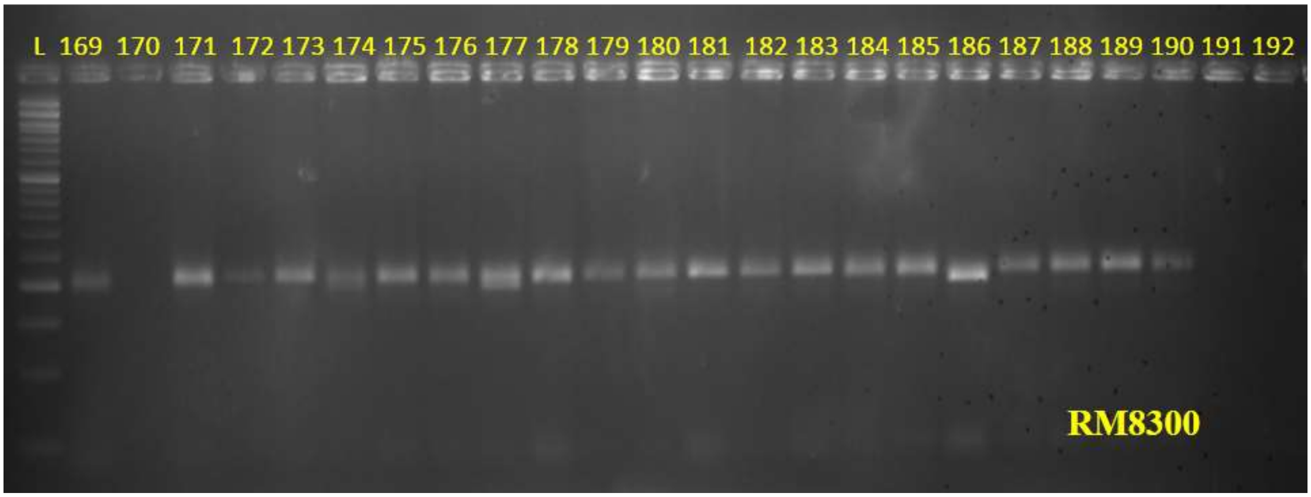
L 169 170 171 172 173 174 175 176 177 178 179 180 181 182 183 184 185 186 187 188 189 190 191 192
RM8300

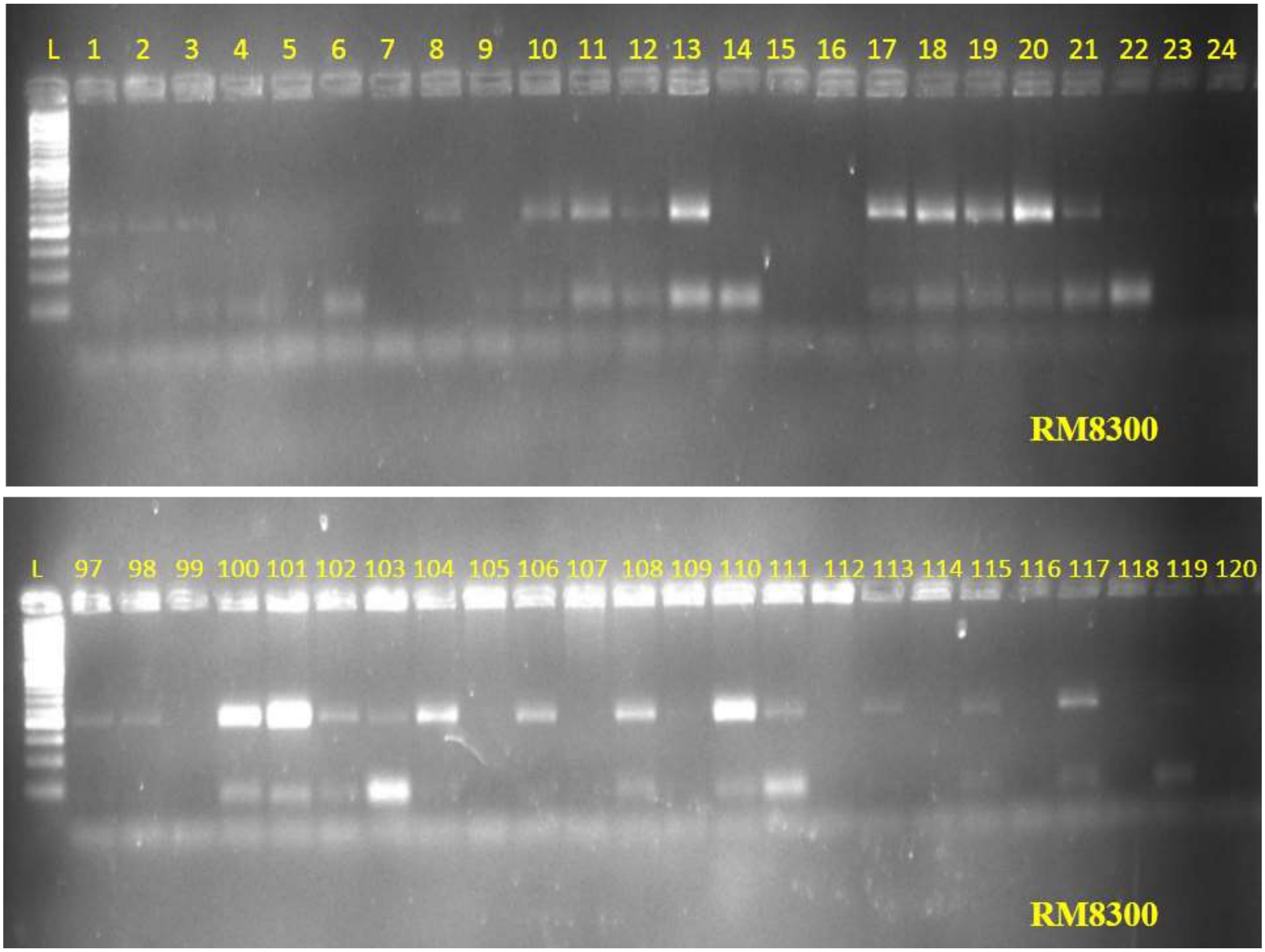

Supplementary figure S6. Molecular screening of BC3F1 progenies in rice variety, Ranidhan background for submergence tolerance and bacterial blight resistance

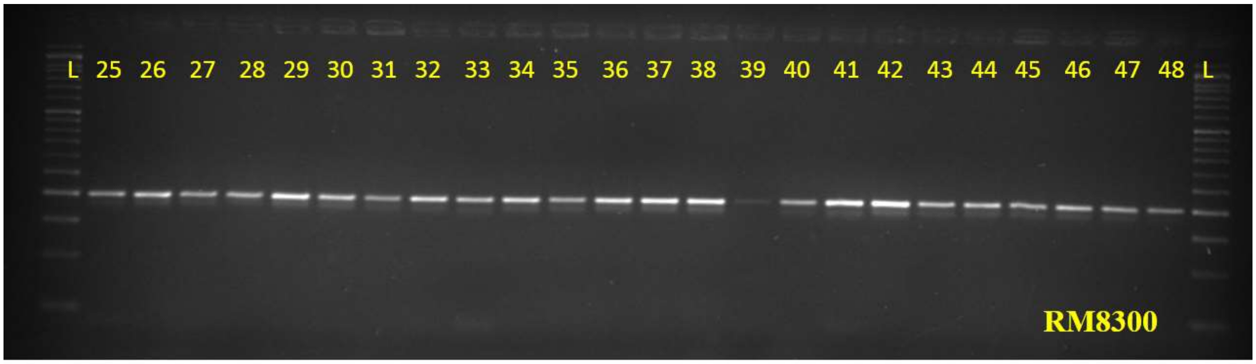
L 25 26 27 28 29 30 31 32 33 34 35 36 37 38 39 40 41 42 43 44 45 46 47 48 L
RM8300

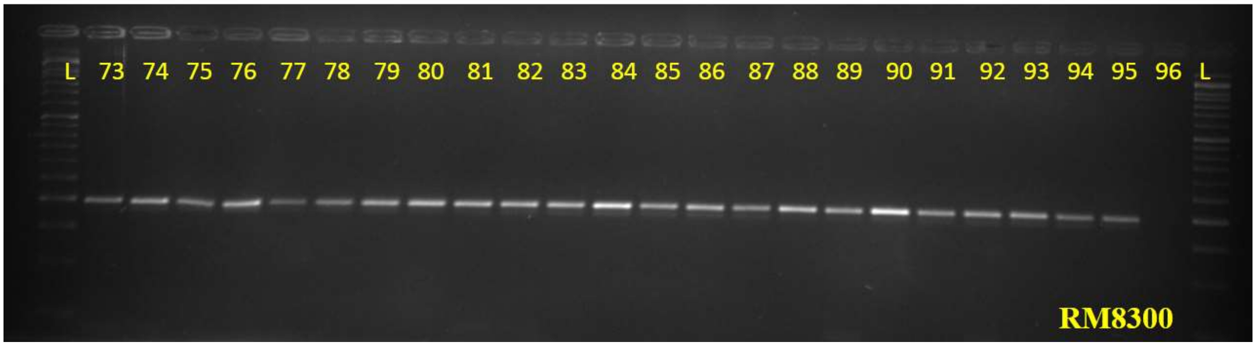
L 73 74 75 76 77 78 79 80 81 82 83 84 85 86 87 88 89 90 91 92 93 94 95 96 L
RM8300

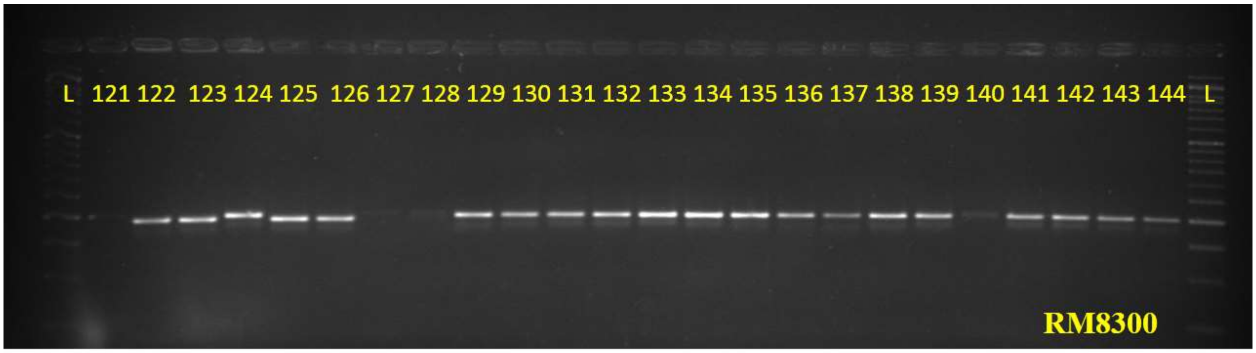
L 121 122 123 124 125 126 127 128 129 130 131 132 133 134 135 136 137 138 139 140 141 142 143 144 L
RM8300

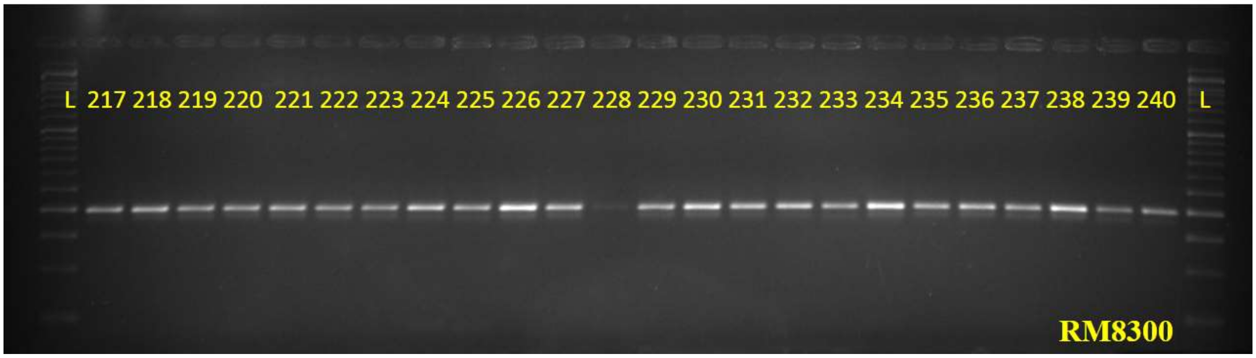
L 217 218 219 220 221 222 223 224 225 226 227 228 229 230 231 232 233 234 235 236 237 238 239 240 L
RM8300

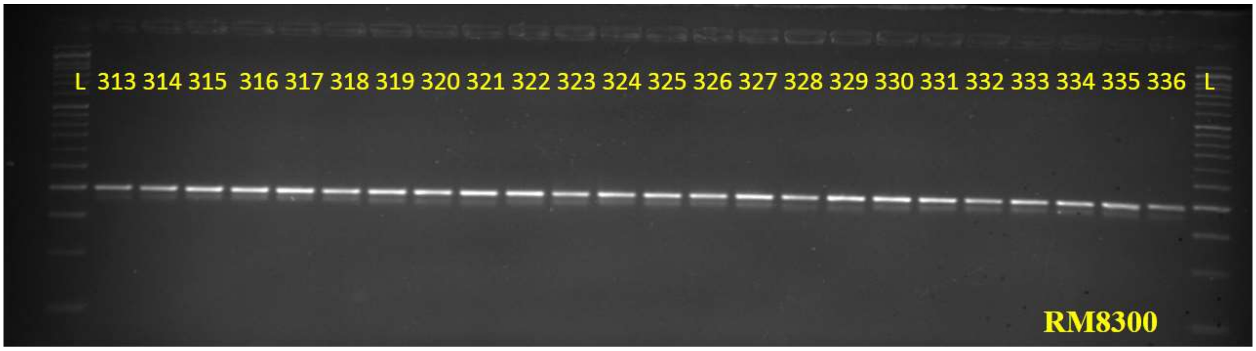
L 313 314 315 316 317 318 319 320 321 322 323 324 325 326 327 328 329 330 331 332 333 334 335 336 L
RM8300

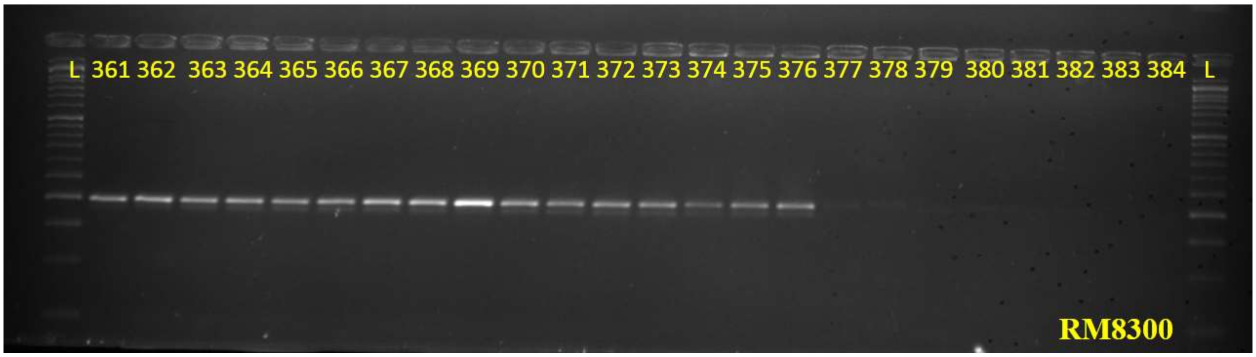
L 361 362 363 364 365 366 367 368 369 370 371 372 373 374 375 376 377 378 379 380 381 382 383 384 L
RM8300

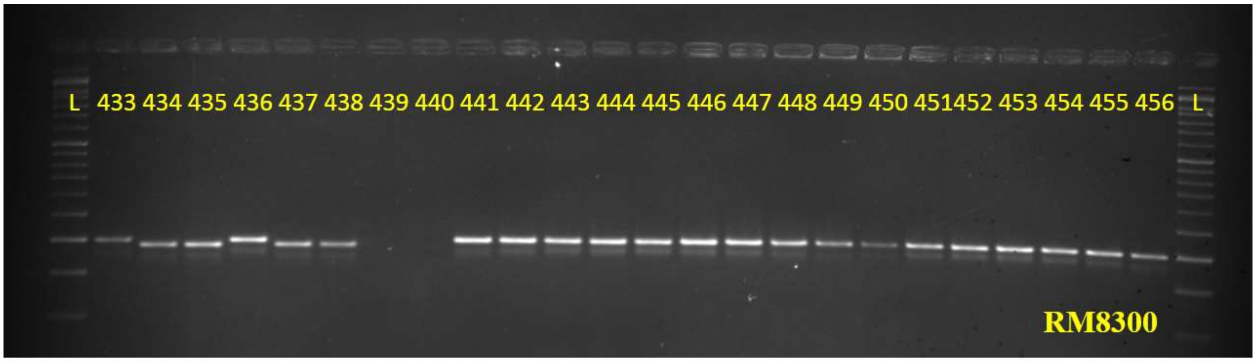
L 433 434 435 436 437 438 439 440 441 442 443 444 445 446 447 448 449 450 451452 453 454 455 456 L
RM8300

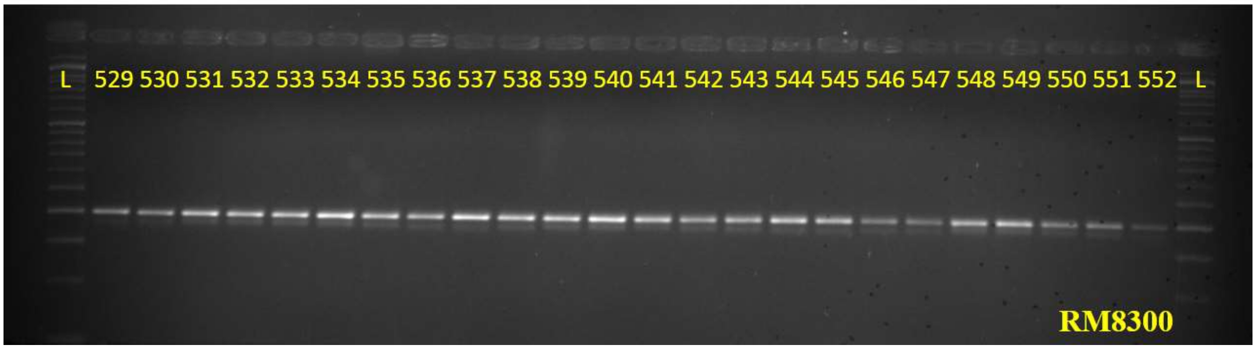
L 529 530 531 532 533 534 535 536 537 538 539 540 541 542 543 544 545 546 547 548 549 550 551 552 L
RM8300

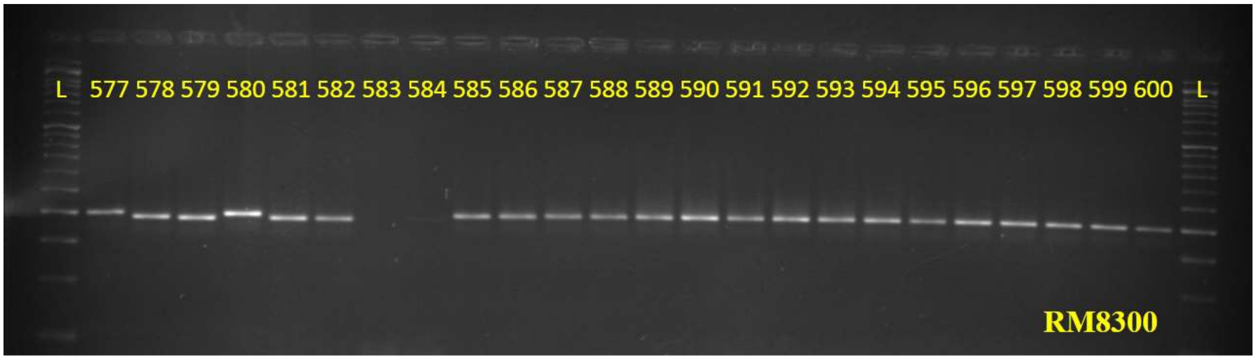
L 577 578 579 580 581 582 583 584 585 586 587 588 589 590 591 592 593 594 595 596 597 598 599 600 L
RM8300

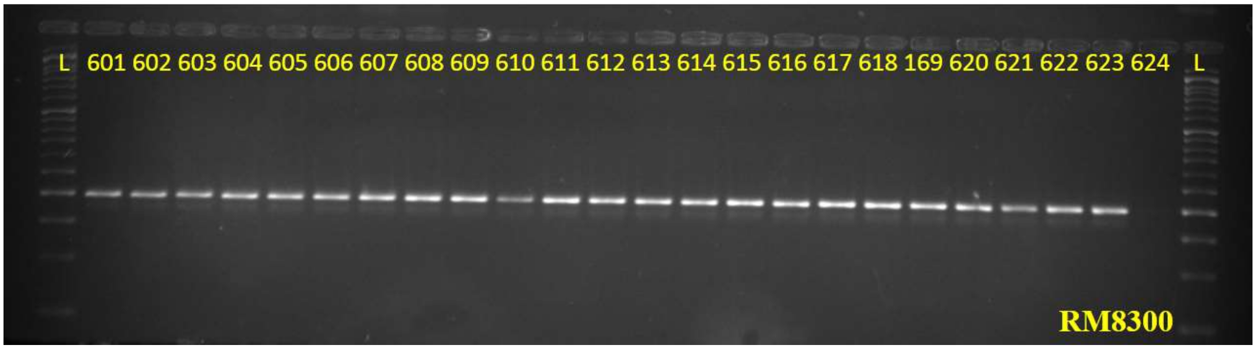
L 601 602 603 604 605 606 607 608 609 610 611 612 613 614 615 616 617 618 169 620 621 622 623 624 L
RM8300

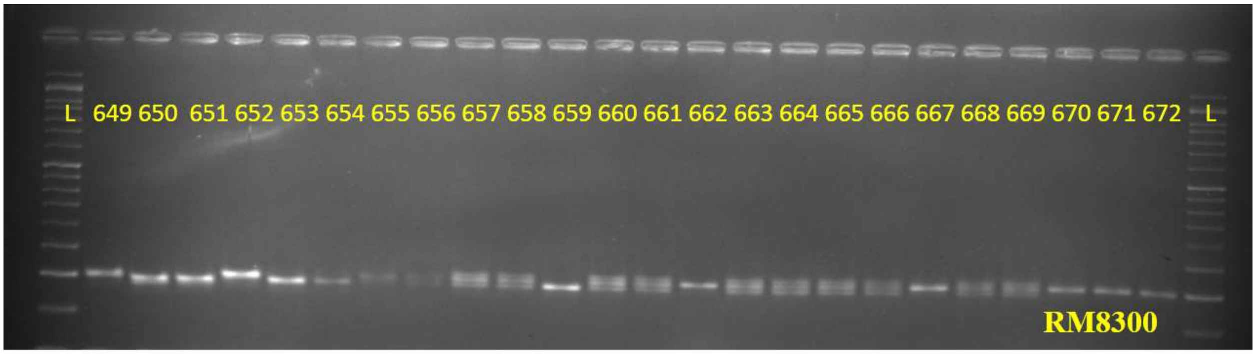
L 649 650 651 652 653 654 655 656 657 658 659 660 661 662 663 664 665 666 667 668 669 670 671 672 L
RM8300

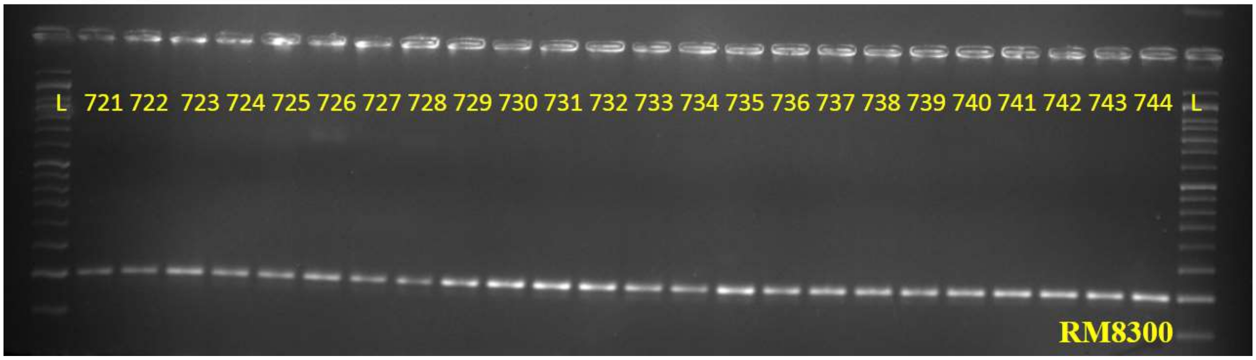
L 721 722 723 724 725 726 727 728 729 730 731 732 733 734 735 736 737 738 739 740 741 742 743 744 L
RM8300

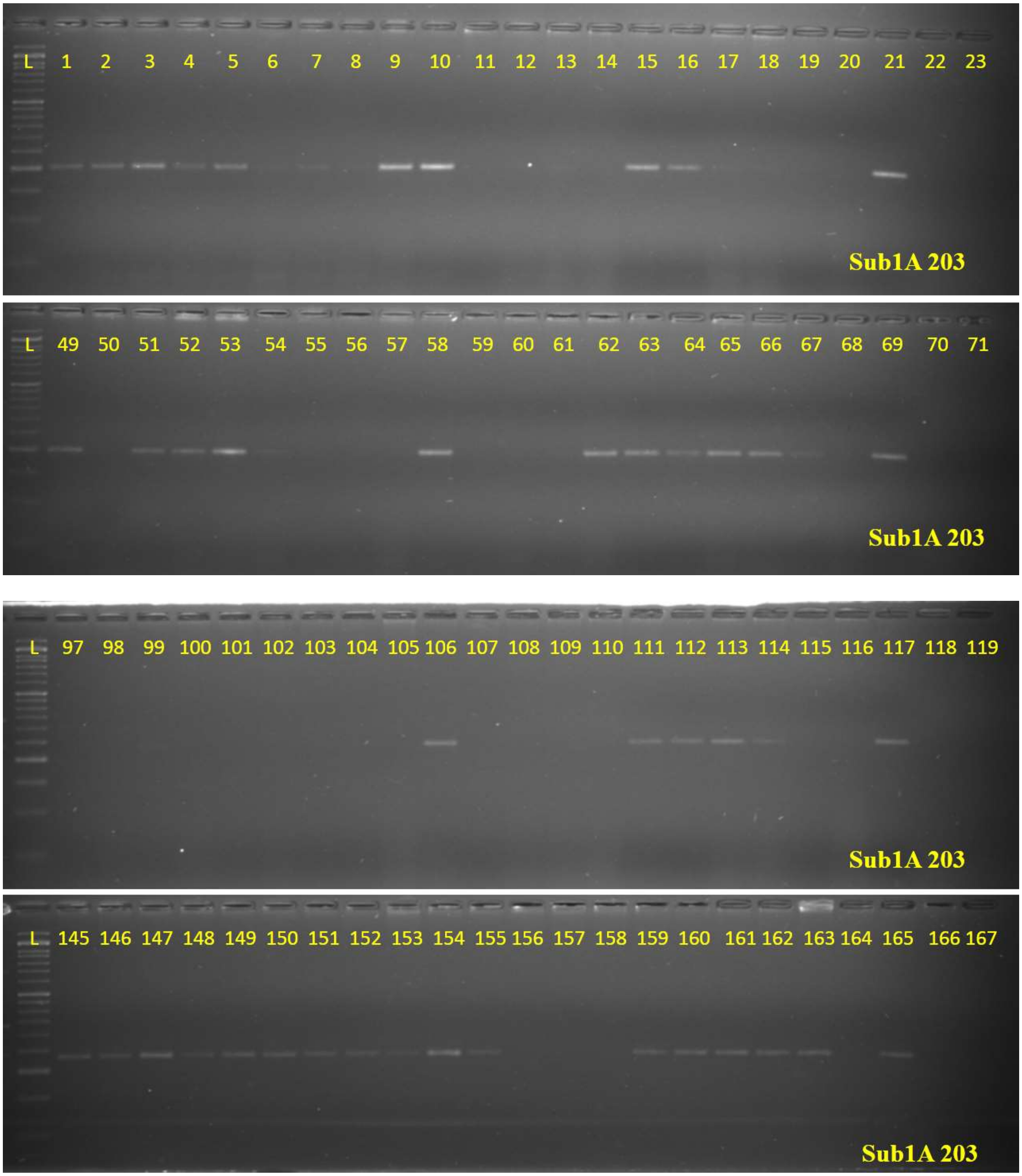
L 1 2 3 4 5 6 7 8 9 10 11 12 13 14 15 16 17 18 19 20 21 22 23
Sub1A 203
L 49 50 51 52 53 54 55 56 57 58 59 60 61 62 63 64 65 66 67 68 69 70 71
Sub1A 203
L 97 98 99 100 101 102 103 104 105 106 107 108 109 110 111 112 113 114 115 116 117 118 119
Sub1A 203
L 145 146 147 148 149 150 151 152 153 154 155 156 157 158 159 160 161 162 163 164 165 166 167
Sub1A 203

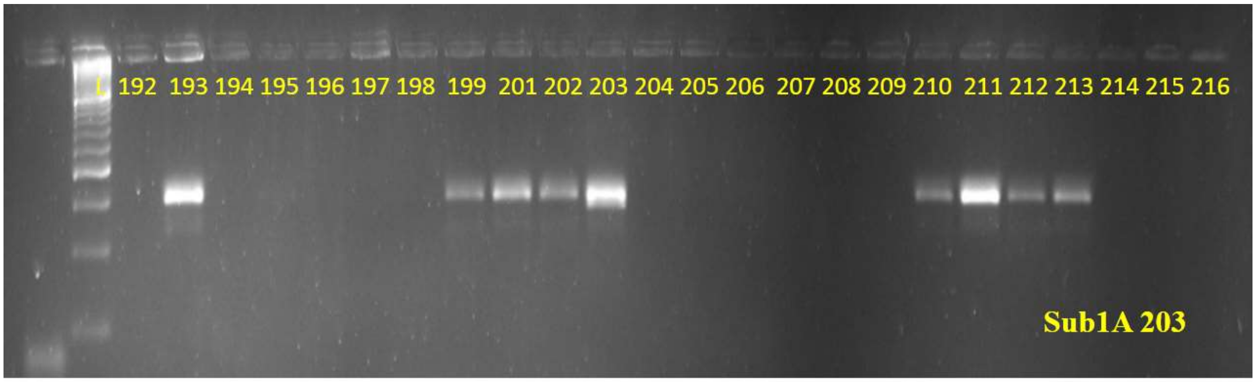
L 192 193 194 195 196 197 198 199 201 202 203 204 205 206 207 208 209 210 211 212 213 214 215 216
Sub1A 203

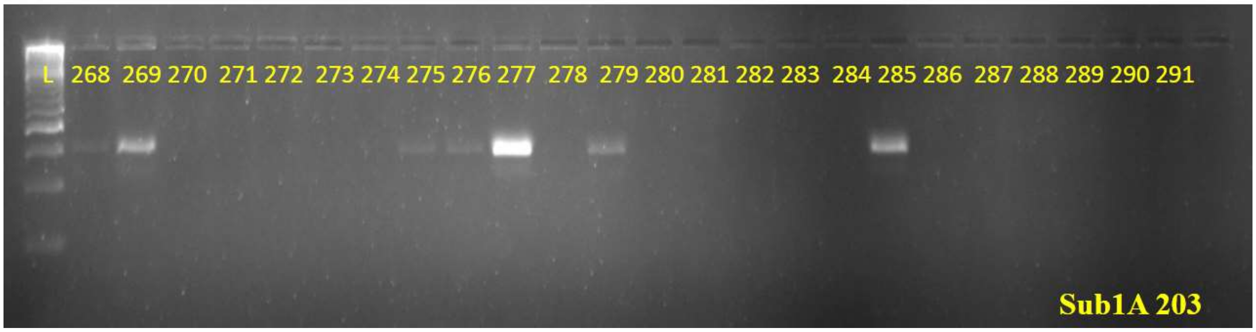
L 268 269 270 271 272 273 274 275 276 277 278 279 280 281 282 283 284 285 286 287 288 289 290 291
Sub1A 203

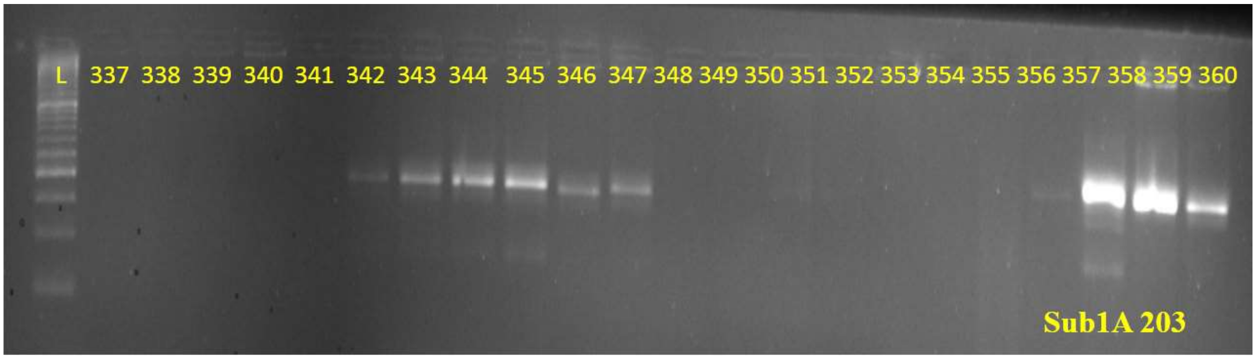
L 337 338 339 340 341 342 343 344 345 346 347 348 349 350 351 352 353 354 355 356 357 358 359 360
Sub1A 203

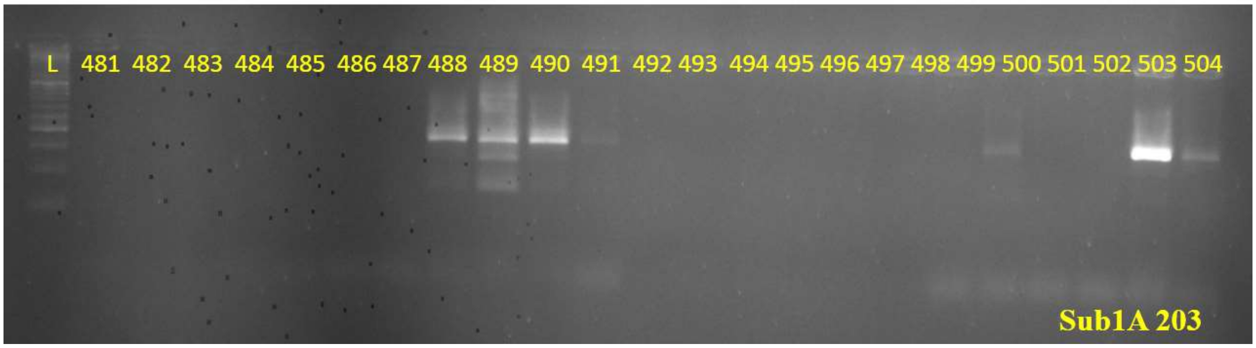
L 481 482 483 484 485 486 487 488 489 490 491 492 493 494 495 496 497 498 499 500 501 502 503 504
Sub1A 203

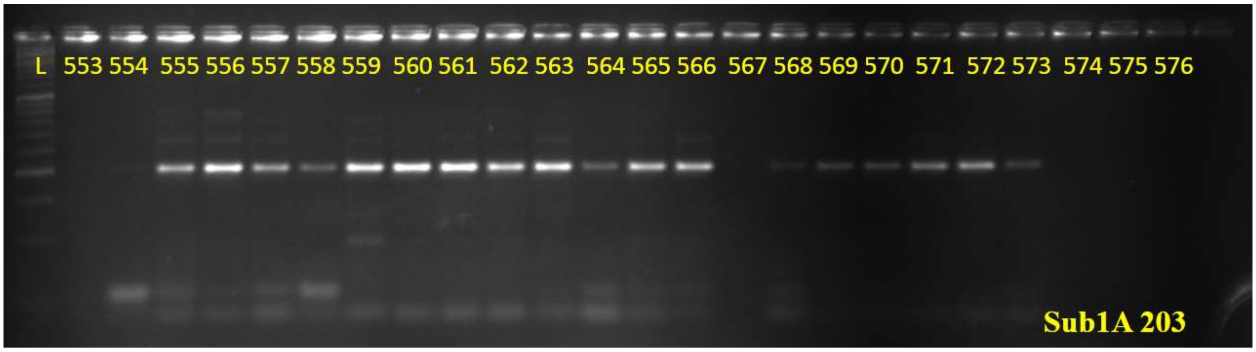
L 553 554 555 556 557 558 559 560 561 562 563 564 565 566 567 568 569 570 571 572 573 574 575 576
Sub1A 203

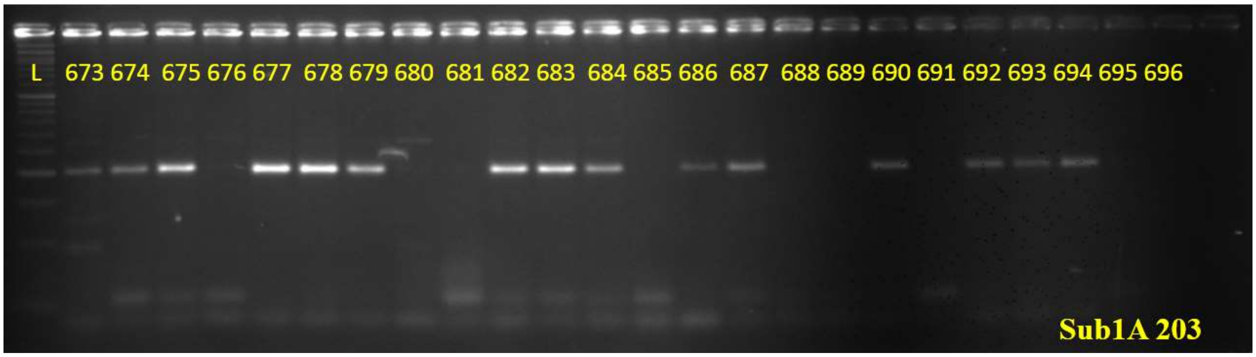
L 673 674 675 676 677 678 679 680 681 682 683 684 685 686 687 688 689 690 691 692 693 694 695 696
Sub1A 203

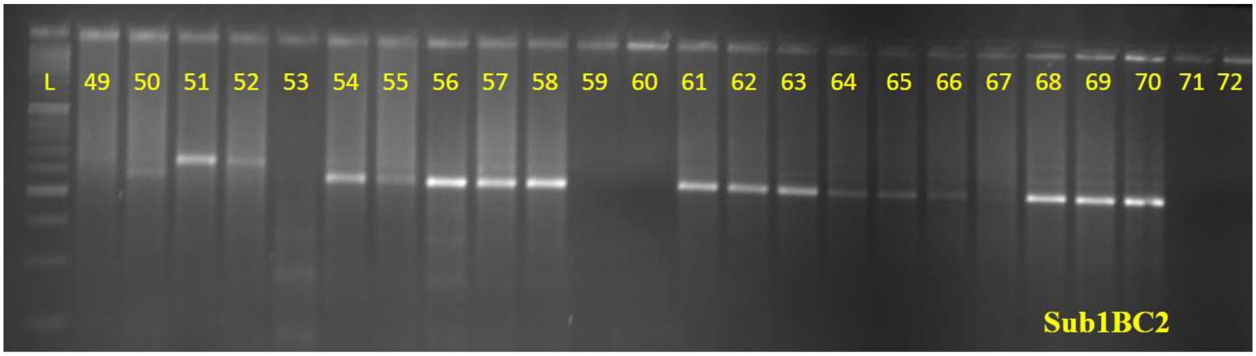
L 49 50 51 52 53 54 55 56 57 58 59 60 61 62 63 64 65 66 67 68 69 70 71 72
Sub1BC2

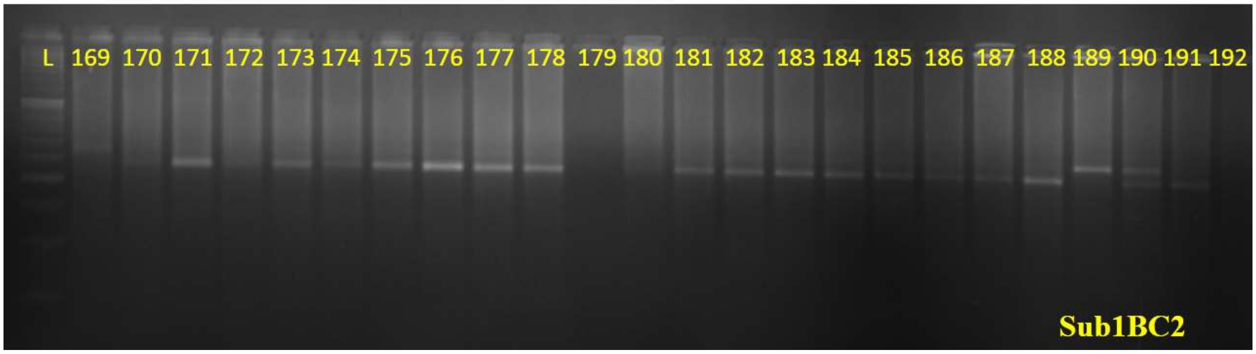
L 169 170 171 172 173 174 175 176 177 178 179 180 181 182 183 184 185 186 187 188 189 190 191 192
Sub1BC2

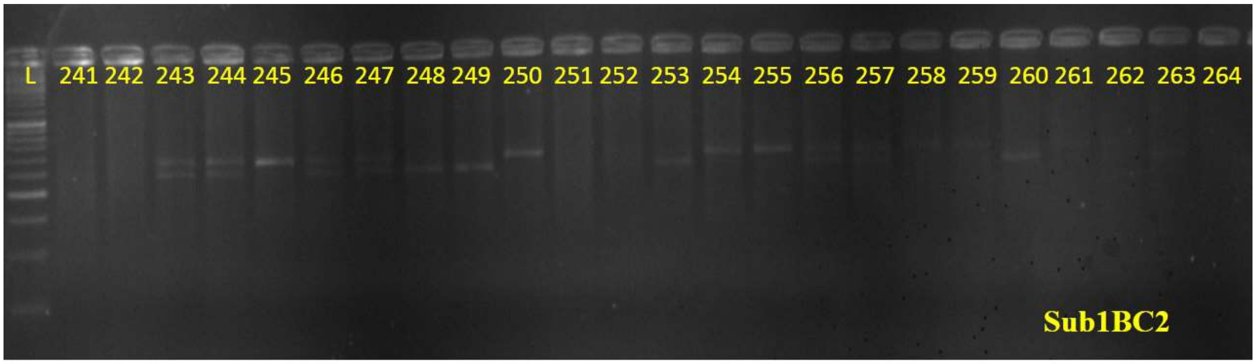
L 241 242 243 244 245 246 247 248 249 250 251 252 253 254 255 256 257 258 259 260 261 262 263 264
Sub1BC2

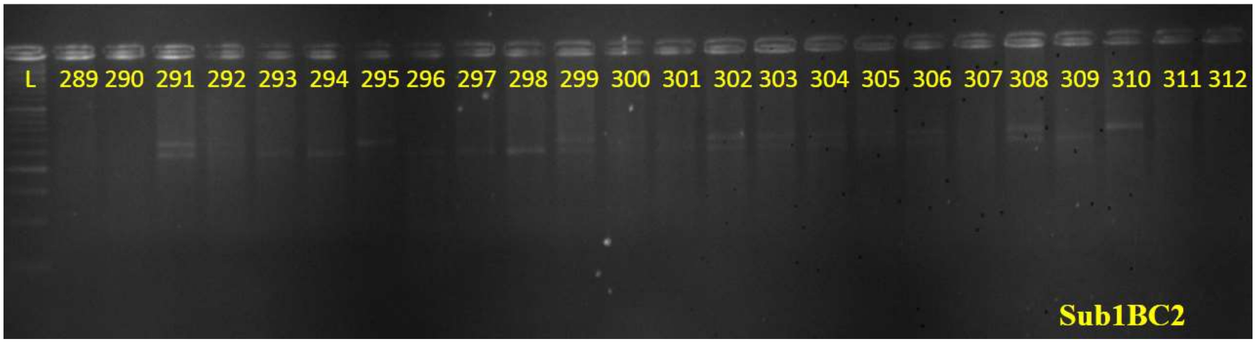
L 289 290 291 292 293 294 295 296 297 298 299 300 301 302 303 304 305 306 307 308 309 310 311 312
Sub1BC2

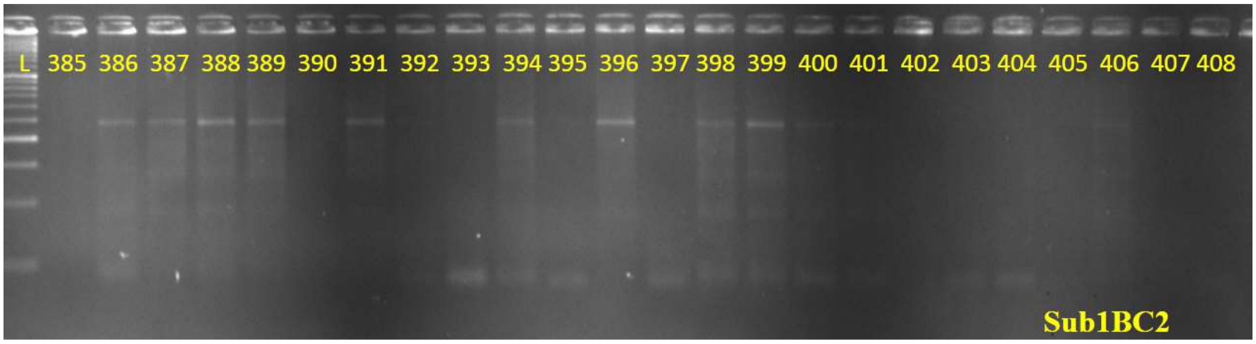
L 385 386 387 388 389 390 391 392 393 394 395 396 397 398 399 400 401 402 403 404 405 406 407 408
Sub1BC2

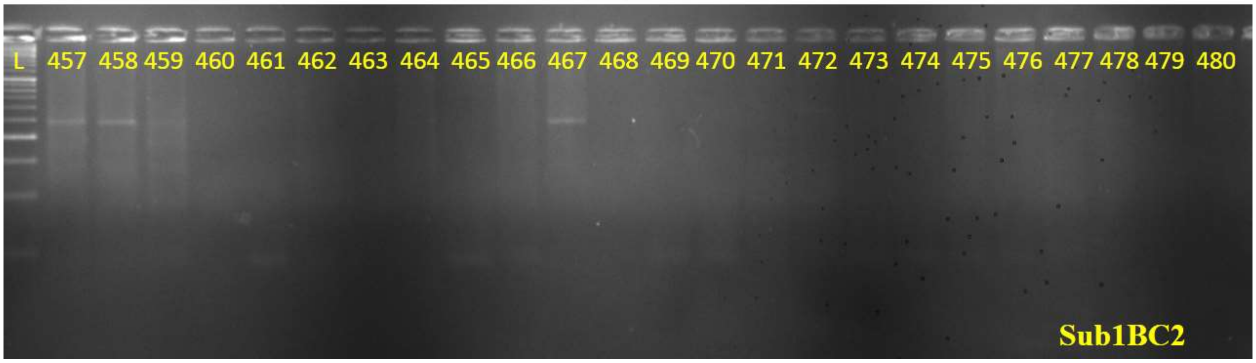
L 457 458 459 460 461 462 463 464 465 466 467 468 469 470 471 472 473 474 475 476 477 478 479 480
Sub1BC2

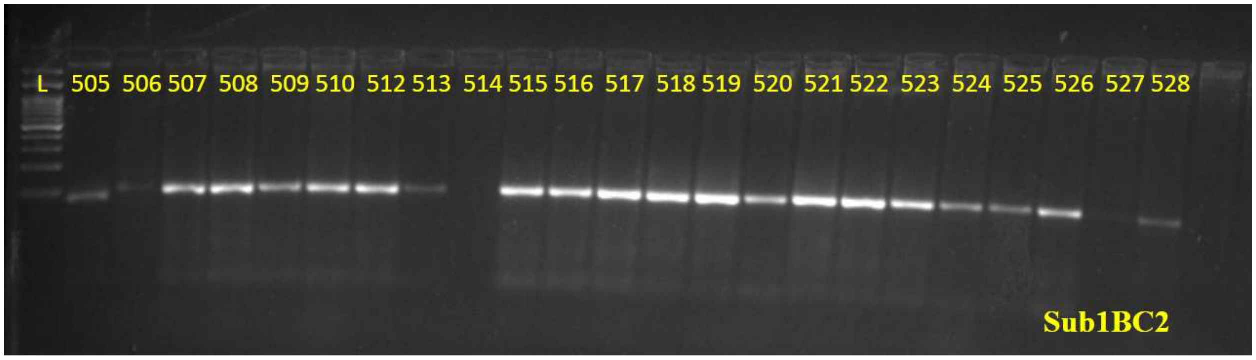
L 505 506 507 508 509 510 512 513 514 515 516 517 518 519 520 521 522 523 524 525 526 527 528
Sub1BC2

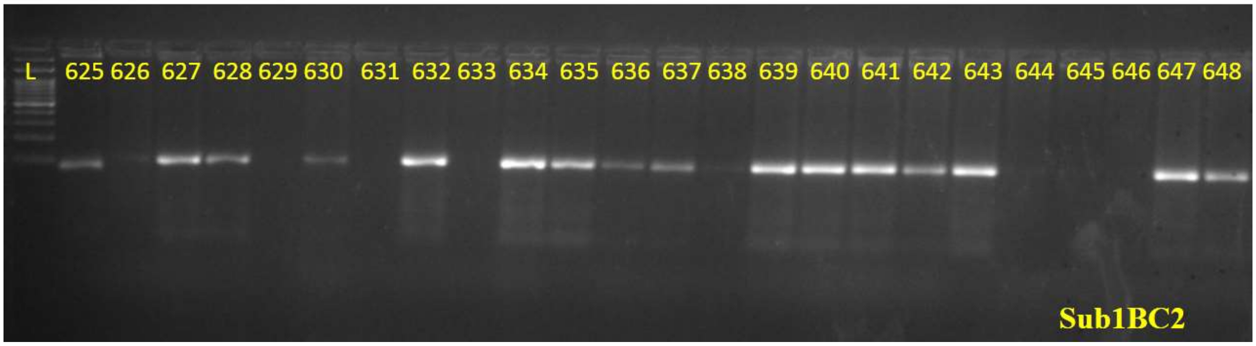
L 625 626 627 628 629 630 631 632 633 634 635 636 637 638 639 640 641 642 643 644 645 646 647 648
Sub1BC2

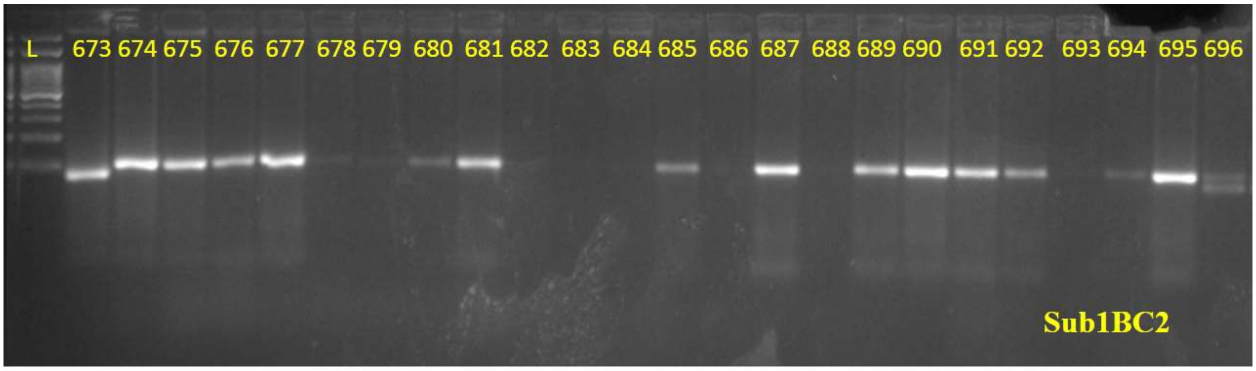
L 673 674 675 676 677 678 679 680 681 682 683 684 685 686 687 688 689 690 691 692 693 694 695 696
Sub1BC2

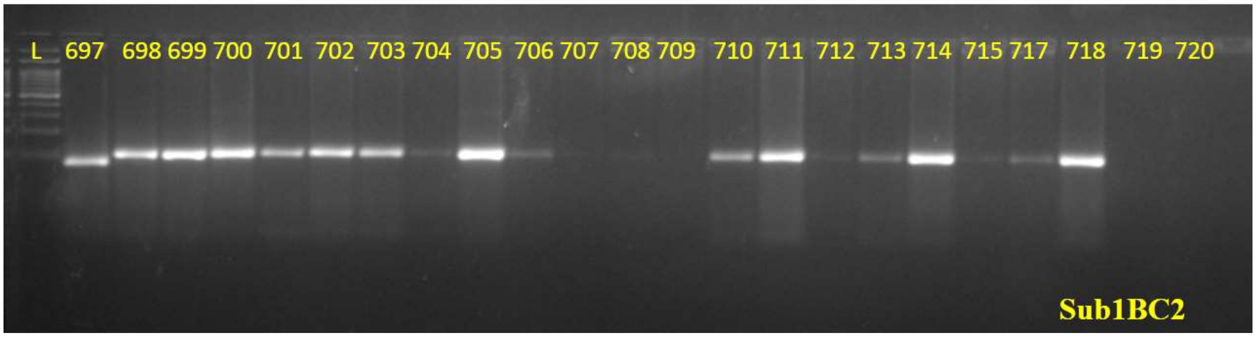
L 697 698 699 700 701 702 703 704 705 706 707 708 709 710 711 712 713 714 715 717 718 719 720
Sub1BC2

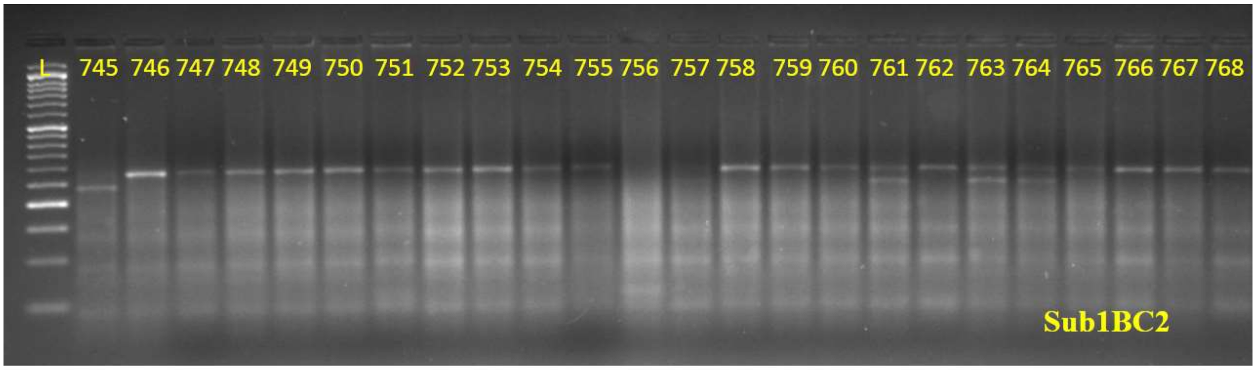
L 745 746 747 748 749 750 751 752 753 754 755 756 757 758 759 760 761 762 763 764 765 766 767 768
Sub1BC2

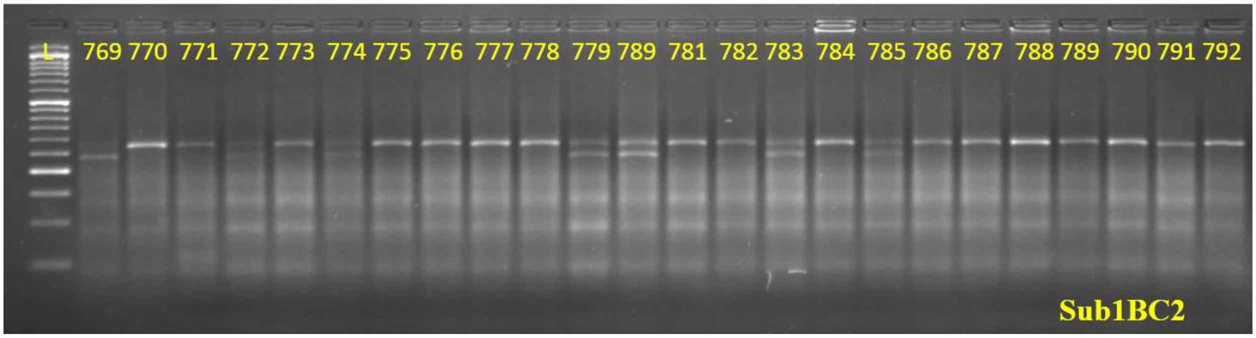
L 769 770 771 772 773 774 775 776 777 778 779 789 781 782 783 784 785 786 787 788 789 790 791 792
Sub1BC2

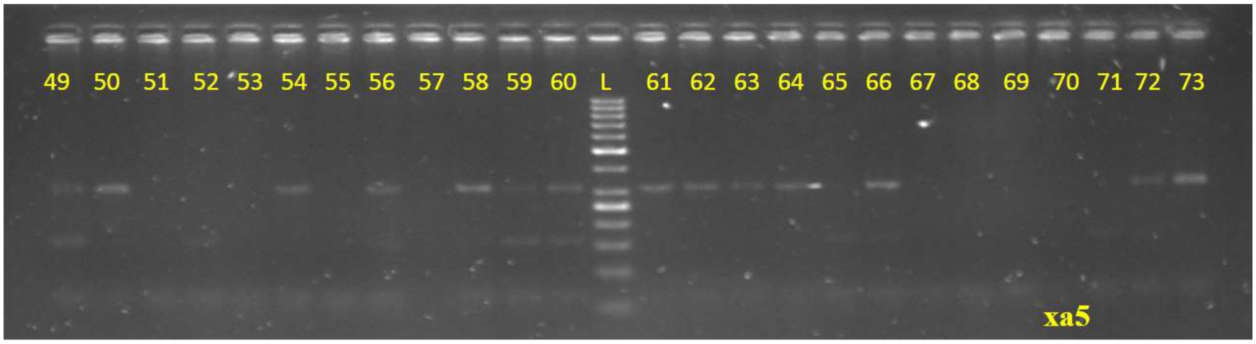
49 50 51 52 53 54 55 56 57 58 59 60 L 61 62 63 64 65 66 67 68 69 70 71 72 73
xa5

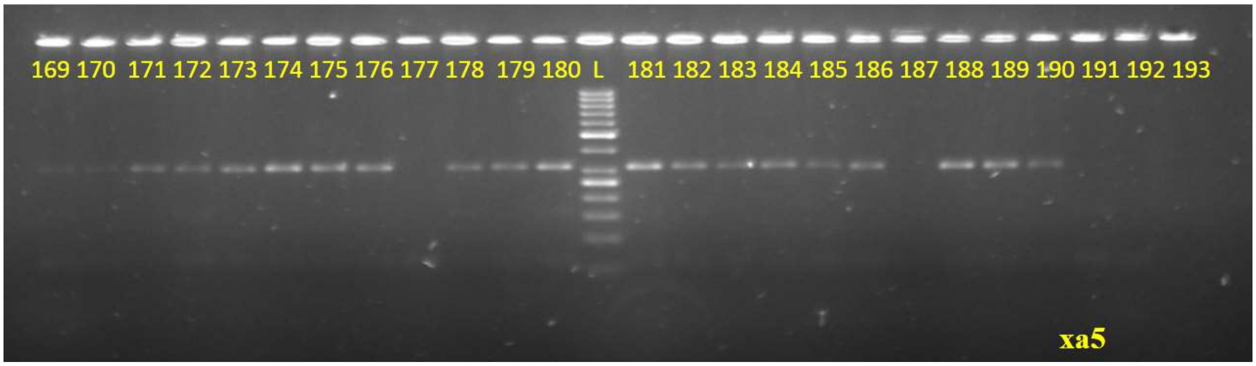
169 170 171 172 173 174 175 176 177 178 179 180 L 181 182 183 184 185 186 187 188 189 190 191 192 193
xa5

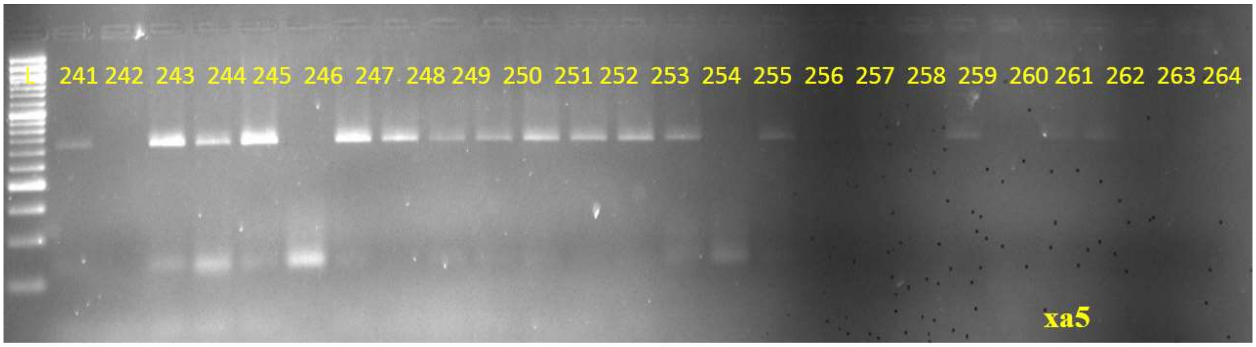
L 241 242 243 244 245 246 247 248 249 250 251 252 253 254 255 256 257 258 259 260 261 262 263 264
xa5

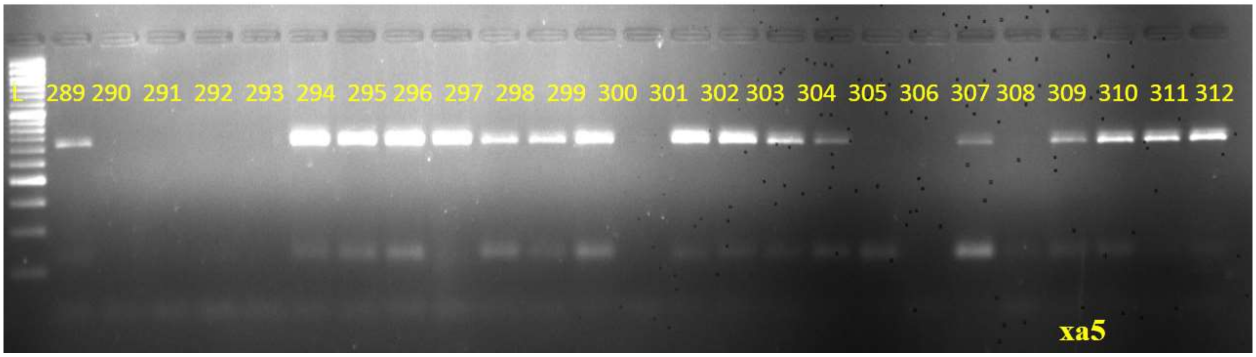
L 289 290 291 292 293 294 295 296 297 298 299 300 301 302 303 304 305 306 307 308 309 310 311 312
xa5

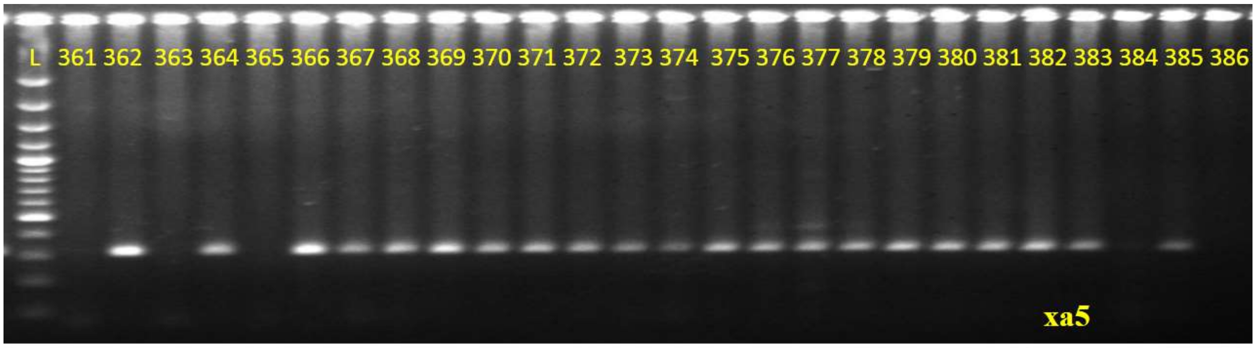
L 361 362 363 364 365 366 367 368 369 370 371 372 373 374 375 376 377 378 379 380 381 382 383 384 385 386
xa5

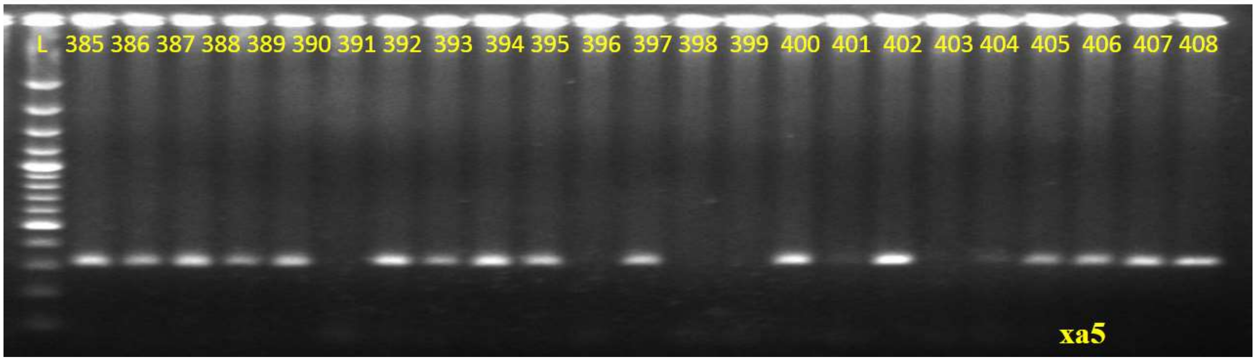
L 385 386 387 388 389 390 391 392 393 394 395 396 397 398 399 400 401 402 403 404 405 406 407 408
xa5

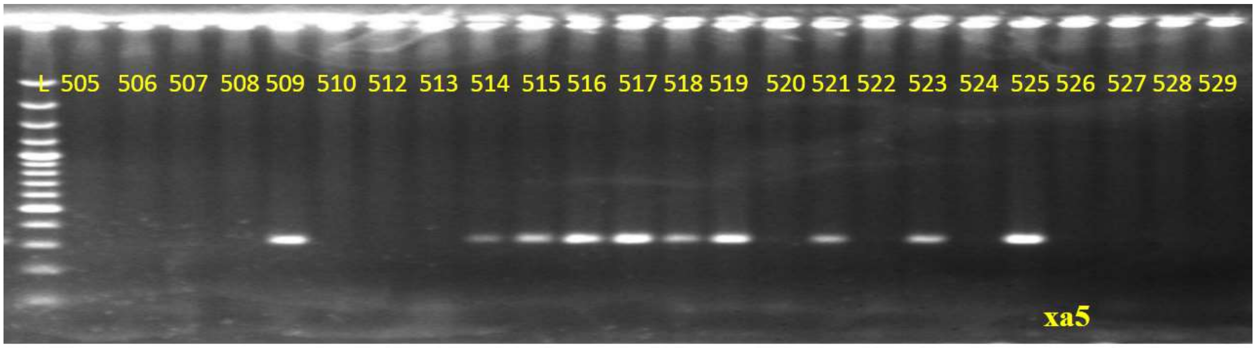
L 505 506 507 508 509 510 512 513 514 515 516 517 518 519 520 521 522 523 524 525 526 527 528 529
xa5

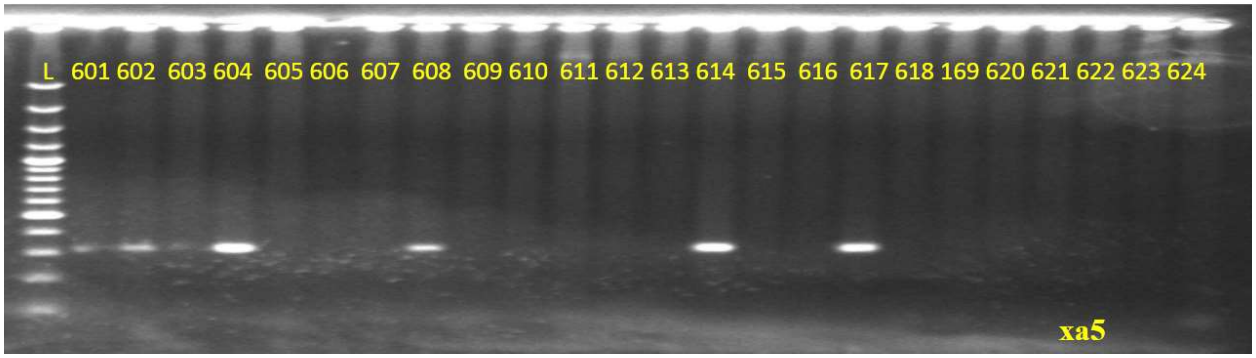
L 601 602 603 604 605 606 607 608 609 610 611 612 613 614 615 616 617 618 169 620 621 622 623 624
xa5

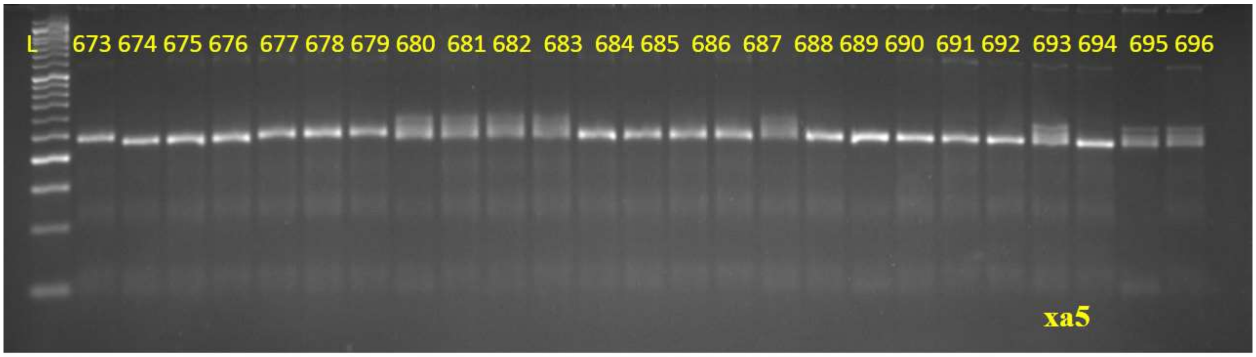
L 673 674 675 676 677 678 679 680 681 682 683 684 685 686 687 688 689 690 691 692 693 694 695 696
xa5

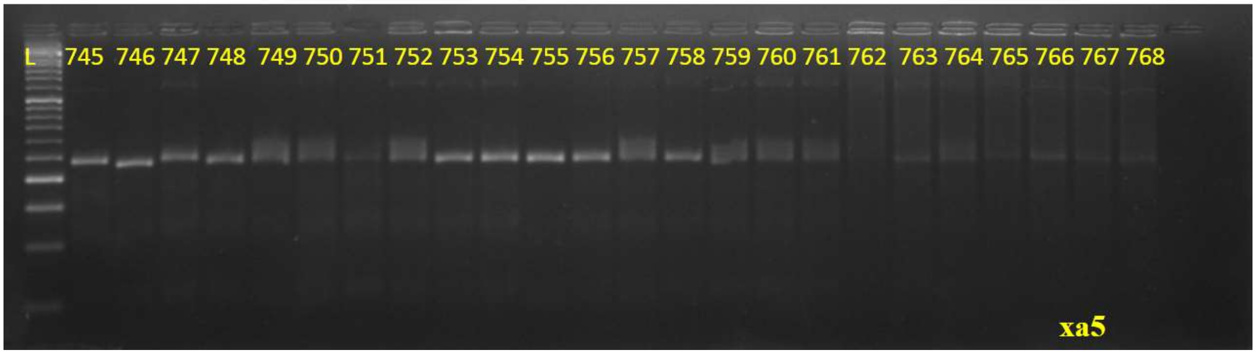
L 745 746 747 748 749 750 751 752 753 754 755 756 757 758 759 760 761 762 763 764 765 766 767 768
xa5

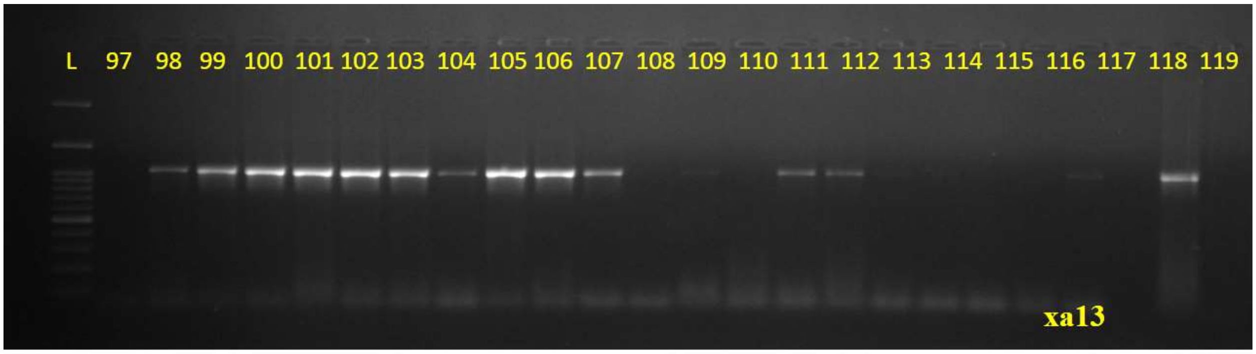
L 97 98 99 100 101 102 103 104 105 106 107 108 109 110 111 112 113 114 115 116 117 118 119
xa13

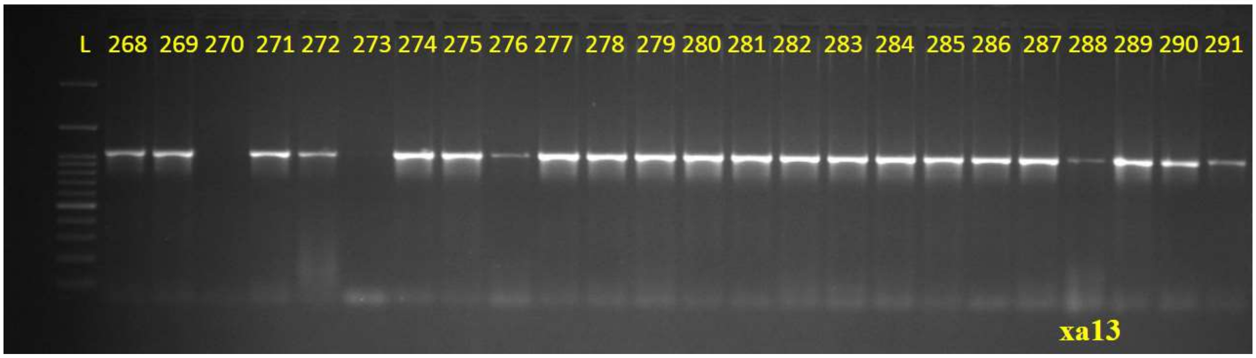
L 268 269 270 271 272 273 274 275 276 277 278 279 280 281 282 283 284 285 286 287 288 289 290 291
xa13

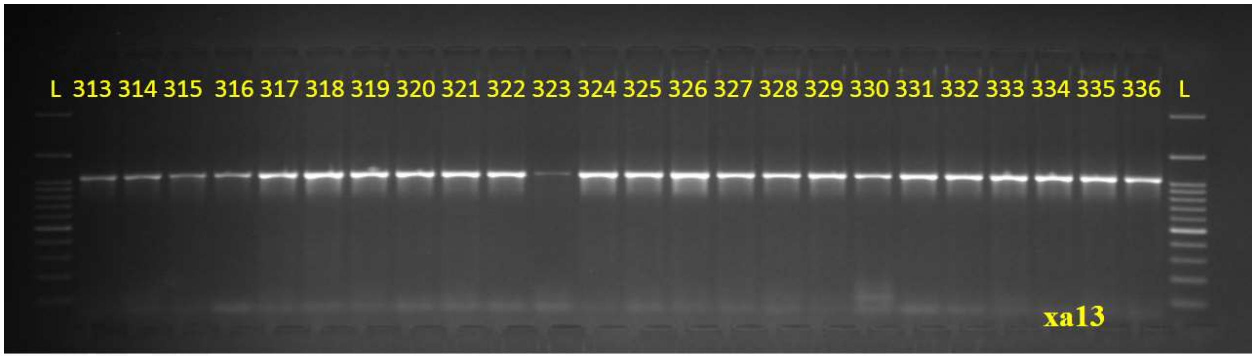
L 313 314 315 316 317 318 319 320 321 322 323 324 325 326 327 328 329 330 331 332 333 334 335 336 L
xa13

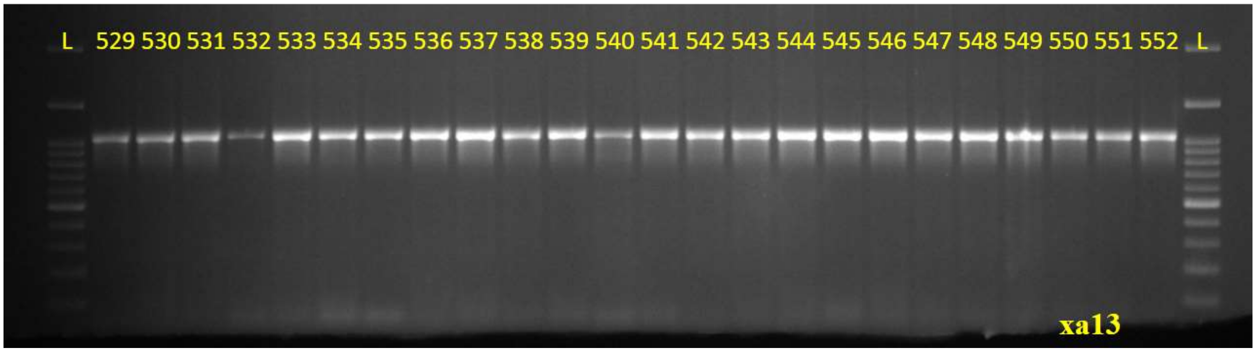
L 529 530 531 532 533 534 535 536 537 538 539 540 541 542 543 544 545 546 547 548 549 550 551 552 L
xa13

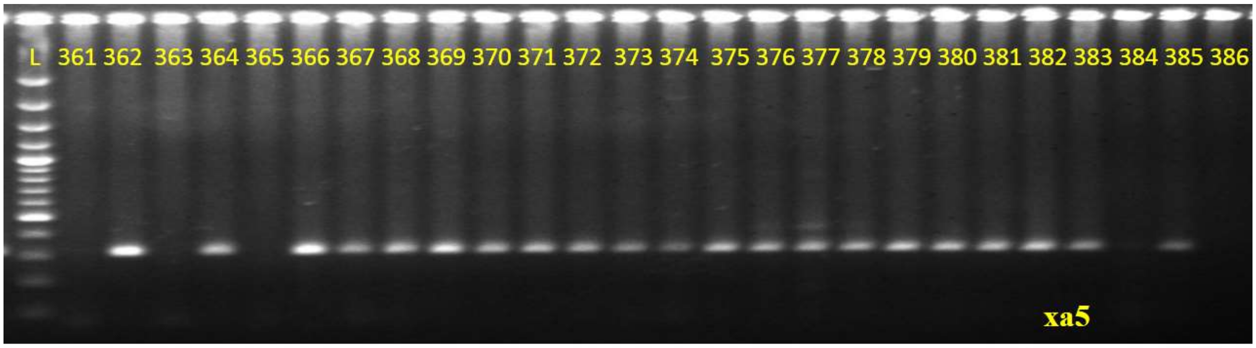
L 361 362 363 364 365 366 367 368 369 370 371 372 373 374 375 376 377 378 379 380 381 382 383 384 385 386
xa5

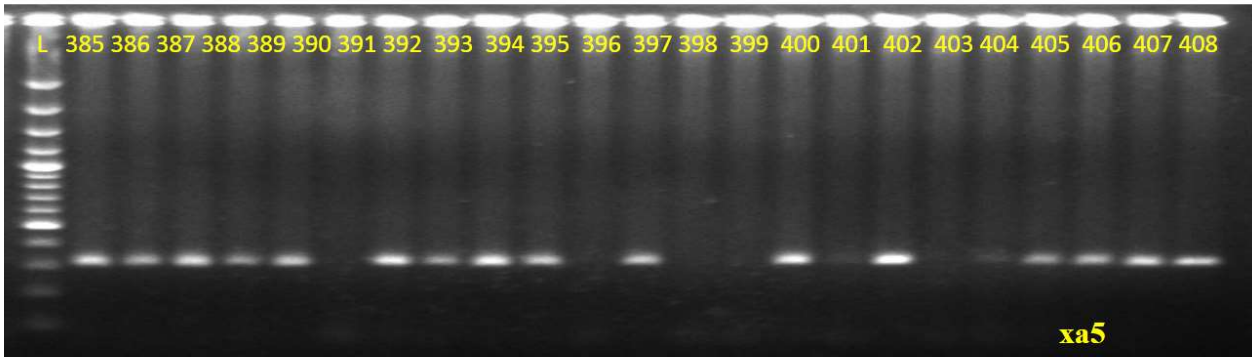
L 385 386 387 388 389 390 391 392 393 394 395 396 397 398 399 400 401 402 403 404 405 406 407 408
xa5

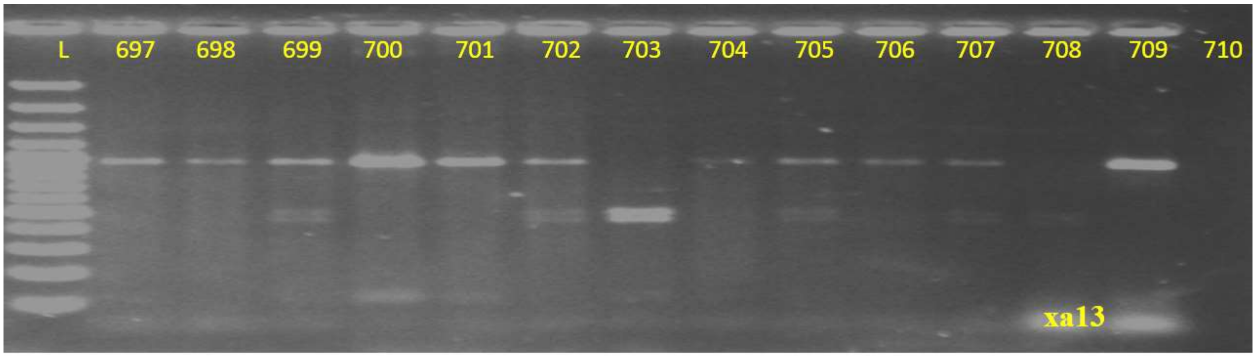
L 697 698 699 700 701 702 703 704 705 706 707 708 709 710
xa13

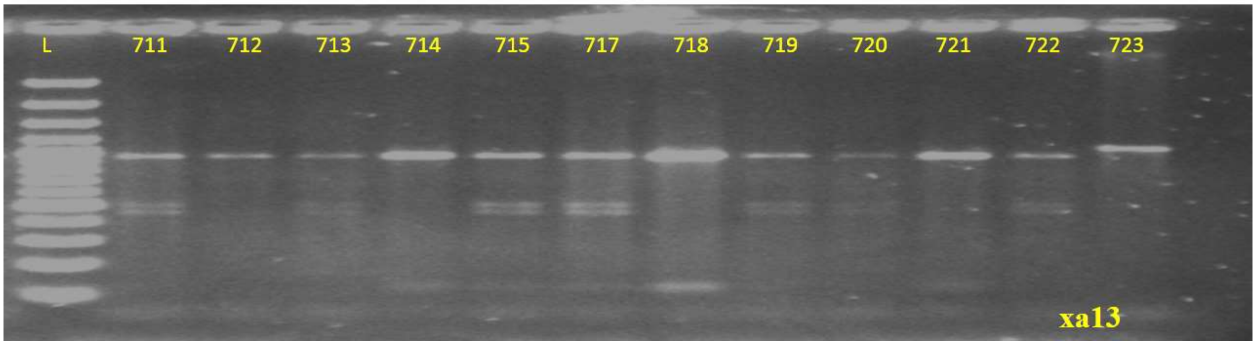
L 711 712 713 714 715 717 718 719 720 721 722 723
xa13

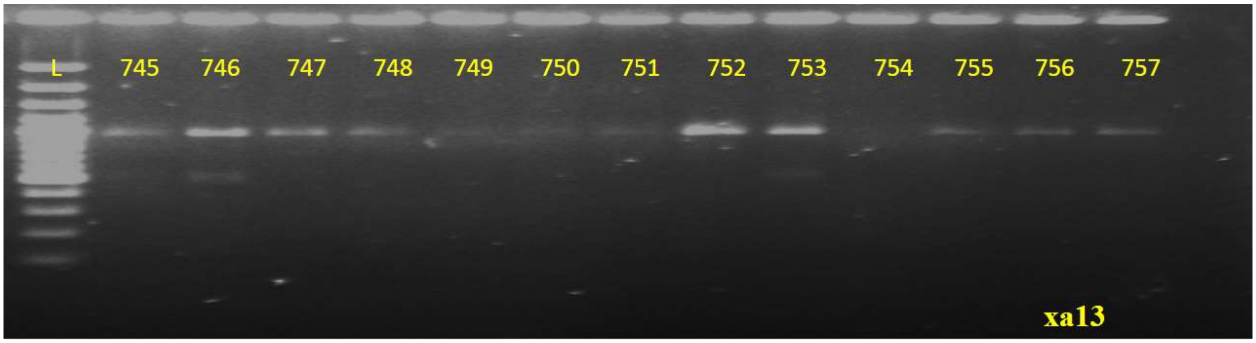
L 745 746 747 748 749 750 751 752 753 754 755 756 757
xa13

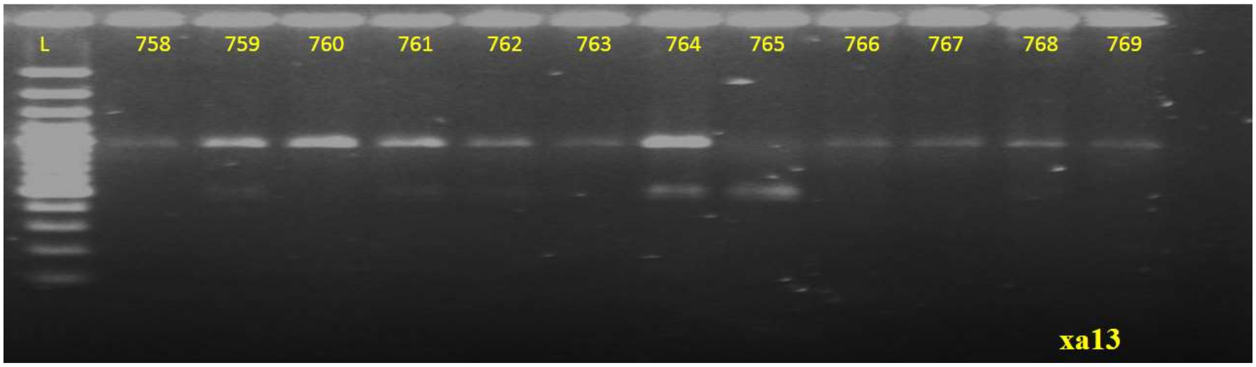
L 758 759 760 761 762 763 764 765 766 767 768 769
xa13

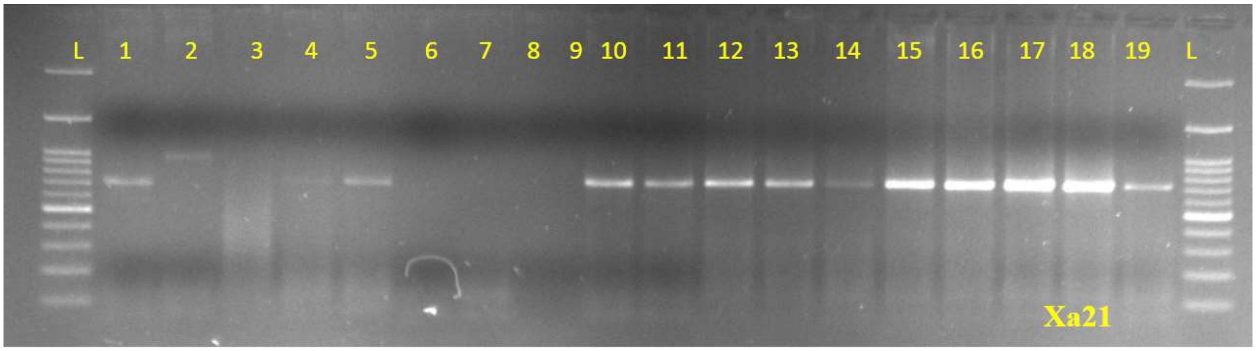
L 1 2 3 4 5 6 7 8 9 10 11 12 13 14 15 16 17 18 19 L
Xa21

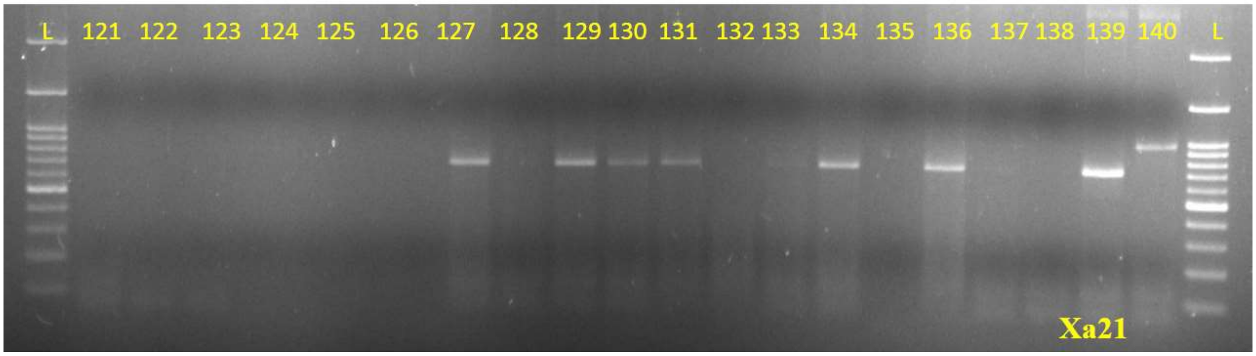
L 121 122 123 124 125 126 127 128 129 130 131 132 133 134 135 136 137 138 139 140 L
Xa21

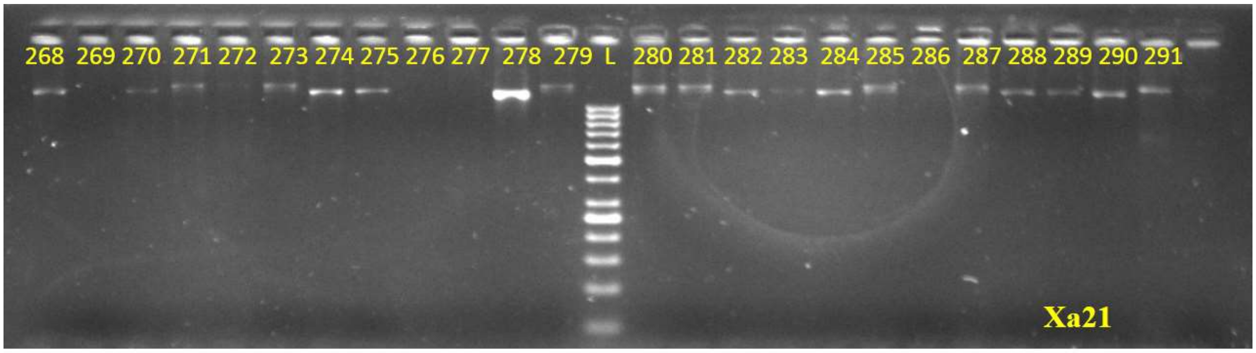
268 269 270 271 272 273 274 275 276 277 278 279 L 280 281 282 283 284 285 286 287 288 289 290 291
Xa21

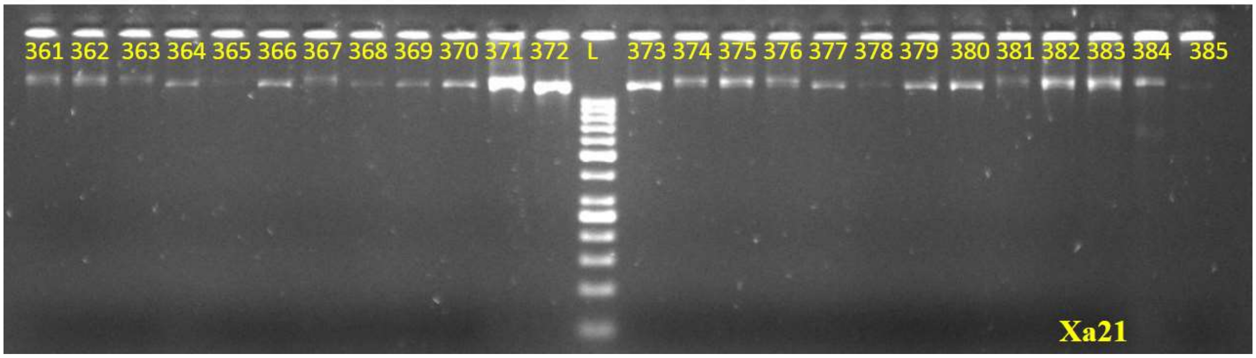
361 362 363 364 365 366 367 368 369 370 371 372 L 373 374 375 376 377 378 379 380 381 382 383 384 385
Xa21

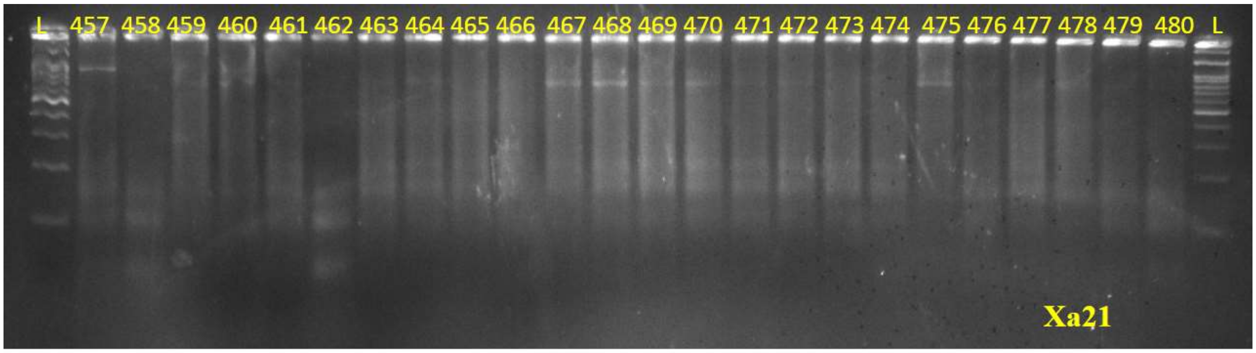
L 457 458 459 460 461 462 463 464 465 466 467 468 469 470 471 472 473 474 475 476 477 478 479 480 L
Xa21

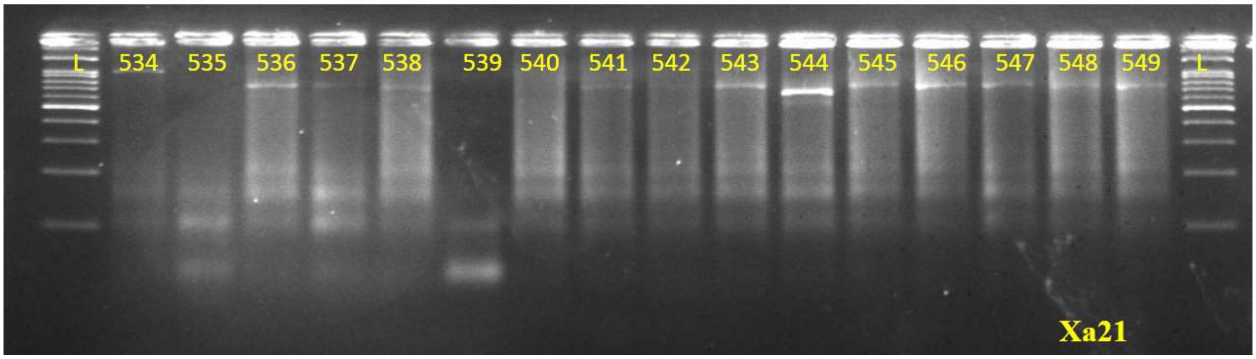
L 534 535 536 537 538 539 540 541 542 543 544 545 546 547 548 549 L
Xa21

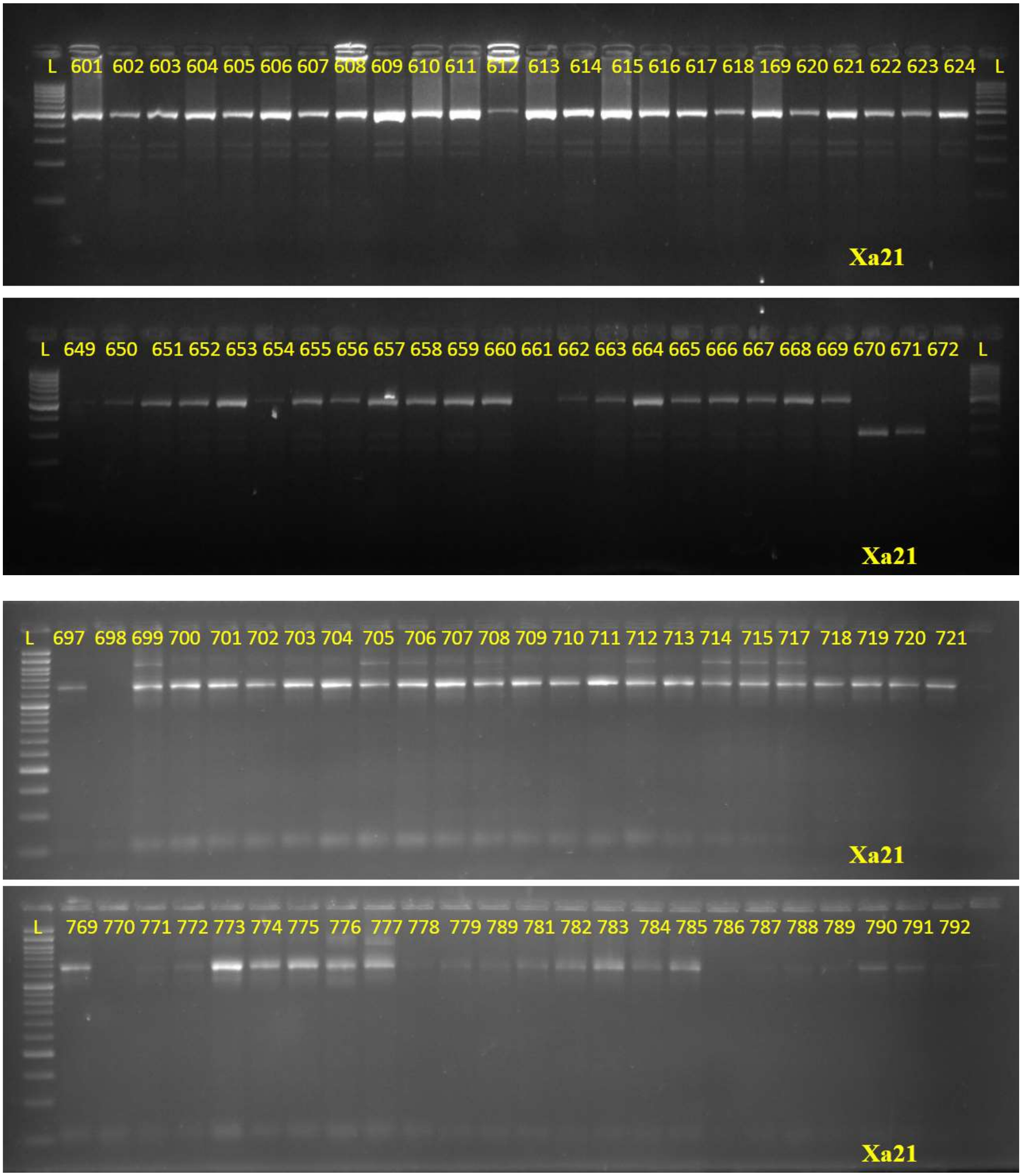

Supplementary figure S7. Molecular screening of BC3F2 progenies in rice variety, Ranidhan background for submergence tolerance and bacterial blight resistance.
